# Correction: Spatial heterogeneity of microbial community structure and its environmental drivers in surface sediments of Erhai Lake

**DOI:** 10.1371/journal.pone.0337922

**Published:** 2025-12-01

**Authors:** Hong Xiang, Cong-Gao Yang, Hao-Qin Xiong, Hao-Ran Bao, Jia-Zhuo Qu, Zhe-Xi Luan, Xiao-Long Sun, Zheng Li

The eighth author’s first and last name are switched and incorrectly spelled. The correct name is: Zheng Li.
