# Supplementary material for: Spatial heterogeneity of microbial community structure and its environmental drivers in surface sediments of Erhai Lake
Source: PLoS One. 2025 Sep 4;20(9):e0322915. doi: 10.1371/journal.pone.0322915 (PMC12410724; doi:10.1371/journal.pone.0322915)
Supplement: S1 Dataset — (DOCX) [file pone.0322915.s001.docx]

Table S1. Community structure of bacteria in water from different sampling points

| Phylum | XHC | YHY | LHQ | KLC | THC | DZ | XYZ | XJY | FMY | BP | LQ | RLYC | TY | SPC | YJC | XC |
| --- | --- | --- | --- | --- | --- | --- | --- | --- | --- | --- | --- | --- | --- | --- | --- | --- |
| Proteobacteria | 16518 | 10131 | 14431 | 23171 | 30966 | 28570 | 25236 | 24413 | 34216 | 28796 | 25288 | 26439 | 27268 | 20464 | 12622 | 21351 |
| (Unassigned) | 5357 | 5670 | 5840 | 4004 | 3462 | 3112 | 4529 | 4660 | 3269 | 4219 | 4741 | 3983 | 4663 | 5515 | 6156 | 4876 |
| Bacteroidota | 6524 | 11129 | 8370 | 11184 | 7338 | 12498 | 9201 | 12564 | 8662 | 7941 | 12268 | 7917 | 6679 | 7037 | 4370 | 11618 |
| Chloroflexi | 6179 | 9475 | 11199 | 7360 | 6883 | 4370 | 5124 | 4697 | 4224 | 4841 | 5705 | 8893 | 9290 | 9326 | 8949 | 4789 |
| Desulfobacterota | 2674 | 10441 | 4497 | 4430 | 2916 | 3801 | 2630 | 4806 | 3018 | 6249 | 5504 | 4892 | 7431 | 6158 | 3307 | 3308 |
| Verrucomicrobiota | 1839 | 2433 | 2343 | 2630 | 2806 | 2471 | 1716 | 2447 | 3756 | 3070 | 3291 | 3217 | 2242 | 2364 | 1915 | 2053 |
| Acidobacteriota | 4210 | 3945 | 3937 | 3810 | 3541 | 3108 | 4330 | 3032 | 2905 | 2825 | 2688 | 4387 | 4618 | 3199 | 6786 | 3653 |
| Actinobacteriota | 3569 | 1553 | 1249 | 4450 | 4770 | 3114 | 1764 | 2631 | 3959 | 2259 | 1618 | 4297 | 2517 | 1282 | 2788 | 2995 |
| Patescibacteria | 551 | 2335 | 752 | 885 | 857 | 902 | 869 | 1177 | 1002 | 727 | 1299 | 841 | 1019 | 1053 | 1819 | 1195 |
| Firmicutes | 2407 | 4361 | 2178 | 1117 | 1191 | 1820 | 935 | 2171 | 1530 | 1138 | 834 | 903 | 1189 | 2766 | 2862 | 540 |
| Myxococcota | 1042 | 1419 | 780 | 1395 | 1176 | 718 | 1810 | 606 | 597 | 667 | 570 | 742 | 1118 | 1119 | 1622 | 1703 |
| Planctomycetota | 620 | 301 | 597 | 638 | 637 | 492 | 937 | 385 | 504 | 449 | 463 | 586 | 409 | 458 | 503 | 915 |
| Latescibacterota | 975 | 483 | 877 | 609 | 400 | 496 | 680 | 329 | 261 | 493 | 446 | 601 | 906 | 922 | 1267 | 839 |
| Spirochaetota | 527 | 2557 | 959 | 911 | 283 | 696 | 565 | 1114 | 249 | 598 | 882 | 486 | 1384 | 1505 | 701 | 358 |
| Nitrospirota | 1509 | 896 | 2016 | 1290 | 392 | 1581 | 1190 | 304 | 548 | 1447 | 630 | 877 | 1143 | 853 | 3176 | 4340 |
| Sva0485 | 1867 | 521 | 3741 | 959 | 578 | 508 | 212 | 369 | 180 | 881 | 1024 | 655 | 1963 | 1573 | 2999 | 1280 |
| Gemmatimonadota | 890 | 416 | 390 | 507 | 902 | 410 | 1670 | 468 | 436 | 290 | 180 | 190 | 278 | 348 | 2446 | 1195 |
| Bdellovibrionota | 176 | 116 | 196 | 242 | 213 | 123 | 291 | 174 | 337 | 117 | 153 | 168 | 128 | 240 | 116 | 325 |
| NB1-j | 911 | 44 | 169 | 307 | 291 | 351 | 1356 | 216 | 157 | 508 | 168 | 151 | 403 | 99 | 414 | 557 |
| Cyanobacteria | 424 | 97 | 100 | 87 | 439 | 686 | 178 | 114 | 290 | 173 | 482 | 236 | 62 | 269 | 34 | 128 |
| MBNT15 | 420 | 441 | 837 | 165 | 207 | 72 | 212 | 93 | 106 | 226 | 110 | 75 | 140 | 97 | 3366 | 590 |
| Dependentiae | 83 | 118 | 126 | 31 | 92 | 27 | 428 | 77 | 34 | 74 | 37 | 55 | 125 | 111 | 167 | 60 |
| Zixibacteria | 200 | 375 | 214 | 208 | 146 | 162 | 289 | 99 | 113 | 130 | 136 | 98 | 342 | 360 | 473 | 177 |
| Nitrospinota | 227 | 303 | 502 | 794 | 78 | 269 | 58 | 154 | 298 | 362 | 408 | 515 | 230 | 203 | 582 | 643 |
| Armatimonadota | 94 | 255 | 116 | 54 | 95 | 108 | 123 | 109 | 101 | 45 | 29 | 140 | 65 | 125 | 314 | 131 |
| Campilobacterota | 342 | 1866 | 37 | 74 | 70 | 106 | 52 | 119 | 99 | 146 | 87 | 224 | 226 | 1540 | 32 | 19 |
| Elusimicrobiota | 79 | 115 | 71 | 62 | 14 | 87 | 35 | 44 | 18 | 31 | 82 | 59 | 20 | 61 | 111 | 124 |
| Methylomirabilota | 127 | 100 | 141 | 88 | 21 | 60 | 72 | 66 | 20 | 34 | 34 | 75 | 117 | 86 | 891 | 38 |
| Fibrobacterota | 44 | 140 | 51 | 150 | 37 | 93 | 63 | 77 | 82 | 63 | 33 | 77 | 59 | 70 | 84 | 75 |
| Sumerlaeota | 9 | 58 | 97 | 17 | 31 | 2 | 43 | 8 | 19 | 32 | 37 | 36 | 64 | 38 | 54 | 25 |
| Calditrichota | 27 | 104 | 331 | 87 | 8 | 33 | 8 | 28 | 0 | 11 | 71 | 26 | 153 | 130 | 62 | 43 |
| Hydrogenedentes | 6 | 34 | 10 | 32 | 41 | 14 | 19 | 10 | 3 | 23 | 56 | 35 | 61 | 32 | 28 | 28 |
| TA06 | 45 | 57 | 94 | 35 | 12 | 17 | 0 | 31 | 8 | 36 | 62 | 43 | 142 | 76 | 14 | 18 |
| Halobacterota | 30 | 319 | 247 | 92 | 106 | 253 | 70 | 147 | 62 | 75 | 168 | 141 | 201 | 278 | 128 | 34 |
| LCP-89 | 23 | 231 | 20 | 40 | 15 | 30 | 0 | 60 | 12 | 16 | 46 | 27 | 129 | 126 | 39 | 22 |
| Cloacimonadota | 3 | 74 | 12 | 36 | 80 | 14 | 10 | 114 | 2 | 45 | 110 | 23 | 59 | 43 | 6 | 3 |
| WS2 | 11 | 90 | 32 | 7 | 17 | 13 | 20 | 18 | 15 | 23 | 16 | 33 | 63 | 22 | 28 | 6 |
| Caldisericota | 29 | 201 | 65 | 45 | 69 | 175 | 26 | 120 | 20 | 93 | 167 | 82 | 61 | 198 | 111 | 20 |
| NKB15 | 6 | 3 | 59 | 3 | 2 | 13 | 6 | 11 | 3 | 5 | 21 | 7 | 116 | 25 | 8 | 6 |
| Modulibacteria | 28 | 208 | 29 | 42 | 0 | 15 | 0 | 13 | 5 | 9 | 0 | 19 | 6 | 60 | 6 | 13 |
| FCPU426 | 6 | 23 | 30 | 23 | 0 | 17 | 13 | 5 | 3 | 9 | 8 | 9 | 2 | 5 | 13 | 8 |
| Fusobacteriota | 47 | 11 | 37 | 15 | 37 | 17 | 0 | 5 | 14 | 40 | 10 | 9 | 0 | 21 | 32 | 0 |
| Deferrisomatota | 26 | 118 | 60 | 22 | 7 | 28 | 6 | 0 | 14 | 23 | 30 | 22 | 2 | 9 | 58 | 227 |
| Deinococcota | 253 | 23 | 24 | 44 | 3 | 570 | 36 | 8 | 345 | 22 | 19 | 35 | 5 | 0 | 40 | 23 |
| Acetothermia | 2 | 13 | 8 | 8 | 3 | 0 | 0 | 7 | 0 | 9 | 21 | 5 | 55 | 64 | 4 | 14 |
| GAL15 | 5 | 6 | 6 | 3 | 4 | 7 | 0 | 0 | 2 | 0 | 0 | 0 | 0 | 0 | 498 | 3 |
| Fermentibacterota | 0 | 22 | 5 | 0 | 0 | 13 | 3 | 22 | 3 | 5 | 10 | 26 | 18 | 10 | 2 | 0 |
| WPS-2 | 25 | 11 | 6 | 8 | 7 | 16 | 15 | 4 | 42 | 14 | 8 | 15 | 0 | 0 | 3 | 30 |
| WOR-1 | 4 | 14 | 0 | 3 | 0 | 5 | 0 | 3 | 0 | 4 | 5 | 0 | 14 | 16 | 4 | 0 |
| WS1 | 0 | 10 | 2 | 2 | 4 | 2 | 4 | 0 | 2 | 3 | 0 | 8 | 0 | 5 | 5 | 0 |
| WS4 | 6 | 10 | 5 | 0 | 0 | 0 | 4 | 8 | 2 | 5 | 0 | 0 | 0 | 2 | 11 | 0 |
| RCP2-54 | 21 | 9 | 9 | 37 | 29 | 11 | 13 | 11 | 0 | 3 | 0 | 8 | 13 | 3 | 176 | 0 |
| Thermoplasmatota | 0 | 7 | 13 | 0 | 0 | 10 | 0 | 0 | 0 | 8 | 0 | 0 | 0 | 19 | 2 | 0 |
| Entotheonellaeota | 4 | 0 | 0 | 11 | 7 | 0 | 0 | 10 | 0 | 5 | 0 | 0 | 3 | 0 | 19 | 0 |
| Euryarchaeota | 0 | 3 | 4 | 0 | 0 | 0 | 7 | 0 | 6 | 0 | 2 | 8 | 2 | 0 | 0 | 0 |
| uncultured | 0 | 2 | 3 | 3 | 0 | 0 | 4 | 0 | 0 | 4 | 2 | 0 | 5 | 0 | 0 | 0 |
| Margulisbacteria | 0 | 0 | 5 | 5 | 0 | 2 | 0 | 32 | 2 | 0 | 0 | 0 | 0 | 0 | 5 | 0 |
| Crenarchaeota | 2 | 0 | 21 | 0 | 4 | 2 | 0 | 0 | 2 | 0 | 2 | 4 | 0 | 4 | 5 | 0 |
| Dadabacteria | 5 | 0 | 0 | 2 | 2 | 6 | 6 | 0 | 0 | 0 | 0 | 0 | 2 | 0 | 16 | 2 |
| Firestonebacteria | 0 | 4 | 0 | 7 | 0 | 2 | 0 | 0 | 0 | 0 | 0 | 0 | 0 | 0 | 7 | 0 |
| Synergistota | 0 | 6 | 0 | 0 | 0 | 0 | 0 | 0 | 0 | 0 | 0 | 0 | 3 | 7 | 0 | 0 |
| Caldatribacteriota | 0 | 9 | 0 | 0 | 0 | 2 | 0 | 0 | 0 | 0 | 0 | 0 | 0 | 8 | 25 | 0 |
| Desantisbacteria | 0 | 7 | 4 | 0 | 0 | 0 | 0 | 0 | 0 | 0 | 0 | 0 | 0 | 2 | 5 | 0 |
| Asgardarchaeota | 0 | 6 | 8 | 0 | 0 | 0 | 0 | 0 | 0 | 0 | 0 | 0 | 5 | 2 | 0 | 0 |
| Poribacteria | 0 | 3 | 0 | 0 | 0 | 0 | 0 | 2 | 0 | 0 | 0 | 0 | 2 | 0 | 5 | 0 |
| Abditibacteriota | 0 | 0 | 2 | 0 | 0 | 0 | 0 | 0 | 0 | 0 | 0 | 2 | 0 | 2 | 0 | 0 |
| Schekmanbacteria | 0 | 0 | 0 | 0 | 0 | 0 | 0 | 0 | 0 | 0 | 0 | 0 | 0 | 4 | 23 | 0 |
| Edwardsbacteria | 0 | 0 | 6 | 0 | 0 | 0 | 0 | 0 | 0 | 0 | 0 | 0 | 0 | 9 | 0 | 0 |
| CK-2C2-2 | 5 | 0 | 0 | 0 | 0 | 0 | 0 | 0 | 0 | 2 | 0 | 0 | 0 | 0 | 0 | 0 |
| Aenigmarchaeota | 5 | 2 | 0 | 0 | 0 | 0 | 0 | 0 | 0 | 0 | 0 | 0 | 4 | 0 | 0 | 0 |
| Micrarchaeota | 0 | 2 | 0 | 0 | 0 | 0 | 0 | 0 | 0 | 0 | 0 | 0 | 0 | 0 | 0 | 0 |
| MAT-CR-M4-B07 | 2 | 0 | 0 | 0 | 0 | 0 | 0 | 0 | 0 | 0 | 0 | 0 | 0 | 0 | 0 | 0 |
| FW113 | 0 | 0 | 0 | 0 | 0 | 0 | 2 | 0 | 0 | 0 | 0 | 0 | 0 | 0 | 0 | 0 |
| Thermotogota | 0 | 0 | 0 | 0 | 0 | 0 | 0 | 0 | 0 | 0 | 0 | 0 | 0 | 6 | 0 | 0 |
| DTB120 | 0 | 0 | 0 | 0 | 0 | 0 | 0 | 0 | 2 | 0 | 0 | 0 | 0 | 0 | 0 | 0 |

Table S2. Community structure of fungi in water from different sampling points

| Phylum | XHC | YHY | LHQ | KLC | THC | DZ | XYZ | XJY | FMY | BP | LQ | RLYC | TY | SPC | YJC | XC |
| --- | --- | --- | --- | --- | --- | --- | --- | --- | --- | --- | --- | --- | --- | --- | --- | --- |
| unidentified | 13913 | 4499 | 9409 | 3220 | 4253 | 6732 | 3863 | 9532 | 8024 | 5100 | 7185 | 7281 | 7064 | 2952 | 4718 | 15613 |
| Rozellomycota | 162 | 10110 | 4350 | 4211 | 4118 | 1966 | 1434 | 4372 | 2331 | 4578 | 3501 | 3345 | 8483 | 3573 | 1988 | 3134 |
| Ascomycota | 614 | 8811 | 2536 | 8450 | 6019 | 5499 | 5649 | 4494 | 1660 | 3037 | 1685 | 3032 | 3362 | 6029 | 2961 | 1613 |
| Ciliophora | 1632 | 14466 | 5387 | 9996 | 11160 | 14964 | 15662 | 4071 | 16808 | 7485 | 8082 | 15352 | 17684 | 9934 | 11495 | 4265 |
| (Unassigned) | 357 | 1846 | 1674 | 2654 | 1181 | 1337 | 813 | 2633 | 3635 | 2175 | 1548 | 1650 | 1980 | 891 | 949 | 1811 |
| Chytridiomycota | 1068 | 829 | 1371 | 1110 | 298 | 533 | 555 | 615 | 630 | 1437 | 1134 | 2319 | 755 | 246 | 1772 | 1022 |
| Basidiomycota | 209 | 1221 | 310 | 1770 | 332 | 748 | 407 | 626 | 132 | 540 | 118 | 1040 | 264 | 683 | 1786 | 86 |
| Cercozoa | 56 | 849 | 128 | 650 | 92 | 66 | 168 | 468 | 40 | 125 | 130 | 99 | 97 | 289 | 341 | 98 |
| Ochrophyta | 18 | 338 | 40 | 39 | 353 | 22 | 175 | 200 | 19 | 230 | 1309 | 97 | 232 | 151 | 829 | 4 |
| Bacillariophyta | 75 | 80 | 437 | 83 | 3 | 378 | 52 | 498 | 243 | 388 | 1016 | 70 | 547 | 143 | 573 | 46 |
| Ichthyosporia_phy_Incertae_sedis | 23 | 32 | 120 | 390 | 150 | 84 | 31 | 302 | 102 | 337 | 194 | 122 | 0 | 140 | 33 | 40 |
| Mortierellomycota | 11 | 0 | 0 | 83 | 48 | 148 | 72 | 112 | 0 | 9 | 7 | 49 | 59 | 2 | 0 | 0 |
| Monoblepharomycota | 0 | 8 | 20 | 17 | 5 | 0 | 48 | 85 | 11 | 3 | 20 | 17 | 30 | 63 | 0 | 40 |
| Arthropoda | 0 | 233 | 2 | 0 | 194 | 15 | 115 | 0 | 88 | 0 | 0 | 394 | 2304 | 5 | 10 | 10 |
| Glomeromycota | 0 | 0 | 0 | 192 | 0 | 0 | 0 | 3 | 4 | 0 | 5 | 0 | 0 | 0 | 11 | 0 |
| Annelida | 0 | 0 | 0 | 11 | 14 | 101 | 0 | 0 | 3 | 0 | 0 | 12 | 0 | 0 | 0 | 164 |
| Aphelidiomycota | 11 | 2 | 0 | 9 | 0 | 7 | 10 | 0 | 0 | 0 | 23 | 8 | 2 | 0 | 51 | 3 |
| Mucoromycota | 2 | 18 | 61 | 4 | 0 | 9 | 0 | 7 | 10 | 22 | 0 | 0 | 0 | 0 | 0 | 0 |
| Apicomplexa | 2 | 0 | 0 | 0 | 0 | 37 | 47 | 0 | 35 | 0 | 48 | 0 | 0 | 5 | 0 | 0 |
| Nematoda | 0 | 0 | 3 | 0 | 29 | 2 | 739 | 0 | 0 | 0 | 0 | 0 | 0 | 0 | 0 | 0 |
| Plasmodiophoromycota | 0 | 0 | 0 | 0 | 0 | 0 | 0 | 16 | 0 | 10 | 26 | 3 | 6 | 0 | 0 | 0 |
| Kickxellomycota | 4 | 2 | 0 | 20 | 0 | 18 | 0 | 4 | 2 | 0 | 0 | 2 | 0 | 0 | 0 | 20 |
| Rotifera | 0 | 0 | 0 | 0 | 0 | 0 | 0 | 0 | 0 | 0 | 0 | 0 | 2 | 6 | 0 | 25 |
| Gastrotricha | 0 | 0 | 0 | 0 | 8 | 2 | 0 | 16 | 0 | 3 | 0 | 0 | 0 | 0 | 0 | 0 |
| Discosea | 0 | 0 | 0 | 0 | 5 | 0 | 0 | 7 | 2 | 0 | 0 | 0 | 0 | 0 | 0 | 0 |
| Basidiobolomycota | 0 | 0 | 0 | 0 | 0 | 0 | 6 | 0 | 0 | 4 | 0 | 0 | 0 | 0 | 0 | 0 |
| Platyhelminthes | 0 | 0 | 0 | 0 | 0 | 14 | 0 | 0 | 0 | 7 | 0 | 0 | 0 | 0 | 0 | 0 |
| Porifera | 0 | 0 | 35 | 0 | 0 | 0 | 0 | 0 | 0 | 0 | 0 | 0 | 0 | 0 | 0 | 0 |
| Cnidaria | 0 | 0 | 0 | 0 | 0 | 0 | 0 | 0 | 0 | 4 | 0 | 0 | 0 | 0 | 0 | 2 |
| Blastocladiomycota | 0 | 0 | 0 | 2 | 0 | 0 | 0 | 0 | 0 | 0 | 2 | 0 | 0 | 0 | 0 | 0 |
| Olpidiomycota | 0 | 5 | 0 | 0 | 0 | 2 | 0 | 0 | 0 | 0 | 0 | 0 | 0 | 0 | 0 | 0 |
| Dinophyta | 0 | 0 | 0 | 0 | 4 | 0 | 8 | 5 | 0 | 0 | 0 | 0 | 9 | 16 | 0 | 0 |
| Mollusca | 0 | 0 | 0 | 0 | 0 | 11 | 0 | 2 | 0 | 0 | 0 | 0 | 0 | 0 | 0 | 0 |
| Haplosporidia | 0 | 0 | 0 | 0 | 0 | 0 | 0 | 0 | 0 | 0 | 0 | 0 | 0 | 11 | 0 | 0 |
| Heterolobosa_phy_Incertae_sedis | 0 | 5 | 0 | 2 | 0 | 0 | 0 | 0 | 0 | 0 | 0 | 0 | 0 | 0 | 0 | 0 |
| Oomycota | 0 | 0 | 0 | 0 | 0 | 0 | 0 | 0 | 4 | 0 | 0 | 0 | 0 | 0 | 0 | 0 |
| Cryptophyta | 0 | 0 | 0 | 0 | 0 | 0 | 0 | 0 | 0 | 0 | 0 | 9 | 0 | 0 | 0 | 0 |
| Euglenozoa_phy_Incertae_sedis | 0 | 7 | 0 | 0 | 0 | 0 | 0 | 0 | 0 | 0 | 0 | 0 | 0 | 0 | 0 | 0 |

Table S3.bacterial genus in surface sediments of Erhai Lake

| Genus | XHC | YHY | THC | DZ | XYZ | XJZ | FMY | BP | LQ | RLYC | TY | SPC | YJC | XC | LHQ | KLC |
| --- | --- | --- | --- | --- | --- | --- | --- | --- | --- | --- | --- | --- | --- | --- | --- | --- |
| Haliangium | 156 | 161 | 130 | 113 | 390 | 77 | 82 | 47 | 37 | 56 | 71 | 177 | 200 | 237 | 82 | 154 |
| Geobacter | 97 | 559 | 357 | 252 | 100 | 711 | 279 | 321 | 572 | 321 | 258 | 902 | 393 | 109 | 568 | 282 |
| Spirochaeta_2 | 201 | 300 | 86 | 234 | 100 | 278 | 61 | 202 | 461 | 144 | 453 | 843 | 248 | 142 | 593 | 253 |
| Anaerolinea | 140 | 741 | 367 | 369 | 275 | 301 | 254 | 211 | 186 | 537 | 214 | 273 | 207 | 51 | 154 | 292 |
| Bdellovibrio | 49 | 7 | 65 | 35 | 77 | 62 | 90 | 56 | 66 | 69 | 22 | 44 | 20 | 152 | 80 | 103 |
| Luteolibacter | 140 | 117 | 614 | 752 | 94 | 453 | 2030 | 833 | 694 | 596 | 210 | 132 | 83 | 129 | 154 | 177 |
| Coxiella | 40 | 164 | 35 | 40 | 26 | 52 | 21 | 49 | 57 | 55 | 170 | 61 | 89 | 47 | 164 | 54 |
| RBG-16-49-21 | 57 | 542 | 92 | 135 | 54 | 346 | 57 | 76 | 183 | 100 | 347 | 197 | 60 | 52 | 93 | 98 |
| Candidatus_Competibacter | 127 | 7 | 137 | 243 | 114 | 138 | 88 | 489 | 1053 | 367 | 1126 | 84 | 79 | 214 | 121 | 741 |
| SM1A02 | 83 | 30 | 144 | 119 | 203 | 100 | 151 | 85 | 84 | 118 | 63 | 29 | 27 | 210 | 52 | 107 |
| Syntrophorhabdus | 33 | 181 | 299 | 115 | 58 | 347 | 116 | 188 | 313 | 118 | 310 | 358 | 34 | 28 | 190 | 123 |
| Desulfatiglans | 464 | 160 | 48 | 304 | 91 | 133 | 122 | 339 | 645 | 255 | 1008 | 521 | 301 | 727 | 533 | 437 |
| Flavobacterium | 22 | 65 | 193 | 517 | 55 | 252 | 308 | 77 | 63 | 66 | 93 | 41 | 21 | 34 | 90 | 130 |
| Ellin6067 | 341 | 96 | 453 | 565 | 407 | 531 | 538 | 551 | 567 | 570 | 348 | 177 | 218 | 1348 | 497 | 614 |
| Anaeromyxobacter | 137 | 85 | 382 | 148 | 50 | 139 | 74 | 107 | 91 | 104 | 77 | 169 | 482 | 246 | 183 | 178 |
| Bryobacter | 235 | 153 | 187 | 204 | 322 | 143 | 88 | 106 | 41 | 198 | 88 | 47 | 93 | 255 | 51 | 295 |
| Syntrophus | 46 | 333 | 117 | 131 | 40 | 421 | 67 | 127 | 298 | 197 | 277 | 497 | 139 | 67 | 294 | 183 |
| Syntrophobacter | 109 | 143 | 99 | 223 | 39 | 138 | 59 | 288 | 277 | 210 | 233 | 239 | 95 | 71 | 244 | 191 |
| OM27_clade | 80 | 19 | 27 | 30 | 33 | 16 | 20 | 13 | 20 | 46 | 34 | 36 | 23 | 39 | 19 | 57 |
| Subgroup_10 | 47 | 15 | 165 | 60 | 178 | 33 | 147 | 74 | 38 | 72 | 91 | 61 | 213 | 170 | 25 | 53 |
| MND1 | 237 | 40 | 108 | 55 | 431 | 80 | 18 | 37 | 23 | 35 | 38 | 62 | 597 | 97 | 39 | 129 |
| SWB02 | 78 | 62 | 146 | 100 | 752 | 125 | 77 | 66 | 30 | 178 | 139 | 34 | 83 | 154 | 41 | 165 |
| Chthoniobacter | 413 | 32 | 37 | 304 | 126 | 42 | 165 | 222 | 119 | 59 | 8 | 13 | 39 | 176 | 59 | 68 |
| Sulfuricurvum | 368 | 1895 | 53 | 86 | 40 | 86 | 37 | 102 | 74 | 194 | 159 | 1230 | 23 | 11 | 21 | 55 |
| Nitrospira | 133 | 99 | 94 | 257 | 536 | 111 | 75 | 100 | 31 | 70 | 62 | 40 | 266 | 136 | 40 | 167 |
| Sva0081_sediment_group | 256 | 9 | 285 | 313 | 112 | 227 | 163 | 1070 | 504 | 421 | 233 | 161 | 134 | 344 | 226 | 249 |
| Thiobacillus | 69 | 23 | 207 | 57 | 288 | 292 | 66 | 417 | 535 | 80 | 1840 | 1797 | 84 | 233 | 1319 | 299 |
| Desulfobacca | 51 | 4 | 48 | 47 | 15 | 24 | 71 | 104 | 105 | 94 | 77 | 144 | 61 | 55 | 186 | 39 |
| Phaselicystis | 12 | 76 | 45 | 31 | 38 | 43 | 40 | 39 | 40 | 21 | 45 | 35 | 30 | 112 | 31 | 58 |
| ADurb.Bin063-1 | 57 | 218 | 148 | 48 | 97 | 167 | 44 | 150 | 304 | 196 | 267 | 285 | 137 | 85 | 243 | 191 |
| Aquicella | 3 | 6 | 10 | 6 | 31 | 6 | 12 | 4 | 0 | 10 | 7 | 8 | 12 | 12 | 0 | 6 |
| Leptolinea | 26 | 423 | 198 | 127 | 60 | 306 | 112 | 114 | 150 | 274 | 145 | 131 | 55 | 11 | 24 | 71 |
| Thermoanaerobaculum | 37 | 54 | 55 | 53 | 23 | 42 | 55 | 52 | 80 | 46 | 227 | 125 | 35 | 89 | 64 | 48 |
| Hyphomicrobium | 200 | 131 | 190 | 322 | 162 | 253 | 549 | 258 | 176 | 303 | 184 | 96 | 180 | 272 | 157 | 254 |
| Gemmatimonas | 19 | 57 | 56 | 62 | 64 | 142 | 136 | 25 | 31 | 46 | 27 | 20 | 39 | 62 | 12 | 70 |
| Ferruginibacter | 372 | 66 | 54 | 793 | 252 | 133 | 210 | 90 | 140 | 48 | 26 | 12 | 49 | 395 | 84 | 153 |
| Pajaroellobacter | 39 | 3 | 36 | 15 | 59 | 17 | 34 | 18 | 7 | 33 | 33 | 5 | 15 | 117 | 21 | 63 |
| Bacillus | 330 | 69 | 66 | 38 | 58 | 15 | 13 | 58 | 21 | 38 | 161 | 42 | 812 | 21 | 124 | 99 |
| mle1-7 | 360 | 18 | 40 | 162 | 61 | 35 | 62 | 94 | 42 | 38 | 39 | 10 | 214 | 244 | 59 | 83 |
| Candidatus_Endomicrobium | 2 | 28 | 7 | 8 | 3 | 15 | 3 | 2 | 2 | 4 | 2 | 18 | 11 | 3 | 26 | 7 |
| RB41 | 13 | 11 | 5 | 2 | 4 | 17 | 1 | 3 | 9 | 5 | 2 | 10 | 976 | 8 | 2 | 11 |
| Smithella | 5 | 119 | 89 | 78 | 4 | 694 | 33 | 54 | 157 | 56 | 335 | 395 | 15 | 2 | 55 | 23 |
| Crenothrix | 468 | 574 | 393 | 1412 | 835 | 732 | 706 | 1176 | 1950 | 420 | 712 | 523 | 254 | 360 | 443 | 537 |
| Candidatus_Solibacter | 6 | 138 | 97 | 26 | 107 | 73 | 16 | 36 | 7 | 77 | 48 | 46 | 30 | 23 | 35 | 60 |
| Christensenellaceae_R-7_group | 3 | 282 | 92 | 69 | 20 | 174 | 131 | 34 | 37 | 33 | 11 | 49 | 14 | 2 | 12 | 35 |
| RBG-16-58-14 | 62 | 173 | 55 | 17 | 14 | 14 | 40 | 63 | 100 | 64 | 490 | 617 | 124 | 45 | 394 | 122 |
| Ignavibacterium | 243 | 55 | 145 | 300 | 596 | 198 | 114 | 451 | 761 | 201 | 355 | 186 | 337 | 1618 | 437 | 488 |
| Lacunisphaera | 4 | 40 | 6 | 11 | 20 | 13 | 61 | 6 | 5 | 31 | 6 | 14 | 3 | 9 | 14 | 18 |
| Phaeodactylibacter | 28 | 8 | 23 | 53 | 28 | 5 | 31 | 16 | 64 | 26 | 6 | 4 | 5 | 93 | 60 | 125 |
| Sulfurifustis | 273 | 6 | 5 | 262 | 241 | 8 | 20 | 60 | 30 | 47 | 7 | 27 | 236 | 31 | 42 | 82 |
| CL500-29_marine_group | 237 | 6 | 99 | 264 | 211 | 65 | 509 | 43 | 30 | 76 | 38 | 27 | 30 | 67 | 30 | 104 |
| Neochlamydia | 3 | 11 | 3 | 4 | 10 | 0 | 10 | 4 | 13 | 2 | 12 | 6 | 10 | 10 | 7 | 2 |
| Terrimonas | 623 | 243 | 254 | 980 | 796 | 217 | 646 | 155 | 137 | 112 | 39 | 34 | 243 | 548 | 100 | 229 |
| Clostridium_sensu_stricto_1 | 93 | 116 | 54 | 26 | 48 | 42 | 43 | 42 | 33 | 38 | 54 | 396 | 89 | 28 | 267 | 29 |
| Paenibacillus | 11 | 9 | 5 | 3 | 8 | 14 | 4 | 5 | 0 | 8 | 3 | 15 | 55 | 0 | 6 | 1 |
| Desulfomonile | 18 | 17 | 32 | 27 | 6 | 21 | 13 | 32 | 30 | 32 | 43 | 31 | 23 | 9 | 59 | 10 |
| Nocardioides | 10 | 17 | 101 | 36 | 12 | 17 | 16 | 12 | 5 | 59 | 44 | 30 | 181 | 18 | 18 | 79 |
| Peredibacter | 0 | 2 | 6 | 4 | 0 | 6 | 5 | 7 | 1 | 5 | 6 | 36 | 1 | 4 | 9 | 16 |
| Desulfovibrio | 3 | 23 | 13 | 3 | 2 | 33 | 30 | 8 | 1 | 11 | 8 | 4 | 13 | 1 | 1 | 14 |
| Arenimonas | 88 | 52 | 332 | 1346 | 115 | 189 | 1803 | 392 | 246 | 246 | 47 | 140 | 12 | 41 | 62 | 57 |
| Pseudomonas | 92 | 40 | 125 | 87 | 46 | 104 | 124 | 100 | 102 | 117 | 48 | 64 | 46 | 51 | 59 | 69 |
| Pedomicrobium | 125 | 71 | 99 | 43 | 152 | 136 | 62 | 39 | 23 | 119 | 43 | 16 | 154 | 40 | 20 | 102 |
| TPD-58 | 0 | 4 | 24 | 5 | 2 | 26 | 9 | 17 | 22 | 5 | 56 | 53 | 5 | 10 | 23 | 6 |
| BD1-7_clade | 78 | 33 | 82 | 30 | 7 | 129 | 50 | 253 | 246 | 105 | 401 | 139 | 81 | 257 | 196 | 162 |
| Dinghuibacter | 193 | 357 | 61 | 321 | 100 | 183 | 268 | 169 | 180 | 113 | 133 | 70 | 63 | 165 | 306 | 75 |
| Desulfobulbus | 4 | 14 | 83 | 135 | 1 | 117 | 201 | 58 | 85 | 85 | 26 | 67 | 8 | 5 | 7 | 50 |
| Ruminiclostridium_1 | 13 | 77 | 5 | 10 | 9 | 15 | 9 | 12 | 5 | 3 | 11 | 32 | 17 | 0 | 27 | 6 |
| Paludibaculum | 15 | 71 | 131 | 55 | 55 | 92 | 75 | 56 | 39 | 74 | 22 | 22 | 17 | 72 | 36 | 99 |
| Sphingomonas | 88 | 114 | 91 | 12 | 48 | 34 | 44 | 20 | 15 | 26 | 46 | 55 | 49 | 44 | 9 | 84 |
| Geothermobacter | 22 | 230 | 49 | 21 | 1 | 113 | 40 | 102 | 237 | 86 | 70 | 123 | 85 | 85 | 287 | 168 |
| Brevinema | 0 | 11 | 0 | 0 | 2 | 20 | 1 | 4 | 20 | 11 | 12 | 3 | 8 | 3 | 7 | 14 |
| Pirellula | 1 | 0 | 0 | 16 | 7 | 0 | 20 | 6 | 2 | 3 | 1 | 0 | 0 | 4 | 2 | 3 |
| Mycobacterium | 13 | 10 | 77 | 73 | 43 | 40 | 25 | 35 | 12 | 90 | 39 | 17 | 15 | 52 | 7 | 40 |
| Paludibacter | 5 | 41 | 5 | 134 | 8 | 38 | 122 | 2 | 15 | 3 | 4 | 3 | 0 | 11 | 6 | 22 |
| Acidibacter | 36 | 14 | 96 | 103 | 127 | 94 | 63 | 40 | 20 | 94 | 49 | 31 | 72 | 15 | 10 | 112 |
| Novosphingobium | 40 | 165 | 124 | 56 | 53 | 73 | 67 | 27 | 14 | 88 | 29 | 26 | 19 | 118 | 54 | 95 |
| Clostridium_sensu_stricto_12 | 80 | 92 | 16 | 72 | 18 | 28 | 19 | 18 | 11 | 14 | 42 | 183 | 113 | 15 | 189 | 21 |
| AKYG587 | 6 | 6 | 26 | 11 | 69 | 28 | 6 | 22 | 9 | 9 | 5 | 2 | 14 | 22 | 7 | 19 |
| Subgroup_23 | 21 | 8 | 48 | 31 | 11 | 21 | 16 | 47 | 69 | 89 | 68 | 82 | 14 | 57 | 47 | 41 |
| Dongia | 29 | 12 | 115 | 65 | 193 | 62 | 21 | 24 | 7 | 69 | 74 | 220 | 69 | 18 | 8 | 113 |
| OLB12 | 170 | 200 | 119 | 266 | 180 | 352 | 155 | 128 | 220 | 100 | 222 | 94 | 164 | 779 | 475 | 417 |
| Methanosaeta | 24 | 217 | 87 | 241 | 125 | 119 | 50 | 55 | 130 | 136 | 142 | 225 | 25 | 24 | 161 | 73 |
| Candidatus_Udaeobacter | 2 | 29 | 0 | 0 | 2 | 10 | 0 | 3 | 0 | 0 | 0 | 0 | 0 | 0 | 0 | 1 |
| Roseomonas | 19 | 35 | 89 | 129 | 63 | 57 | 308 | 128 | 58 | 119 | 50 | 84 | 17 | 88 | 98 | 85 |
| Allorhizobium-  Neorhizobium-  Pararhizobium-  Rhizobium | 5 | 19 | 68 | 24 | 38 | 21 | 30 | 6 | 3 | 64 | 11 | 32 | 8 | 2 | 2 | 46 |
| Lacihabitans | 5 | 7 | 28 | 78 | 13 | 49 | 167 | 12 | 24 | 21 | 3 | 2 | 1 | 20 | 8 | 45 |
| Sandaracinus | 19 | 0 | 8 | 12 | 11 | 7 | 18 | 3 | 11 | 8 | 5 | 18 | 7 | 14 | 11 | 13 |
| Erysipelothrix | 2 | 98 | 9 | 69 | 6 | 95 | 58 | 10 | 17 | 10 | 14 | 16 | 2 | 5 | 9 | 9 |
| Candidatus_Alysiosphaera | 6 | 2 | 23 | 14 | 20 | 6 | 14 | 7 | 6 | 13 | 13 | 2 | 3 | 2 | 1 | 6 |
| Gaiella | 153 | 18 | 256 | 59 | 134 | 58 | 75 | 79 | 21 | 65 | 61 | 21 | 158 | 295 | 11 | 119 |
| Longilinea | 40 | 389 | 156 | 121 | 31 | 166 | 100 | 103 | 128 | 244 | 126 | 230 | 27 | 23 | 143 | 118 |
| WCHB1-32 | 3 | 49 | 9 | 22 | 3 | 23 | 40 | 0 | 4 | 1 | 2 | 8 | 0 | 1 | 0 | 4 |
| Treponema | 2 | 29 | 4 | 7 | 8 | 11 | 6 | 5 | 4 | 6 | 3 | 14 | 17 | 4 | 5 | 11 |
| Hydrogenispora | 20 | 30 | 3 | 6 | 5 | 13 | 7 | 5 | 2 | 2 | 10 | 31 | 34 | 9 | 20 | 18 |
| Defluviicoccus | 12 | 4 | 27 | 5 | 17 | 4 | 8 | 25 | 9 | 12 | 151 | 8 | 27 | 7 | 3 | 25 |
| Deferrisoma | 18 | 41 | 1 | 4 | 0 | 2 | 2 | 11 | 12 | 2 | 3 | 4 | 24 | 185 | 35 | 8 |
| Cyanobium_PCC-6307 | 21 | 0 | 3 | 7 | 15 | 8 | 20 | 12 | 20 | 9 | 3 | 0 | 2 | 8 | 6 | 12 |
| OM60NOR5_clade | 215 | 35 | 337 | 1312 | 87 | 419 | 859 | 865 | 1064 | 571 | 196 | 137 | 84 | 364 | 386 | 525 |
| Nannocystis | 16 | 5 | 19 | 3 | 28 | 5 | 2 | 3 | 13 | 6 | 11 | 5 | 3 | 8 | 8 | 10 |
| Streptomyces | 4 | 4 | 34 | 2 | 29 | 17 | 1 | 0 | 1 | 26 | 4 | 13 | 13 | 2 | 1 | 8 |
| Sh765B-TzT-35 | 16 | 15 | 9 | 13 | 6 | 12 | 3 | 15 | 18 | 33 | 35 | 49 | 40 | 12 | 52 | 30 |
| BSV13 | 0 | 97 | 30 | 15 | 11 | 37 | 5 | 26 | 19 | 13 | 19 | 20 | 6 | 1 | 1 | 5 |
| Desulfosporosinus | 64 | 39 | 62 | 33 | 10 | 91 | 53 | 33 | 63 | 38 | 32 | 82 | 23 | 48 | 80 | 31 |
| Lysobacter | 3 | 17 | 137 | 20 | 4 | 21 | 16 | 10 | 3 | 25 | 4 | 44 | 28 | 14 | 12 | 32 |
| Lautropia | 48 | 4 | 34 | 47 | 8 | 30 | 46 | 82 | 58 | 28 | 96 | 20 | 14 | 126 | 45 | 58 |
| M2PT2-76_termite_group | 1 | 5 | 3 | 2 | 0 | 6 | 5 | 2 | 3 | 1 | 4 | 12 | 8 | 0 | 8 | 10 |
| Methylotenera | 11 | 59 | 85 | 156 | 52 | 168 | 250 | 49 | 35 | 82 | 362 | 40 | 65 | 23 | 38 | 48 |
| Reyranella | 5 | 9 | 76 | 17 | 24 | 37 | 15 | 11 | 2 | 37 | 7 | 10 | 10 | 7 | 2 | 28 |
| Ilumatobacter | 580 | 47 | 263 | 516 | 201 | 154 | 670 | 267 | 224 | 324 | 88 | 44 | 90 | 363 | 116 | 383 |
| Conexibacter | 23 | 2 | 66 | 14 | 16 | 12 | 6 | 15 | 18 | 30 | 14 | 1 | 5 | 39 | 1 | 16 |
| Geothrix | 1 | 31 | 13 | 15 | 2 | 73 | 5 | 11 | 39 | 32 | 0 | 7 | 3 | 11 | 9 | 24 |
| Iamia | 80 | 6 | 142 | 15 | 35 | 13 | 19 | 13 | 7 | 34 | 5 | 5 | 22 | 49 | 13 | 67 |
| Altererythrobacter | 0 | 4 | 35 | 6 | 28 | 15 | 8 | 4 | 6 | 8 | 3 | 2 | 4 | 4 | 8 | 16 |
| Blvii28_wastewater-sludge_group | 2 | 15 | 4 | 8 | 1 | 30 | 6 | 6 | 24 | 5 | 1 | 11 | 3 | 4 | 3 | 27 |
| Acidaminobacter | 70 | 66 | 30 | 78 | 3 | 26 | 19 | 36 | 21 | 22 | 10 | 53 | 44 | 15 | 47 | 54 |
| Bauldia | 53 | 9 | 46 | 10 | 151 | 31 | 10 | 7 | 4 | 13 | 5 | 5 | 50 | 9 | 3 | 18 |
| Pelolinea | 30 | 164 | 27 | 33 | 9 | 24 | 11 | 33 | 34 | 25 | 68 | 86 | 32 | 18 | 40 | 40 |
| Anaerovorax | 9 | 58 | 39 | 72 | 3 | 82 | 17 | 27 | 8 | 15 | 23 | 37 | 10 | 0 | 14 | 7 |
| Devosia | 9 | 59 | 90 | 30 | 171 | 91 | 28 | 11 | 4 | 76 | 26 | 20 | 20 | 39 | 4 | 59 |
| Methyloparacoccus | 11 | 0 | 74 | 29 | 6 | 92 | 130 | 61 | 112 | 20 | 129 | 18 | 4 | 33 | 9 | 23 |
| CL500-3 | 0 | 0 | 1 | 13 | 3 | 11 | 3 | 0 | 2 | 5 | 3 | 0 | 0 | 7 | 0 | 0 |
| Oligoflexus | 1 | 1 | 37 | 0 | 2 | 0 | 6 | 0 | 4 | 3 | 1 | 1 | 0 | 3 | 4 | 4 |
| Halomonas | 0 | 0 | 3 | 12 | 0 | 15 | 20 | 11 | 3 | 31 | 6 | 16 | 2 | 15 | 31 | 33 |
| Singulisphaera | 5 | 0 | 4 | 7 | 3 | 0 | 3 | 11 | 1 | 10 | 1 | 0 | 5 | 1 | 0 | 2 |
| Tumebacillus | 0 | 4 | 19 | 20 | 13 | 0 | 0 | 6 | 8 | 8 | 7 | 11 | 4 | 1 | 7 | 6 |
| Flavisolibacter | 0 | 22 | 3 | 0 | 0 | 2 | 1 | 2 | 3 | 0 | 0 | 5 | 2 | 1 | 0 | 4 |
| Massilia | 13 | 3 | 2 | 4 | 0 | 10 | 1 | 5 | 0 | 14 | 7 | 140 | 12 | 8 | 3 | 21 |
| PAUC26f | 2 | 4 | 16 | 18 | 55 | 22 | 6 | 1 | 1 | 6 | 3 | 5 | 19 | 1 | 4 | 5 |
| Rhodococcus | 0 | 1 | 10 | 2 | 2 | 1 | 5 | 1 | 0 | 5 | 4 | 4 | 0 | 1 | 0 | 1 |
| Phenylobacterium | 4 | 22 | 35 | 12 | 27 | 27 | 40 | 12 | 7 | 46 | 21 | 113 | 18 | 9 | 13 | 31 |
| Steroidobacter | 9 | 13 | 15 | 2 | 8 | 8 | 1 | 2 | 2 | 5 | 5 | 3 | 158 | 6 | 0 | 80 |
| Legionella | 0 | 0 | 9 | 1 | 0 | 2 | 4 | 1 | 3 | 0 | 0 | 0 | 3 | 3 | 0 | 2 |
| Hydrogenophaga | 31 | 62 | 382 | 431 | 63 | 137 | 391 | 42 | 34 | 126 | 49 | 59 | 7 | 16 | 33 | 109 |
| Fluviicola | 1 | 7 | 7 | 3 | 5 | 0 | 10 | 0 | 7 | 0 | 1 | 0 | 1 | 8 | 13 | 0 |
| Candidatus_Latescibacter | 2 | 2 | 0 | 0 | 1 | 2 | 0 | 2 | 0 | 1 | 4 | 13 | 2 | 0 | 10 | 0 |
| Ohtaekwangia | 0 | 1 | 1 | 2 | 4 | 1 | 21 | 0 | 0 | 7 | 8 | 33 | 0 | 5 | 0 | 5 |
| Candidatus_Ovatusbacter | 0 | 0 | 7 | 4 | 0 | 0 | 0 | 0 | 2 | 3 | 0 | 0 | 0 | 2 | 0 | 0 |
| Emticicia | 4 | 1 | 1 | 24 | 12 | 2 | 68 | 5 | 0 | 6 | 2 | 0 | 0 | 0 | 3 | 0 |
| Lutispora | 3 | 5 | 0 | 0 | 1 | 5 | 3 | 2 | 0 | 0 | 0 | 0 | 5 | 0 | 4 | 0 |
| Bacteroides | 1 | 3 | 2 | 100 | 0 | 0 | 2 | 7 | 0 | 2 | 3 | 7 | 3 | 2 | 11 | 15 |
| Nitrosomonas | 0 | 2 | 2 | 9 | 0 | 0 | 0 | 10 | 5 | 4 | 2 | 0 | 3 | 3 | 0 | 0 |
| Sulfuritalea | 10 | 14 | 9 | 11 | 4 | 84 | 29 | 32 | 61 | 30 | 13 | 37 | 20 | 17 | 31 | 77 |
| Thioalkalispira | 2 | 2 | 4 | 1 | 0 | 1 | 3 | 1 | 2 | 2 | 12 | 520 | 0 | 0 | 381 | 1 |
| Meiothermus | 110 | 13 | 0 | 318 | 24 | 8 | 108 | 19 | 15 | 19 | 2 | 0 | 23 | 9 | 15 | 16 |
| Woeseia | 39 | 9 | 33 | 485 | 30 | 78 | 134 | 136 | 194 | 160 | 33 | 13 | 12 | 58 | 45 | 37 |
| Microvirga | 10 | 19 | 48 | 9 | 8 | 9 | 5 | 16 | 4 | 41 | 16 | 10 | 12 | 6 | 3 | 61 |
| Caldisericum | 3 | 3 | 10 | 112 | 2 | 28 | 3 | 7 | 9 | 1 | 12 | 40 | 5 | 0 | 0 | 0 |
| Hirschia | 29 | 16 | 196 | 33 | 233 | 88 | 51 | 21 | 22 | 110 | 41 | 28 | 4 | 29 | 12 | 51 |
| Methanobacterium | 0 | 5 | 1 | 3 | 7 | 0 | 2 | 2 | 2 | 8 | 0 | 2 | 0 | 2 | 6 | 1 |
| Uliginosibacterium | 0 | 6 | 3 | 21 | 0 | 1 | 40 | 1 | 0 | 1 | 0 | 4 | 4 | 0 | 10 | 34 |
| Candidatus_Paracaedibacter | 5 | 0 | 2 | 0 | 10 | 7 | 2 | 5 | 1 | 1 | 0 | 1 | 2 | 5 | 8 | 0 |
| Roseimarinus | 0 | 1 | 9 | 5 | 4 | 18 | 9 | 3 | 1 | 5 | 2 | 8 | 1 | 0 | 0 | 8 |
| Rhodobacter | 23 | 115 | 262 | 519 | 107 | 214 | 682 | 98 | 85 | 254 | 33 | 22 | 16 | 55 | 24 | 252 |
| SCGC-AB-539-J10 | 7 | 2 | 0 | 0 | 0 | 0 | 0 | 0 | 2 | 0 | 6 | 115 | 0 | 0 | 3 | 4 |
| Caproiciproducens | 0 | 73 | 1 | 0 | 1 | 0 | 0 | 0 | 0 | 4 | 2 | 1 | 0 | 4 | 4 | 0 |
| Syntrophomonas | 5 | 29 | 16 | 20 | 1 | 30 | 6 | 4 | 0 | 1 | 1 | 62 | 5 | 5 | 39 | 12 |
| Chloronema | 0 | 2 | 21 | 0 | 1 | 0 | 5 | 0 | 2 | 0 | 1 | 0 | 0 | 0 | 0 | 0 |
| Desulfomicrobium | 0 | 19 | 43 | 13 | 3 | 41 | 83 | 6 | 11 | 7 | 26 | 18 | 2 | 2 | 6 | 10 |
| JdFR-76 | 0 | 0 | 2 | 0 | 10 | 0 | 0 | 1 | 0 | 0 | 0 | 0 | 28 | 7 | 0 | 0 |
| Methanoregula | 7 | 55 | 14 | 45 | 3 | 21 | 8 | 27 | 41 | 17 | 42 | 59 | 7 | 12 | 64 | 22 |
| IS-44 | 20 | 3 | 11 | 11 | 17 | 21 | 3 | 4 | 2 | 16 | 1 | 7 | 185 | 0 | 0 | 9 |
| Rhodomicrobium | 23 | 19 | 17 | 11 | 20 | 4 | 0 | 7 | 1 | 20 | 10 | 2 | 2 | 0 | 6 | 24 |
| Alterococcus | 0 | 7 | 7 | 0 | 14 | 9 | 1 | 0 | 1 | 8 | 1 | 10 | 0 | 0 | 3 | 13 |
| Blastopirellula | 8 | 0 | 0 | 2 | 0 | 0 | 2 | 0 | 0 | 0 | 0 | 0 | 0 | 0 | 3 | 0 |
| Desulfonema | 0 | 0 | 3 | 0 | 0 | 2 | 4 | 1 | 0 | 2 | 0 | 0 | 14 | 0 | 0 | 3 |
| Blastocatella | 104 | 6 | 1 | 38 | 5 | 5 | 76 | 2 | 3 | 6 | 1 | 1 | 7 | 3 | 4 | 10 |
| Sideroxydans | 13 | 52 | 29 | 14 | 33 | 16 | 14 | 18 | 2 | 9 | 52 | 312 | 68 | 2 | 41 | 35 |
| Cytophaga | 0 | 0 | 0 | 1 | 0 | 0 | 3 | 0 | 0 | 2 | 0 | 0 | 0 | 5 | 0 | 4 |
| Solirubrobacter | 1 | 1 | 12 | 2 | 0 | 8 | 0 | 7 | 1 | 15 | 4 | 0 | 4 | 0 | 0 | 32 |
| Fusibacter | 18 | 3 | 4 | 18 | 0 | 23 | 14 | 7 | 0 | 1 | 2 | 6 | 1 | 1 | 2 | 11 |
| Turneriella | 1 | 2 | 1 | 0 | 0 | 2 | 5 | 0 | 0 | 1 | 0 | 1 | 18 | 11 | 0 | 2 |
| Candidatus_Accumulibacter | 5 | 3 | 47 | 99 | 15 | 55 | 114 | 84 | 179 | 30 | 76 | 18 | 10 | 21 | 21 | 54 |
| Candidatus_Omnitrophus | 0 | 0 | 0 | 0 | 0 | 1 | 0 | 0 | 2 | 1 | 0 | 0 | 6 | 0 | 1 | 2 |
| IMCC26207 | 4 | 0 | 21 | 30 | 16 | 12 | 36 | 14 | 17 | 26 | 5 | 3 | 5 | 30 | 7 | 30 |
| Amphiplicatus | 0 | 1 | 19 | 0 | 13 | 5 | 2 | 1 | 0 | 0 | 0 | 0 | 3 | 6 | 0 | 4 |
| Clostridium_sensu_stricto_13 | 18 | 19 | 10 | 61 | 6 | 48 | 6 | 18 | 13 | 7 | 21 | 91 | 20 | 13 | 88 | 20 |
| Candidatus_Protochlamydia | 0 | 0 | 3 | 1 | 5 | 2 | 2 | 1 | 0 | 0 | 3 | 0 | 3 | 1 | 4 | 0 |
| SM23-31 | 1 | 11 | 0 | 10 | 0 | 0 | 0 | 1 | 6 | 3 | 4 | 21 | 1 | 2 | 1 | 4 |
| Stenotrophobacter | 9 | 2 | 7 | 21 | 19 | 22 | 27 | 1 | 9 | 11 | 2 | 0 | 7 | 84 | 11 | 11 |
| hgcI_clade | 3 | 2 | 0 | 2 | 0 | 0 | 1 | 10 | 2 | 0 | 0 | 3 | 2 | 0 | 0 | 0 |
| Levilinea | 1 | 32 | 14 | 5 | 0 | 6 | 12 | 9 | 0 | 1 | 0 | 26 | 12 | 0 | 12 | 5 |
| Candidatus_Captivus | 0 | 3 | 2 | 1 | 0 | 0 | 3 | 4 | 0 | 0 | 1 | 0 | 0 | 2 | 0 | 0 |
| Roseimicrobium | 0 | 3 | 11 | 0 | 3 | 0 | 0 | 0 | 0 | 2 | 1 | 0 | 0 | 2 | 0 | 4 |
| Runella | 0 | 0 | 0 | 0 | 2 | 2 | 41 | 2 | 0 | 5 | 3 | 1 | 0 | 0 | 0 | 0 |
| 966-1 | 27 | 0 | 5 | 7 | 14 | 1 | 1 | 14 | 7 | 7 | 6 | 0 | 2 | 60 | 4 | 27 |
| Ruminococcaceae_UCG-014 | 0 | 11 | 4 | 0 | 0 | 6 | 3 | 0 | 0 | 0 | 0 | 9 | 1 | 0 | 0 | 0 |
| oc32 | 10 | 2 | 2 | 1 | 0 | 3 | 5 | 15 | 11 | 0 | 0 | 3 | 62 | 114 | 60 | 2 |
| Planktothricoides_SR001 | 0 | 0 | 1 | 0 | 0 | 2 | 2 | 5 | 112 | 0 | 4 | 0 | 0 | 0 | 0 | 0 |
| Rhodoferax | 10 | 59 | 412 | 45 | 25 | 384 | 98 | 38 | 56 | 48 | 85 | 38 | 19 | 15 | 4 | 25 |
| Dokdonella | 0 | 1 | 1 | 2 | 10 | 7 | 0 | 0 | 0 | 10 | 0 | 0 | 0 | 0 | 0 | 0 |
| Planktothrix_NIVA-CYA_15 | 0 | 0 | 59 | 0 | 1 | 0 | 1 | 27 | 18 | 0 | 0 | 0 | 0 | 0 | 1 | 2 |
| Brevundimonas | 7 | 4 | 14 | 1 | 8 | 6 | 33 | 6 | 4 | 10 | 11 | 5 | 0 | 10 | 15 | 6 |
| Methyloglobulus | 0 | 0 | 7 | 3 | 0 | 13 | 0 | 9 | 0 | 7 | 1 | 9 | 0 | 1 | 0 | 2 |
| Desulfurivibrio | 0 | 4 | 0 | 2 | 0 | 4 | 2 | 1 | 3 | 1 | 2 | 10 | 9 | 4 | 3 | 7 |
| Desulfovirga | 2 | 7 | 5 | 7 | 2 | 9 | 9 | 11 | 14 | 13 | 28 | 50 | 7 | 0 | 9 | 21 |
| Sporomusa | 0 | 1 | 2 | 0 | 0 | 0 | 0 | 1 | 0 | 0 | 1 | 2 | 15 | 0 | 8 | 0 |
| Sporocytophaga | 0 | 0 | 0 | 0 | 1 | 0 | 4 | 0 | 0 | 1 | 0 | 2 | 2 | 0 | 1 | 0 |
| Ferrovibrio | 0 | 0 | 1 | 0 | 10 | 1 | 2 | 0 | 0 | 13 | 0 | 6 | 0 | 7 | 0 | 3 |
| Pir4_lineage | 0 | 1 | 3 | 2 | 0 | 0 | 0 | 3 | 0 | 0 | 0 | 0 | 0 | 0 | 0 | 2 |
| Phreatobacter | 3 | 11 | 34 | 5 | 3 | 7 | 33 | 3 | 4 | 23 | 3 | 7 | 2 | 1 | 0 | 9 |
| Acinetobacter | 18 | 0 | 4 | 1 | 0 | 2 | 0 | 2 | 0 | 0 | 0 | 0 | 0 | 0 | 0 | 0 |
| Methanospirillum | 0 | 17 | 1 | 3 | 0 | 4 | 0 | 1 | 0 | 0 | 0 | 4 | 0 | 0 | 1 | 0 |
| Epulopiscium | 1 | 0 | 3 | 12 | 0 | 3 | 3 | 1 | 1 | 1 | 1 | 7 | 0 | 0 | 1 | 0 |
| Bacteriovorax | 0 | 0 | 8 | 1 | 2 | 3 | 9 | 0 | 0 | 0 | 5 | 57 | 0 | 4 | 12 | 0 |
| AAP99 | 7 | 2 | 13 | 7 | 4 | 9 | 11 | 4 | 1 | 7 | 0 | 1 | 3 | 8 | 6 | 9 |
| Calorithrix | 18 | 10 | 0 | 4 | 0 | 4 | 1 | 4 | 34 | 2 | 23 | 21 | 0 | 22 | 155 | 17 |
| Anaerolineaceae_UCG-001 | 0 | 2 | 0 | 10 | 1 | 6 | 3 | 8 | 0 | 2 | 1 | 2 | 0 | 0 | 0 | 0 |
| UKL13-1 | 2 | 0 | 8 | 3 | 0 | 0 | 8 | 1 | 0 | 4 | 0 | 1 | 0 | 2 | 0 | 2 |
| Adhaeribacter | 0 | 9 | 10 | 2 | 0 | 11 | 1 | 0 | 0 | 3 | 4 | 0 | 2 | 0 | 2 | 8 |
| Pseudolabrys | 39 | 30 | 162 | 50 | 84 | 81 | 31 | 29 | 4 | 108 | 31 | 28 | 48 | 9 | 18 | 66 |
| Acetobacteroides | 0 | 18 | 4 | 33 | 5 | 11 | 2 | 4 | 2 | 2 | 2 | 9 | 0 | 2 | 12 | 9 |
| Aeromonas | 7 | 16 | 3 | 27 | 1 | 24 | 32 | 0 | 1 | 7 | 1 | 3 | 0 | 4 | 11 | 0 |
| HSB_OF53-F07 | 0 | 2 | 0 | 0 | 0 | 4 | 3 | 0 | 0 | 0 | 0 | 0 | 0 | 0 | 0 | 0 |
| Cetobacterium | 1 | 3 | 20 | 11 | 0 | 2 | 12 | 33 | 4 | 9 | 1 | 4 | 0 | 0 | 2 | 4 |
| Salinispira | 5 | 0 | 2 | 1 | 2 | 4 | 8 | 1 | 4 | 0 | 0 | 1 | 9 | 0 | 2 | 12 |
| Propionivibrio | 1 | 14 | 4 | 2 | 1 | 8 | 100 | 5 | 6 | 5 | 1 | 12 | 15 | 1 | 10 | 9 |
| Sva0996_marine_group | 75 | 0 | 0 | 16 | 0 | 0 | 7 | 0 | 1 | 3 | 2 | 0 | 0 | 5 | 0 | 8 |
| JGI_0001001-H03 | 254 | 28 | 13 | 150 | 103 | 21 | 118 | 46 | 35 | 19 | 16 | 9 | 45 | 110 | 25 | 20 |
| Curvibacter | 0 | 8 | 0 | 1 | 0 | 1 | 2 | 0 | 0 | 0 | 2 | 71 | 0 | 0 | 5 | 0 |
| Opitutus | 4 | 19 | 4 | 28 | 11 | 14 | 17 | 13 | 31 | 36 | 1 | 5 | 5 | 16 | 16 | 29 |
| Armatimonas | 0 | 1 | 0 | 1 | 0 | 4 | 5 | 0 | 2 | 12 | 2 | 0 | 0 | 4 | 1 | 2 |
| Lacibacter | 175 | 124 | 20 | 372 | 242 | 15 | 200 | 16 | 18 | 21 | 9 | 3 | 51 | 178 | 26 | 14 |
| GOUTB8 | 29 | 70 | 28 | 10 | 33 | 34 | 12 | 19 | 14 | 23 | 53 | 65 | 41 | 23 | 56 | 22 |
| Leptospirillum | 21 | 0 | 0 | 3 | 3 | 2 | 1 | 0 | 0 | 2 | 0 | 0 | 141 | 0 | 2 | 1 |
| Parasegetibacter | 0 | 6 | 9 | 1 | 0 | 2 | 0 | 0 | 0 | 0 | 0 | 0 | 0 | 0 | 0 | 1 |
| Fonticella | 1 | 1 | 0 | 3 | 9 | 3 | 3 | 0 | 0 | 3 | 9 | 0 | 14 | 1 | 23 | 8 |
| GOUTA6 | 9 | 13 | 2 | 7 | 0 | 3 | 13 | 3 | 2 | 3 | 2 | 6 | 101 | 26 | 11 | 20 |
| Azoarcus | 0 | 27 | 5 | 0 | 0 | 0 | 3 | 0 | 0 | 0 | 1 | 0 | 0 | 1 | 2 | 0 |
| Gemmatirosa | 0 | 0 | 3 | 0 | 2 | 2 | 0 | 0 | 0 | 0 | 0 | 0 | 1 | 0 | 0 | 6 |
| Silanimonas | 0 | 0 | 53 | 1 | 8 | 39 | 81 | 1 | 1 | 4 | 0 | 0 | 0 | 1 | 0 | 3 |
| Kaistia | 3 | 0 | 6 | 4 | 2 | 13 | 1 | 0 | 3 | 3 | 2 | 0 | 0 | 2 | 0 | 0 |
| Sphingobium | 0 | 2 | 8 | 1 | 0 | 4 | 0 | 3 | 0 | 15 | 0 | 0 | 0 | 2 | 2 | 2 |
| MSBL7 | 0 | 0 | 1 | 0 | 0 | 0 | 0 | 0 | 4 | 1 | 3 | 16 | 2 | 0 | 1 | 0 |
| Sulfurovum | 1 | 14 | 3 | 5 | 1 | 3 | 0 | 4 | 1 | 2 | 5 | 15 | 0 | 0 | 1 | 0 |
| Limnobacter | 0 | 0 | 20 | 0 | 0 | 0 | 0 | 0 | 0 | 3 | 1 | 7 | 0 | 0 | 4 | 0 |
| Pir2_lineage | 0 | 2 | 4 | 0 | 0 | 0 | 0 | 0 | 0 | 0 | 0 | 0 | 0 | 0 | 0 | 0 |
| Skermanella | 5 | 3 | 14 | 4 | 2 | 3 | 2 | 6 | 1 | 10 | 14 | 3 | 0 | 0 | 0 | 51 |
| GWE2-42-42 | 0 | 0 | 2 | 1 | 4 | 4 | 2 | 0 | 1 | 0 | 3 | 0 | 0 | 0 | 0 | 0 |
| GWD2-49-16 | 2 | 49 | 0 | 0 | 0 | 1 | 1 | 0 | 2 | 0 | 3 | 8 | 0 | 0 | 0 | 1 |
| Hypnocyclicus | 42 | 7 | 0 | 0 | 1 | 2 | 1 | 2 | 0 | 0 | 0 | 8 | 24 | 1 | 33 | 2 |
| Ruminiclostridium | 1 | 8 | 1 | 25 | 3 | 10 | 34 | 10 | 11 | 7 | 4 | 10 | 0 | 0 | 3 | 26 |
| Halioglobus | 154 | 22 | 134 | 510 | 35 | 220 | 402 | 391 | 411 | 247 | 77 | 39 | 55 | 385 | 266 | 223 |
| Haliscomenobacter | 2 | 1 | 1 | 4 | 0 | 2 | 8 | 1 | 9 | 11 | 0 | 2 | 2 | 21 | 13 | 3 |
| Oscillatoria_PCC-10802 | 0 | 0 | 13 | 0 | 0 | 0 | 0 | 0 | 1 | 0 | 0 | 154 | 1 | 2 | 0 | 0 |
| Candidatus_Berkiella | 0 | 0 | 0 | 0 | 3 | 2 | 0 | 2 | 0 | 1 | 0 | 2 | 2 | 3 | 0 | 0 |
| Defluviitaleaceae_UCG-011 | 2 | 0 | 0 | 8 | 2 | 0 | 32 | 0 | 0 | 2 | 0 | 0 | 0 | 1 | 1 | 3 |
| SH-PL14 | 0 | 3 | 0 | 0 | 0 | 0 | 1 | 0 | 0 | 0 | 0 | 0 | 0 | 4 | 0 | 1 |
| alphaI_cluster | 23 | 13 | 70 | 26 | 24 | 31 | 111 | 38 | 34 | 54 | 52 | 20 | 6 | 27 | 14 | 23 |
| Candidatus_  Xiphinematobacter | 0 | 9 | 6 | 19 | 6 | 12 | 0 | 12 | 7 | 4 | 4 | 3 | 3 | 1 | 0 | 42 |
| Candidatus_Methylospira | 1 | 0 | 0 | 1 | 0 | 2 | 2 | 6 | 5 | 3 | 10 | 1 | 1 | 16 | 1 | 0 |
| C1-B045 | 1 | 0 | 4 | 0 | 0 | 0 | 0 | 0 | 2 | 0 | 0 | 21 | 0 | 0 | 0 | 0 |
| Methylomonas | 1 | 17 | 4 | 8 | 3 | 29 | 1 | 6 | 23 | 21 | 3 | 14 | 9 | 5 | 7 | 19 |
| Oscillochloris | 0 | 0 | 13 | 0 | 0 | 0 | 0 | 0 | 0 | 0 | 0 | 0 | 0 | 0 | 0 | 0 |
| Denitratisoma | 3 | 5 | 1 | 2 | 0 | 4 | 5 | 2 | 1 | 27 | 27 | 2 | 0 | 1 | 0 | 6 |
| Oxobacter | 0 | 1 | 2 | 1 | 0 | 0 | 0 | 1 | 0 | 0 | 0 | 0 | 4 | 1 | 0 | 0 |
| Cellvibrio | 0 | 5 | 0 | 7 | 1 | 1 | 17 | 0 | 0 | 3 | 0 | 0 | 0 | 0 | 0 | 3 |
| Pseudonocardia | 0 | 5 | 6 | 3 | 4 | 3 | 0 | 5 | 1 | 8 | 0 | 1 | 2 | 1 | 0 | 34 |
| Z114MB74 | 19 | 0 | 2 | 4 | 0 | 0 | 0 | 2 | 4 | 2 | 4 | 13 | 0 | 0 | 35 | 9 |
| Candidatus_Amoebophilus | 63 | 0 | 4 | 2 | 8 | 2 | 3 | 0 | 9 | 1 | 2 | 0 | 1 | 0 | 0 | 0 |
| Chlorobaculum | 0 | 0 | 68 | 0 | 4 | 0 | 0 | 0 | 4 | 0 | 26 | 4 | 0 | 0 | 0 | 0 |
| Chthonomonas | 4 | 0 | 3 | 0 | 0 | 0 | 2 | 0 | 0 | 0 | 0 | 0 | 0 | 0 | 0 | 2 |
| Niastella | 1 | 1 | 2 | 0 | 3 | 0 | 0 | 0 | 0 | 1 | 2 | 0 | 0 | 0 | 0 | 5 |
| Vulgatibacter | 0 | 0 | 0 | 0 | 0 | 0 | 0 | 0 | 0 | 0 | 0 | 4 | 0 | 2 | 0 | 0 |
| Agromyces | 1 | 0 | 18 | 3 | 0 | 2 | 0 | 1 | 2 | 2 | 10 | 3 | 1 | 0 | 2 | 31 |
| Clostridium_sensu_stricto_3 | 162 | 100 | 73 | 96 | 102 | 106 | 96 | 104 | 99 | 101 | 68 | 163 | 63 | 89 | 157 | 96 |
| Chitinophaga | 0 | 0 | 0 | 0 | 6 | 0 | 5 | 0 | 0 | 0 | 0 | 0 | 0 | 1 | 0 | 0 |
| Clostridium_sensu_stricto_8 | 6 | 7 | 4 | 2 | 2 | 4 | 2 | 4 | 1 | 2 | 3 | 11 | 35 | 2 | 31 | 1 |
| Desulforhabdus | 0 | 3 | 57 | 3 | 3 | 11 | 5 | 6 | 8 | 3 | 5 | 14 | 1 | 0 | 1 | 0 |
| Pseudoxanthomonas | 0 | 2 | 5 | 6 | 10 | 16 | 2 | 0 | 0 | 1 | 3 | 1 | 0 | 1 | 0 | 2 |
| Ferritrophicum | 2 | 2 | 15 | 8 | 39 | 6 | 14 | 21 | 6 | 13 | 123 | 203 | 7 | 5 | 0 | 12 |
| Polyangium | 0 | 4 | 4 | 4 | 2 | 3 | 2 | 2 | 0 | 9 | 7 | 5 | 2 | 2 | 0 | 23 |
| Dysgonomonas | 0 | 2 | 0 | 0 | 2 | 0 | 5 | 0 | 0 | 0 | 0 | 0 | 0 | 2 | 0 | 0 |
| Sediminibacterium | 9 | 9 | 7 | 11 | 5 | 28 | 54 | 5 | 13 | 9 | 0 | 0 | 8 | 0 | 24 | 24 |
| Thermincola | 1 | 1 | 0 | 1 | 0 | 2 | 0 | 0 | 2 | 2 | 0 | 0 | 56 | 4 | 1 | 0 |
| Segetibacter | 2 | 2 | 3 | 62 | 18 | 0 | 8 | 2 | 3 | 6 | 0 | 1 | 0 | 2 | 4 | 18 |
| Candidatus_Electronema | 0 | 0 | 24 | 0 | 0 | 3 | 0 | 0 | 0 | 1 | 0 | 0 | 1 | 0 | 0 | 0 |
| Pleurocapsa_PCC-7319 | 3 | 0 | 1 | 77 | 0 | 0 | 2 | 0 | 0 | 0 | 0 | 1 | 1 | 0 | 0 | 0 |
| Thauera | 0 | 15 | 1 | 8 | 0 | 9 | 1 | 2 | 0 | 5 | 3 | 5 | 0 | 0 | 0 | 0 |
| Flavihumibacter | 1 | 75 | 12 | 10 | 10 | 3 | 7 | 9 | 2 | 2 | 10 | 24 | 2 | 0 | 2 | 3 |
| Methylobacterium | 0 | 1 | 50 | 0 | 0 | 0 | 1 | 0 | 0 | 0 | 4 | 7 | 2 | 0 | 1 | 4 |
| Thiothrix | 0 | 0 | 2 | 2 | 5 | 0 | 1 | 1 | 0 | 0 | 0 | 0 | 0 | 0 | 0 | 0 |
| Nordella | 3 | 10 | 2 | 1 | 0 | 6 | 1 | 2 | 0 | 0 | 1 | 1 | 35 | 0 | 0 | 0 |
| Sedimentibacter | 1 | 1 | 2 | 9 | 0 | 0 | 0 | 0 | 0 | 0 | 1 | 9 | 2 | 0 | 4 | 2 |
| Candidatus_Koribacter | 0 | 4 | 4 | 0 | 0 | 2 | 0 | 1 | 0 | 0 | 0 | 0 | 18 | 0 | 0 | 3 |
| Desulfuromonas | 4 | 86 | 9 | 1 | 1 | 21 | 1 | 0 | 1 | 1 | 0 | 5 | 2 | 0 | 0 | 1 |
| Methylomicrobium | 10 | 9 | 1 | 26 | 3 | 2 | 18 | 1 | 5 | 12 | 5 | 5 | 19 | 3 | 36 | 8 |
| OLB13 | 8 | 0 | 3 | 16 | 13 | 1 | 2 | 1 | 2 | 4 | 0 | 0 | 1 | 3 | 0 | 8 |
| Litorilinea | 0 | 0 | 0 | 0 | 1 | 4 | 0 | 0 | 0 | 0 | 0 | 0 | 1 | 0 | 0 | 3 |
| Parviterribacter | 0 | 0 | 4 | 0 | 3 | 0 | 0 | 0 | 0 | 0 | 0 | 0 | 2 | 0 | 0 | 0 |
| Nodosilinea_PCC-7104 | 1 | 1 | 0 | 0 | 2 | 1 | 7 | 0 | 8 | 4 | 0 | 0 | 0 | 1 | 1 | 1 |
| Parvibaculum | 0 | 2 | 3 | 0 | 0 | 0 | 0 | 0 | 0 | 0 | 0 | 4 | 0 | 0 | 0 | 0 |
| Noviherbaspirillum | 0 | 8 | 7 | 2 | 4 | 7 | 2 | 1 | 0 | 17 | 0 | 3 | 2 | 1 | 0 | 2 |
| Leptolyngbya_ANT.L52.2 | 0 | 0 | 0 | 0 | 0 | 0 | 0 | 0 | 4 | 0 | 0 | 0 | 0 | 0 | 0 | 3 |
| Asticcacaulis | 0 | 0 | 21 | 0 | 0 | 4 | 0 | 0 | 0 | 0 | 1 | 0 | 1 | 0 | 2 | 3 |
| Ruminococcus_1 | 1 | 6 | 2 | 1 | 0 | 0 | 24 | 0 | 0 | 1 | 0 | 0 | 0 | 2 | 6 | 4 |
| Methylococcus | 0 | 0 | 0 | 0 | 2 | 0 | 0 | 0 | 0 | 0 | 0 | 0 | 16 | 0 | 2 | 3 |
| Methylocaldum | 16 | 1 | 22 | 17 | 20 | 13 | 26 | 23 | 20 | 1 | 19 | 8 | 4 | 64 | 2 | 4 |
| Methylocystis | 41 | 16 | 50 | 49 | 37 | 60 | 108 | 98 | 65 | 103 | 44 | 45 | 72 | 109 | 71 | 55 |
| Silvanigrella | 0 | 0 | 2 | 1 | 0 | 1 | 0 | 0 | 0 | 2 | 0 | 0 | 0 | 2 | 3 | 0 |
| Methylomagnum | 0 | 0 | 0 | 0 | 0 | 0 | 0 | 2 | 2 | 2 | 0 | 0 | 8 | 0 | 0 | 0 |
| Magnetovibrio | 0 | 0 | 0 | 0 | 3 | 0 | 0 | 0 | 0 | 0 | 0 | 15 | 13 | 0 | 0 | 0 |
| Mesorhizobium | 5 | 5 | 23 | 10 | 13 | 28 | 7 | 5 | 7 | 25 | 5 | 0 | 5 | 1 | 6 | 22 |
| Ruminococcaceae_UCG-010 | 0 | 2 | 0 | 3 | 0 | 0 | 0 | 0 | 0 | 0 | 0 | 0 | 0 | 0 | 0 | 0 |
| Paenisporosarcina | 2450 | 6 | 22 | 22 | 38 | 12 | 10 | 10 | 3 | 31 | 39 | 20 | 155 | 4 | 29 | 32 |
| Maritimimonas | 0 | 42 | 8 | 1 | 0 | 8 | 1 | 4 | 11 | 4 | 2 | 3 | 0 | 0 | 0 | 2 |
| Methanosarcina | 0 | 5 | 3 | 13 | 0 | 0 | 0 | 1 | 0 | 0 | 0 | 1 | 2 | 0 | 1 | 0 |
| Annamia_HOs24 | 2 | 0 | 0 | 0 | 1 | 1 | 0 | 2 | 0 | 1 | 0 | 0 | 1 | 0 | 0 | 0 |
| Natranaerovirga | 0 | 2 | 0 | 3 | 3 | 0 | 43 | 2 | 0 | 3 | 0 | 2 | 0 | 0 | 0 | 1 |
| Saccharofermentans | 2 | 11 | 1 | 14 | 1 | 7 | 4 | 6 | 11 | 1 | 0 | 24 | 5 | 1 | 6 | 8 |
| Sphaerospermopsis_BCCUSP55 | 1 | 17 | 0 | 2 | 3 | 0 | 1 | 0 | 2 | 0 | 2 | 0 | 0 | 0 | 0 | 0 |
| Algoriphagus | 0 | 17 | 9 | 0 | 0 | 0 | 0 | 0 | 1 | 0 | 2 | 1 | 1 | 0 | 1 | 0 |
| Nakamurella | 0 | 0 | 13 | 4 | 0 | 5 | 1 | 0 | 2 | 3 | 0 | 0 | 0 | 0 | 0 | 19 |
| Ruminococcaceae_UCG-012 | 1 | 8 | 0 | 0 | 2 | 2 | 1 | 4 | 3 | 0 | 7 | 2 | 1 | 2 | 0 | 1 |
| Anaerofustis | 0 | 2 | 0 | 3 | 0 | 3 | 0 | 0 | 0 | 0 | 0 | 0 | 0 | 0 | 0 | 0 |
| Microcystis_PCC-7914 | 0 | 0 | 0 | 0 | 0 | 4 | 0 | 1 | 2 | 2 | 0 | 0 | 0 | 0 | 0 | 1 |
| Aquabacterium | 0 | 0 | 0 | 1 | 0 | 0 | 2 | 0 | 0 | 2 | 3 | 0 | 0 | 0 | 0 | 0 |
| Microseira_Carmichael-Alabama | 1 | 0 | 0 | 0 | 0 | 0 | 0 | 2 | 2 | 16 | 0 | 0 | 0 | 0 | 0 | 0 |
| Desulfococcus | 0 | 0 | 0 | 1 | 2 | 0 | 0 | 1 | 0 | 1 | 4 | 3 | 0 | 0 | 0 | 1 |
| Prosthecobacter | 2 | 1 | 2 | 4 | 3 | 6 | 16 | 3 | 4 | 2 | 0 | 0 | 0 | 2 | 2 | 2 |
| Prevotella_9 | 0 | 101 | 0 | 2 | 0 | 0 | 1 | 0 | 0 | 0 | 0 | 0 | 0 | 1 | 1 | 6 |
| Burkholderia-Caballeronia-Paraburkholderia | 0 | 0 | 0 | 0 | 0 | 3 | 0 | 0 | 0 | 0 | 0 | 11 | 0 | 0 | 0 | 0 |
| Truepera | 127 | 8 | 2 | 248 | 13 | 6 | 186 | 4 | 6 | 11 | 3 | 2 | 19 | 10 | 9 | 30 |
| DSSD61 | 9 | 0 | 25 | 7 | 15 | 8 | 8 | 4 | 9 | 19 | 1 | 3 | 2 | 44 | 6 | 14 |
| Caedibacter | 0 | 1 | 0 | 5 | 0 | 1 | 3 | 2 | 0 | 1 | 1 | 0 | 0 | 0 | 0 | 2 |
| Demequina | 5 | 5 | 5 | 3 | 5 | 10 | 5 | 3 | 2 | 13 | 6 | 3 | 1 | 3 | 0 | 4 |
| Deefgea | 8 | 7 | 4 | 11 | 0 | 19 | 11 | 1 | 0 | 3 | 1 | 0 | 0 | 1 | 0 | 0 |
| Polynucleobacter | 2 | 7 | 5 | 0 | 0 | 4 | 0 | 1 | 0 | 0 | 0 | 2 | 7 | 0 | 0 | 0 |
| Pontibacter | 5 | 3 | 0 | 0 | 0 | 1 | 1 | 3 | 0 | 1 | 2 | 0 | 7 | 1 | 2 | 0 |
| Exiguobacterium | 188 | 9 | 1 | 4 | 2 | 6 | 6 | 5 | 4 | 1 | 2 | 1 | 3 | 0 | 0 | 13 |
| Rhodoplanes | 4 | 3 | 16 | 3 | 4 | 7 | 1 | 2 | 1 | 14 | 4 | 1 | 13 | 2 | 1 | 22 |
| Porphyrobacter | 3 | 13 | 233 | 14 | 36 | 104 | 67 | 10 | 6 | 14 | 14 | 10 | 3 | 1 | 8 | 8 |
| Brevifollis | 0 | 2 | 0 | 0 | 0 | 0 | 2 | 0 | 0 | 0 | 0 | 0 | 0 | 0 | 0 | 0 |
| Prevotella_7 | 0 | 24 | 0 | 0 | 0 | 0 | 0 | 0 | 0 | 0 | 0 | 4 | 0 | 0 | 0 | 1 |
| Portibacter | 3 | 1 | 2 | 40 | 2 | 7 | 4 | 4 | 7 | 9 | 0 | 0 | 0 | 4 | 3 | 5 |
| Porticoccus | 0 | 0 | 0 | 0 | 1 | 3 | 1 | 0 | 0 | 0 | 0 | 3 | 0 | 0 | 4 | 15 |
| Caulobacter | 3 | 0 | 2 | 0 | 5 | 0 | 5 | 1 | 0 | 0 | 0 | 0 | 0 | 0 | 0 | 1 |
| pLW-20 | 2 | 5 | 0 | 0 | 0 | 0 | 1 | 0 | 0 | 0 | 0 | 0 | 0 | 0 | 0 | 0 |
| Chitinibacter | 0 | 2 | 1 | 2 | 0 | 6 | 8 | 1 | 2 | 1 | 0 | 1 | 0 | 0 | 6 | 2 |
| Cellulosilyticum | 4 | 0 | 0 | 0 | 0 | 0 | 0 | 0 | 0 | 1 | 0 | 0 | 4 | 0 | 0 | 0 |
| Candidatus_Methanoperedens | 1 | 18 | 8 | 8 | 0 | 0 | 8 | 1 | 2 | 1 | 4 | 0 | 104 | 1 | 13 | 0 |
| Candidatus_Entotheonella | 0 | 1 | 0 | 0 | 0 | 0 | 0 | 1 | 0 | 0 | 1 | 0 | 1 | 0 | 0 | 6 |
| Candidatus_Contendobacter | 0 | 0 | 0 | 0 | 0 | 1 | 0 | 0 | 0 | 0 | 0 | 8 | 0 | 0 | 0 | 0 |
| Pseudorhodoplanes | 30 | 36 | 65 | 42 | 44 | 43 | 25 | 23 | 12 | 90 | 29 | 15 | 20 | 26 | 12 | 64 |
| Candidatus_Jidaibacter | 0 | 0 | 0 | 0 | 0 | 0 | 4 | 0 | 0 | 0 | 0 | 2 | 0 | 0 | 0 | 0 |
| Alkaliphilus | 2 | 1 | 0 | 0 | 0 | 0 | 0 | 0 | 0 | 0 | 0 | 3 | 3 | 1 | 0 | 0 |
| Cohnella | 0 | 4 | 0 | 0 | 0 | 0 | 0 | 0 | 0 | 0 | 1 | 2 | 7 | 0 | 0 | 0 |
| Urania-1B-19_  marine_sediment_group | 0 | 0 | 0 | 2 | 1 | 0 | 0 | 0 | 0 | 1 | 2 | 0 | 2 | 0 | 0 | 0 |
| Cryptanaerobacter | 1 | 352 | 2 | 10 | 2 | 7 | 17 | 6 | 2 | 0 | 2 | 64 | 5 | 0 | 44 | 0 |
| Clostridium_sensu_stricto_6 | 1 | 2 | 1 | 2 | 1 | 2 | 0 | 2 | 1 | 0 | 1 | 2 | 1 | 1 | 4 | 1 |
| Chryseolinea | 88 | 34 | 170 | 67 | 77 | 41 | 31 | 61 | 9 | 64 | 19 | 23 | 9 | 76 | 19 | 31 |
| Candidatus_Anammoximicrobium | 0 | 0 | 4 | 0 | 0 | 2 | 2 | 2 | 1 | 1 | 5 | 0 | 0 | 0 | 1 | 2 |
| Wolinella | 2 | 0 | 0 | 1 | 1 | 1 | 1 | 1 | 2 | 2 | 3 | 0 | 0 | 3 | 0 | 2 |
| Clostridium_sensu_stricto_11 | 4 | 73 | 4 | 1 | 1 | 0 | 0 | 1 | 3 | 4 | 8 | 9 | 2 | 4 | 12 | 1 |
| Bosea | 1 | 4 | 15 | 9 | 1 | 4 | 2 | 6 | 1 | 6 | 6 | 2 | 3 | 0 | 4 | 7 |
| Actinomadura | 0 | 0 | 3 | 0 | 0 | 0 | 0 | 0 | 0 | 0 | 0 | 0 | 6 | 0 | 0 | 0 |
| HdN1 | 0 | 0 | 0 | 0 | 5 | 0 | 0 | 0 | 0 | 0 | 0 | 1 | 0 | 0 | 5 | 1 |
| Sulfuriferula | 4 | 0 | 6 | 4 | 8 | 2 | 14 | 5 | 0 | 5 | 3 | 59 | 18 | 2 | 25 | 5 |
| Actibacter | 47 | 208 | 72 | 443 | 74 | 394 | 321 | 539 | 419 | 600 | 113 | 161 | 101 | 145 | 211 | 178 |
| Synechocystis_PCC-6803 | 0 | 1 | 0 | 0 | 0 | 0 | 5 | 0 | 2 | 0 | 0 | 0 | 0 | 0 | 0 | 0 |
| Sulfurospirillum | 0 | 12 | 0 | 0 | 0 | 0 | 10 | 0 | 0 | 1 | 0 | 6 | 0 | 0 | 0 | 0 |
| Hyphomonas | 5 | 5 | 7 | 42 | 9 | 24 | 15 | 21 | 17 | 14 | 16 | 10 | 0 | 10 | 3 | 19 |
| IheB3-7 | 0 | 2 | 4 | 1 | 1 | 2 | 0 | 3 | 0 | 3 | 2 | 0 | 0 | 1 | 0 | 5 |
| BIyi10 | 0 | 0 | 3 | 0 | 4 | 0 | 0 | 0 | 0 | 0 | 0 | 0 | 0 | 1 | 5 | 2 |
| KCM-B-112 | 0 | 0 | 5 | 0 | 0 | 1 | 0 | 0 | 0 | 0 | 0 | 19 | 0 | 0 | 0 | 0 |
| Holophaga | 0 | 0 | 0 | 0 | 0 | 2 | 0 | 1 | 2 | 2 | 5 | 2 | 0 | 0 | 0 | 1 |
| Pelosinus | 1 | 0 | 0 | 1 | 0 | 0 | 0 | 0 | 0 | 0 | 1 | 2 | 0 | 0 | 5 | 0 |
| Labrys | 0 | 0 | 7 | 0 | 11 | 9 | 4 | 0 | 0 | 3 | 0 | 0 | 3 | 1 | 0 | 2 |
| FukuN18_freshwater_group | 0 | 0 | 0 | 0 | 0 | 0 | 0 | 0 | 0 | 0 | 0 | 0 | 2 | 2 | 1 | 0 |
| Romboutsia | 54 | 18 | 19 | 68 | 13 | 14 | 4 | 17 | 21 | 13 | 38 | 165 | 21 | 15 | 120 | 49 |
| Terrimicrobium | 0 | 3 | 5 | 8 | 0 | 7 | 5 | 2 | 1 | 1 | 0 | 2 | 2 | 1 | 0 | 0 |
| Pedobacter | 0 | 2 | 5 | 0 | 14 | 4 | 1 | 1 | 0 | 3 | 3 | 0 | 3 | 0 | 2 | 0 |
| Sporobacter | 0 | 9 | 0 | 0 | 4 | 3 | 0 | 0 | 0 | 2 | 0 | 1 | 0 | 0 | 1 | 0 |
| LD29 | 0 | 0 | 15 | 1 | 2 | 0 | 1 | 0 | 2 | 0 | 0 | 0 | 0 | 1 | 1 | 0 |
| Gallionella | 3 | 18 | 2 | 0 | 0 | 0 | 0 | 0 | 0 | 0 | 0 | 4 | 12 | 0 | 0 | 0 |
| Ga0074140 | 0 | 0 | 0 | 0 | 2 | 0 | 0 | 0 | 0 | 0 | 0 | 0 | 8 | 0 | 0 | 0 |
| Kribbella | 0 | 4 | 0 | 1 | 0 | 0 | 0 | 0 | 0 | 10 | 0 | 1 | 1 | 0 | 0 | 8 |
| Rubrobacter | 1 | 5 | 0 | 0 | 0 | 0 | 0 | 0 | 0 | 1 | 0 | 0 | 0 | 0 | 0 | 2 |
| Roseococcus | 0 | 0 | 3 | 0 | 12 | 2 | 9 | 2 | 0 | 4 | 0 | 2 | 0 | 0 | 1 | 0 |
| Rubritalea | 0 | 2 | 0 | 0 | 0 | 0 | 0 | 0 | 0 | 0 | 0 | 0 | 0 | 0 | 0 | 0 |
| Allokutzneria | 0 | 0 | 2 | 0 | 0 | 0 | 0 | 0 | 0 | 0 | 0 | 0 | 2 | 0 | 0 | 2 |
| Rubritepida | 0 | 0 | 0 | 3 | 1 | 0 | 0 | 0 | 1 | 3 | 0 | 0 | 0 | 0 | 0 | 1 |
| Rubellimicrobium | 0 | 0 | 0 | 0 | 0 | 0 | 0 | 0 | 0 | 0 | 0 | 0 | 0 | 0 | 0 | 5 |
| Rubribacterium | 0 | 0 | 0 | 0 | 3 | 0 | 4 | 0 | 0 | 0 | 1 | 0 | 0 | 0 | 0 | 0 |
| Alicyclobacillus | 0 | 0 | 0 | 0 | 0 | 0 | 0 | 2 | 0 | 0 | 0 | 0 | 0 | 0 | 0 | 0 |
| Puniceicoccus | 0 | 0 | 0 | 0 | 2 | 0 | 0 | 0 | 2 | 0 | 0 | 0 | 0 | 0 | 0 | 4 |
| Allocatelliglobosispora | 0 | 0 | 13 | 0 | 0 | 1 | 0 | 1 | 0 | 0 | 0 | 0 | 0 | 1 | 0 | 0 |
| Roseiarcus | 0 | 0 | 1 | 0 | 0 | 8 | 0 | 0 | 0 | 0 | 0 | 0 | 0 | 0 | 0 | 0 |
| Ruminococcaceae_UCG-005 | 0 | 0 | 0 | 0 | 0 | 0 | 0 | 0 | 0 | 0 | 3 | 0 | 0 | 0 | 0 | 0 |
| Roseiflexus | 3 | 0 | 0 | 0 | 0 | 0 | 2 | 0 | 0 | 0 | 0 | 0 | 0 | 0 | 0 | 0 |
| Rhodocytophaga | 0 | 4 | 0 | 0 | 0 | 1 | 0 | 0 | 0 | 0 | 0 | 0 | 0 | 0 | 0 | 0 |
| Rhodopirellula | 0 | 0 | 0 | 3 | 1 | 0 | 3 | 0 | 0 | 0 | 0 | 0 | 0 | 1 | 0 | 0 |
| Rickettsiella | 0 | 0 | 2 | 0 | 0 | 4 | 0 | 2 | 1 | 3 | 2 | 4 | 0 | 0 | 0 | 0 |
| Rivibacter | 0 | 0 | 0 | 0 | 0 | 0 | 2 | 0 | 0 | 0 | 0 | 0 | 0 | 0 | 0 | 0 |
| Rikenellaceae_RC9  _gut_group | 0 | 0 | 0 | 0 | 0 | 2 | 0 | 0 | 0 | 0 | 0 | 0 | 0 | 0 | 0 | 0 |
| Rhodoblastus | 0 | 0 | 36 | 0 | 3 | 0 | 2 | 0 | 0 | 0 | 3 | 0 | 1 | 3 | 0 | 1 |
| Ramlibacter | 0 | 0 | 0 | 0 | 0 | 2 | 0 | 0 | 1 | 0 | 0 | 0 | 0 | 0 | 0 | 0 |
| Ruminococcaceae_  NK4A214_group | 0 | 0 | 0 | 3 | 0 | 0 | 0 | 0 | 0 | 0 | 0 | 0 | 0 | 0 | 0 | 0 |
| Rahnella | 0 | 0 | 0 | 0 | 0 | 0 | 0 | 4 | 0 | 0 | 0 | 0 | 0 | 0 | 0 | 0 |
| Ralstonia | 11 | 0 | 0 | 0 | 0 | 4 | 0 | 1 | 0 | 0 | 7 | 0 | 0 | 0 | 0 | 0 |
| Ruminococcaceae_  UCG-013 | 1 | 0 | 0 | 0 | 0 | 0 | 1 | 0 | 0 | 0 | 0 | 1 | 3 | 0 | 0 | 0 |
| Rhizomicrobium | 0 | 0 | 1 | 0 | 2 | 3 | 0 | 12 | 0 | 14 | 0 | 1 | 0 | 0 | 0 | 2 |
| Alistipes | 0 | 6 | 0 | 9 | 0 | 0 | 0 | 1 | 0 | 0 | 0 | 0 | 0 | 0 | 1 | 0 |
| Rheinheimera | 0 | 0 | 0 | 1 | 0 | 5 | 0 | 2 | 0 | 0 | 0 | 0 | 0 | 1 | 0 | 0 |
| Thiodictyon | 0 | 0 | 11 | 0 | 0 | 0 | 0 | 0 | 1 | 0 | 0 | 0 | 0 | 0 | 0 | 0 |
| Thiopseudomonas | 0 | 0 | 0 | 0 | 0 | 3 | 0 | 0 | 0 | 0 | 0 | 0 | 0 | 0 | 1 | 0 |
| Thiocapsa | 0 | 0 | 1 | 4 | 0 | 0 | 1 | 0 | 0 | 0 | 0 | 1 | 0 | 0 | 0 | 0 |
| Thermomonas | 0 | 2 | 5 | 1 | 4 | 0 | 0 | 0 | 0 | 0 | 3 | 2 | 0 | 0 | 0 | 0 |
| Achromobacter | 0 | 1 | 4 | 1 | 1 | 3 | 0 | 3 | 0 | 1 | 0 | 0 | 0 | 1 | 5 | 1 |
| Tolypothrix_PCC-7601 | 2 | 2 | 2 | 0 | 0 | 0 | 0 | 0 | 3 | 0 | 3 | 0 | 0 | 0 | 1 | 0 |
| Achromatium | 0 | 0 | 0 | 0 | 0 | 0 | 2 | 0 | 0 | 0 | 0 | 0 | 0 | 0 | 0 | 0 |
| Tolumonas | 1 | 11 | 0 | 0 | 3 | 0 | 0 | 0 | 0 | 0 | 0 | 0 | 1 | 0 | 0 | 0 |
| Thiovirga | 0 | 0 | 2 | 0 | 0 | 0 | 0 | 0 | 0 | 0 | 0 | 0 | 0 | 0 | 0 | 0 |
| Tissierella | 0 | 0 | 0 | 0 | 0 | 0 | 0 | 0 | 0 | 0 | 1 | 0 | 0 | 0 | 3 | 0 |
| Telmatocola | 0 | 0 | 0 | 0 | 0 | 0 | 0 | 2 | 0 | 0 | 0 | 0 | 0 | 0 | 0 | 0 |
| Tepidimicrobium | 0 | 0 | 0 | 0 | 0 | 0 | 0 | 0 | 0 | 0 | 0 | 0 | 0 | 0 | 2 | 0 |
| Taonella | 0 | 0 | 0 | 0 | 2 | 0 | 2 | 0 | 0 | 3 | 0 | 1 | 0 | 0 | 0 | 0 |
| Tahibacter | 0 | 1 | 0 | 1 | 0 | 0 | 0 | 0 | 0 | 1 | 0 | 1 | 0 | 0 | 0 | 0 |
| Taibaiella | 4 | 1 | 0 | 7 | 9 | 1 | 1 | 0 | 0 | 0 | 0 | 0 | 0 | 0 | 0 | 0 |
| Thermoflavimicrobium | 0 | 0 | 0 | 0 | 0 | 0 | 0 | 0 | 0 | 4 | 0 | 0 | 0 | 0 | 1 | 0 |
| Thermoflexibacter | 0 | 3 | 0 | 0 | 0 | 0 | 0 | 0 | 0 | 0 | 0 | 0 | 0 | 0 | 0 | 0 |
| Terrisporobacter | 2 | 1 | 4 | 1 | 1 | 0 | 2 | 0 | 1 | 4 | 0 | 8 | 9 | 1 | 2 | 1 |
| Tepidisphaera | 0 | 0 | 0 | 0 | 0 | 0 | 4 | 3 | 0 | 0 | 0 | 0 | 0 | 0 | 0 | 1 |
| Acidipila | 0 | 2 | 0 | 0 | 0 | 0 | 0 | 0 | 0 | 0 | 0 | 0 | 0 | 0 | 0 | 0 |
| Treponema_2 | 2 | 5 | 4 | 6 | 3 | 2 | 5 | 5 | 3 | 3 | 0 | 6 | 9 | 0 | 1 | 7 |
| Wolbachia | 0 | 0 | 0 | 0 | 0 | 0 | 0 | 0 | 0 | 0 | 0 | 0 | 0 | 0 | 2 | 0 |
| XBB1006 | 0 | 0 | 0 | 6 | 0 | 0 | 0 | 0 | 0 | 0 | 0 | 0 | 0 | 0 | 0 | 0 |
| Vogesella | 0 | 0 | 0 | 0 | 0 | 3 | 0 | 2 | 0 | 0 | 0 | 0 | 0 | 0 | 0 | 0 |
| Verticia | 0 | 6 | 0 | 0 | 0 | 0 | 0 | 0 | 0 | 0 | 0 | 0 | 0 | 0 | 0 | 0 |
| Vicinamibacter | 0 | 0 | 0 | 0 | 0 | 0 | 0 | 0 | 0 | 3 | 0 | 0 | 0 | 0 | 0 | 4 |
| possible_genus_04 | 0 | 0 | 0 | 0 | 11 | 0 | 3 | 0 | 0 | 0 | 0 | 1 | 0 | 4 | 0 | 0 |
| 1174-901-12 | 0 | 1 | 0 | 0 | 0 | 2 | 0 | 0 | 0 | 2 | 0 | 0 | 0 | 0 | 0 | 0 |
| possible_genus_03 | 0 | 0 | 0 | 1 | 0 | 1 | 0 | 1 | 5 | 4 | 0 | 1 | 3 | 1 | 2 | 0 |
| ZOR0006 | 0 | 0 | 0 | 0 | 1 | 0 | 6 | 1 | 0 | 0 | 0 | 0 | 0 | 0 | 0 | 0 |
| Acetobacterium | 4 | 3 | 3 | 3 | 0 | 3 | 0 | 2 | 2 | 3 | 0 | 2 | 0 | 0 | 0 | 2 |
| Turicibacter | 1 | 3 | 6 | 0 | 2 | 1 | 0 | 2 | 0 | 0 | 2 | 3 | 9 | 0 | 1 | 0 |
| Tychonema_CCAP_1459-11B | 0 | 0 | 21 | 0 | 0 | 0 | 8 | 11 | 38 | 4 | 0 | 0 | 0 | 0 | 2 | 0 |
| Tsukamurella | 0 | 0 | 3 | 0 | 0 | 0 | 0 | 0 | 0 | 0 | 0 | 0 | 2 | 0 | 0 | 0 |
| Trichococcus | 20 | 417 | 12 | 40 | 3 | 36 | 3 | 23 | 2 | 3 | 9 | 73 | 4 | 3 | 6 | 4 |
| Tropicimonas | 6 | 3 | 5 | 17 | 12 | 6 | 57 | 12 | 8 | 15 | 5 | 2 | 1 | 3 | 7 | 5 |
| Verruc-01 | 0 | 0 | 0 | 0 | 0 | 0 | 0 | 0 | 0 | 1 | 0 | 2 | 0 | 0 | 0 | 0 |
| Verrucomicrobium | 0 | 0 | 0 | 0 | 0 | 0 | 0 | 0 | 0 | 0 | 0 | 0 | 0 | 0 | 3 | 0 |
| Undibacterium | 0 | 0 | 10 | 0 | 0 | 0 | 4 | 0 | 0 | 0 | 1 | 0 | 0 | 0 | 0 | 1 |
| Tyzzerella_3 | 0 | 0 | 0 | 0 | 0 | 0 | 0 | 0 | 0 | 0 | 2 | 0 | 0 | 0 | 0 | 0 |
| UTCFX1 | 4 | 7 | 12 | 3 | 8 | 2 | 1 | 2 | 3 | 30 | 6 | 6 | 34 | 3 | 3 | 15 |
| Sediminispirochaeta | 1 | 0 | 0 | 1 | 0 | 0 | 1 | 0 | 1 | 0 | 0 | 3 | 0 | 0 | 0 | 3 |
| Advenella | 0 | 0 | 0 | 0 | 0 | 0 | 0 | 0 | 0 | 2 | 0 | 0 | 0 | 0 | 0 | 0 |
| Schwartzia | 0 | 9 | 0 | 0 | 0 | 0 | 0 | 0 | 0 | 0 | 0 | 0 | 0 | 0 | 0 | 0 |
| Schleiferia | 0 | 0 | 0 | 0 | 1 | 0 | 0 | 0 | 0 | 0 | 0 | 0 | 0 | 8 | 0 | 0 |
| Schlesneria | 0 | 0 | 0 | 2 | 0 | 0 | 0 | 1 | 0 | 0 | 0 | 0 | 0 | 0 | 1 | 0 |
| Sorangium | 0 | 0 | 1 | 0 | 0 | 0 | 0 | 0 | 0 | 0 | 0 | 2 | 0 | 0 | 0 | 0 |
| Sphingoaurantiacus | 1 | 0 | 0 | 0 | 0 | 0 | 0 | 0 | 0 | 3 | 0 | 0 | 1 | 0 | 0 | 1 |
| Solobacterium | 0 | 3 | 0 | 0 | 0 | 0 | 0 | 0 | 0 | 0 | 0 | 1 | 0 | 0 | 0 | 0 |
| Shimazuella | 0 | 3 | 0 | 0 | 0 | 0 | 0 | 0 | 0 | 0 | 0 | 0 | 0 | 0 | 0 | 0 |
| Solitalea | 0 | 3 | 0 | 3 | 0 | 0 | 0 | 0 | 0 | 0 | 0 | 0 | 0 | 0 | 2 | 0 |
| Aggregicoccus | 0 | 0 | 0 | 0 | 0 | 0 | 8 | 0 | 0 | 0 | 14 | 0 | 0 | 1 | 1 | 1 |
| Salinispora | 0 | 0 | 0 | 0 | 0 | 0 | 0 | 0 | 0 | 6 | 0 | 0 | 0 | 0 | 1 | 0 |
| SU2_symbiont_group | 0 | 0 | 1 | 0 | 0 | 0 | 3 | 4 | 1 | 29 | 0 | 0 | 0 | 0 | 0 | 1 |
| SCADC1-2-3 | 0 | 4 | 0 | 0 | 0 | 0 | 0 | 0 | 0 | 0 | 0 | 2 | 0 | 0 | 0 | 0 |
| SEEP-SRB1 | 0 | 0 | 0 | 0 | 0 | 0 | 0 | 0 | 0 | 3 | 0 | 1 | 2 | 0 | 0 | 0 |
| Sarcina | 0 | 0 | 2 | 0 | 0 | 0 | 0 | 3 | 0 | 0 | 0 | 1 | 0 | 0 | 1 | 0 |
| Schizothrix_LEGE_07164 | 0 | 0 | 0 | 3 | 0 | 0 | 0 | 0 | 0 | 0 | 0 | 0 | 0 | 0 | 0 | 0 |
| Sanguibacter | 0 | 0 | 0 | 3 | 0 | 1 | 0 | 0 | 0 | 0 | 0 | 0 | 0 | 0 | 0 | 1 |
| Sandaracinobacter | 0 | 0 | 0 | 0 | 6 | 2 | 4 | 0 | 2 | 3 | 0 | 0 | 1 | 0 | 0 | 3 |
| Sandarakinorhabdus | 0 | 0 | 2 | 0 | 0 | 5 | 13 | 0 | 0 | 7 | 1 | 0 | 1 | 3 | 4 | 2 |
| Actinotalea | 0 | 1 | 2 | 0 | 0 | 0 | 0 | 0 | 0 | 5 | 1 | 2 | 1 | 2 | 0 | 0 |
| Actinomycetospora | 0 | 0 | 0 | 0 | 0 | 2 | 0 | 0 | 0 | 3 | 0 | 0 | 0 | 0 | 0 | 0 |
| Actinoallomurus | 0 | 0 | 0 | 0 | 0 | 0 | 0 | 0 | 0 | 2 | 0 | 0 | 0 | 0 | 0 | 0 |
| Actinophytocola | 0 | 3 | 0 | 0 | 0 | 0 | 0 | 0 | 0 | 1 | 0 | 0 | 0 | 0 | 0 | 2 |
| Streptosporangium | 0 | 0 | 1 | 0 | 3 | 3 | 0 | 0 | 0 | 0 | 0 | 2 | 0 | 0 | 0 | 0 |
| Actinoplanes | 0 | 2 | 3 | 0 | 1 | 0 | 1 | 1 | 0 | 2 | 0 | 0 | 0 | 0 | 0 | 9 |
| Tabrizicola | 0 | 0 | 2 | 0 | 0 | 0 | 1 | 1 | 0 | 0 | 0 | 0 | 1 | 0 | 0 | 0 |
| Tagaea | 5 | 0 | 1 | 0 | 4 | 4 | 0 | 0 | 0 | 3 | 2 | 0 | 16 | 0 | 0 | 1 |
| Acidothermus | 0 | 0 | 2 | 0 | 0 | 0 | 0 | 0 | 0 | 0 | 0 | 0 | 0 | 0 | 0 | 0 |
| Symphothece_PCC-7002 | 0 | 0 | 0 | 0 | 0 | 2 | 0 | 0 | 0 | 0 | 0 | 0 | 0 | 0 | 0 | 0 |
| Acidovorax | 0 | 0 | 0 | 0 | 0 | 0 | 0 | 3 | 0 | 0 | 0 | 0 | 0 | 0 | 0 | 0 |
| Sporacetigenium | 15 | 10 | 3 | 3 | 3 | 8 | 2 | 1 | 4 | 0 | 14 | 17 | 36 | 5 | 76 | 25 |
| Sporichthya | 1 | 0 | 15 | 17 | 1 | 1 | 4 | 1 | 0 | 6 | 0 | 0 | 0 | 0 | 4 | 3 |
| Sphingosinicella | 0 | 0 | 1 | 0 | 2 | 0 | 0 | 0 | 0 | 1 | 2 | 2 | 0 | 0 | 0 | 0 |
| Sphingopyxis | 0 | 15 | 0 | 4 | 1 | 0 | 8 | 0 | 0 | 18 | 3 | 0 | 3 | 2 | 3 | 5 |
| Sphingorhabdus | 0 | 2 | 0 | 0 | 0 | 0 | 0 | 0 | 0 | 0 | 0 | 0 | 0 | 0 | 0 | 0 |
| Sterolibacterium | 0 | 0 | 1 | 2 | 0 | 0 | 0 | 0 | 1 | 0 | 0 | 3 | 24 | 1 | 0 | 0 |
| Actinopolymorpha | 0 | 0 | 0 | 0 | 0 | 0 | 0 | 0 | 0 | 0 | 2 | 0 | 0 | 0 | 0 | 0 |
| Stenotrophomonas | 0 | 0 | 0 | 0 | 0 | 0 | 0 | 0 | 0 | 0 | 0 | 2 | 0 | 0 | 0 | 0 |
| Sporosarcina | 0 | 0 | 1 | 2 | 0 | 0 | 0 | 1 | 0 | 0 | 0 | 0 | 2 | 0 | 0 | 2 |
| Stella | 0 | 1 | 12 | 1 | 14 | 1 | 2 | 0 | 0 | 2 | 1 | 1 | 0 | 0 | 0 | 0 |
| Psychrobacter | 2 | 0 | 0 | 0 | 0 | 0 | 0 | 0 | 0 | 0 | 0 | 0 | 0 | 0 | 0 | 0 |
| Fodinibacter | 0 | 0 | 10 | 1 | 0 | 0 | 0 | 0 | 0 | 1 | 0 | 1 | 1 | 0 | 0 | 3 |
| Fodinicola | 4 | 2 | 50 | 37 | 3 | 15 | 41 | 9 | 3 | 8 | 7 | 0 | 0 | 4 | 2 | 15 |
| Fluviimonas | 1 | 0 | 7 | 0 | 0 | 0 | 0 | 0 | 0 | 0 | 0 | 0 | 0 | 0 | 0 | 0 |
| Flexibacter | 0 | 0 | 0 | 1 | 0 | 0 | 12 | 0 | 0 | 0 | 0 | 0 | 0 | 1 | 0 | 3 |
| Fluviicoccus | 0 | 2 | 1 | 0 | 0 | 0 | 0 | 0 | 0 | 0 | 0 | 0 | 0 | 0 | 0 | 2 |
| Formivibrio | 2 | 3 | 0 | 0 | 0 | 2 | 1 | 0 | 0 | 0 | 0 | 1 | 0 | 0 | 0 | 1 |
| Geitlerinema_PCC-7105 | 0 | 0 | 5 | 0 | 1 | 0 | 0 | 0 | 0 | 0 | 0 | 0 | 1 | 0 | 0 | 4 |
| Geitlerinema_PCC-8501 | 0 | 0 | 0 | 0 | 0 | 0 | 4 | 0 | 3 | 0 | 0 | 0 | 0 | 0 | 0 | 0 |
| GWE2-31-10 | 0 | 2 | 1 | 0 | 0 | 1 | 0 | 0 | 0 | 1 | 1 | 1 | 0 | 0 | 0 | 1 |
| FukuN57 | 0 | 0 | 0 | 0 | 0 | 0 | 0 | 0 | 2 | 0 | 0 | 0 | 0 | 0 | 0 | 0 |
| GKS98_freshwater_group | 0 | 0 | 0 | 2 | 0 | 0 | 0 | 0 | 0 | 0 | 0 | 0 | 3 | 0 | 0 | 0 |
| Flavitalea | 0 | 0 | 0 | 0 | 0 | 0 | 0 | 2 | 0 | 0 | 0 | 0 | 0 | 0 | 0 | 0 |
| Bradyrhizobium | 10 | 41 | 152 | 30 | 54 | 111 | 16 | 15 | 5 | 96 | 22 | 15 | 23 | 15 | 9 | 101 |
| Elstera | 0 | 0 | 0 | 0 | 0 | 0 | 0 | 0 | 0 | 0 | 0 | 0 | 0 | 0 | 0 | 2 |
| Elev-16S-1166 | 5 | 5 | 2 | 3 | 16 | 3 | 12 | 6 | 0 | 2 | 2 | 2 | 0 | 25 | 1 | 3 |
| Brevibacillus | 0 | 0 | 0 | 0 | 0 | 0 | 0 | 0 | 1 | 6 | 0 | 0 | 0 | 0 | 0 | 3 |
| Dyella | 2 | 0 | 2 | 0 | 0 | 2 | 0 | 0 | 0 | 0 | 0 | 0 | 0 | 0 | 0 | 0 |
| Enterovibrio | 0 | 0 | 0 | 0 | 0 | 0 | 5 | 0 | 0 | 0 | 0 | 0 | 0 | 0 | 0 | 0 |
| Fervidicella | 0 | 0 | 0 | 1 | 0 | 0 | 0 | 1 | 0 | 0 | 0 | 7 | 4 | 1 | 0 | 0 |
| Fictibacillus | 52 | 1 | 2 | 2 | 3 | 4 | 0 | 4 | 0 | 0 | 9 | 2 | 65 | 4 | 1 | 7 |
| Family_XIII_UCG-001 | 0 | 0 | 0 | 0 | 0 | 1 | 1 | 0 | 0 | 0 | 0 | 4 | 0 | 0 | 0 | 0 |
| Ercella | 0 | 0 | 0 | 0 | 0 | 3 | 0 | 0 | 0 | 0 | 0 | 0 | 0 | 0 | 0 | 0 |
| Brachyspira | 0 | 0 | 0 | 0 | 0 | 0 | 0 | 0 | 0 | 0 | 0 | 0 | 0 | 0 | 0 | 3 |
| I-8 | 0 | 2 | 0 | 0 | 0 | 0 | 0 | 0 | 0 | 0 | 0 | 0 | 0 | 0 | 0 | 0 |
| Azospirillum | 0 | 2 | 0 | 0 | 0 | 0 | 0 | 0 | 0 | 0 | 0 | 0 | 0 | 0 | 0 | 0 |
| Hymenobacter | 0 | 3 | 0 | 0 | 0 | 0 | 0 | 0 | 0 | 0 | 0 | 0 | 0 | 0 | 0 | 0 |
| Herpetosiphon | 0 | 1 | 0 | 2 | 0 | 0 | 0 | 0 | 0 | 0 | 0 | 0 | 0 | 0 | 3 | 0 |
| Hydrogenoanaerobacterium | 0 | 0 | 0 | 1 | 0 | 0 | 0 | 3 | 0 | 0 | 0 | 0 | 0 | 0 | 0 | 0 |
| Immundisolibacter | 0 | 0 | 0 | 0 | 0 | 0 | 0 | 0 | 0 | 0 | 0 | 2 | 0 | 0 | 0 | 0 |
| JTB255_marine_benthic_group | 0 | 0 | 0 | 0 | 0 | 1 | 0 | 2 | 0 | 3 | 0 | 0 | 0 | 0 | 0 | 0 |
| Kamptonema_PCC-6407 | 0 | 0 | 3 | 0 | 0 | 0 | 0 | 0 | 0 | 0 | 0 | 0 | 0 | 0 | 0 | 0 |
| JGI-0000079-D21 | 0 | 3 | 0 | 0 | 0 | 0 | 0 | 0 | 0 | 0 | 1 | 4 | 0 | 0 | 0 | 0 |
| Inhella | 6 | 2 | 7 | 14 | 3 | 1 | 30 | 3 | 0 | 2 | 1 | 0 | 1 | 5 | 3 | 4 |
| Intrasporangium | 0 | 0 | 0 | 0 | 0 | 0 | 0 | 0 | 0 | 0 | 0 | 0 | 0 | 0 | 0 | 15 |
| Haloplasma | 0 | 0 | 0 | 0 | 0 | 0 | 0 | 0 | 0 | 0 | 2 | 0 | 0 | 0 | 0 | 0 |
| Blastococcus | 9 | 5 | 13 | 7 | 2 | 5 | 1 | 5 | 2 | 26 | 7 | 0 | 3 | 1 | 0 | 24 |
| Bellilinea | 6 | 7 | 0 | 0 | 0 | 0 | 0 | 0 | 1 | 0 | 0 | 2 | 11 | 0 | 8 | 2 |
| Gemmata | 0 | 0 | 2 | 0 | 0 | 0 | 0 | 0 | 0 | 0 | 0 | 0 | 0 | 0 | 0 | 0 |
| Geminicoccus | 0 | 1 | 6 | 0 | 0 | 1 | 0 | 0 | 0 | 0 | 1 | 0 | 0 | 0 | 0 | 0 |
| Geminocystis_PCC-6308 | 15 | 0 | 1 | 2 | 4 | 2 | 1 | 2 | 5 | 0 | 0 | 1 | 0 | 1 | 0 | 0 |
| Geothermomicrobium | 0 | 2 | 0 | 0 | 0 | 0 | 0 | 0 | 0 | 0 | 0 | 0 | 0 | 0 | 0 | 0 |
| Hahella | 2 | 0 | 0 | 0 | 0 | 0 | 1 | 0 | 2 | 0 | 0 | 0 | 0 | 3 | 0 | 4 |
| Haloimpatiens | 0 | 0 | 0 | 3 | 0 | 0 | 1 | 4 | 0 | 0 | 0 | 0 | 0 | 1 | 0 | 0 |
| Gottschalkia | 0 | 1 | 42 | 0 | 0 | 2 | 0 | 2 | 1 | 0 | 1 | 7 | 0 | 0 | 0 | 1 |
| AKIW659 | 0 | 0 | 0 | 5 | 2 | 0 | 0 | 0 | 0 | 0 | 0 | 0 | 0 | 0 | 0 | 0 |
| Gleocapsa | 0 | 0 | 0 | 0 | 0 | 0 | 2 | 0 | 0 | 0 | 0 | 0 | 0 | 0 | 0 | 0 |
| Clostridium_sensu_stricto_5 | 9 | 8 | 2 | 12 | 0 | 0 | 7 | 2 | 5 | 1 | 6 | 12 | 8 | 5 | 21 | 3 |
| Clostridium_sensu_stricto_7 | 0 | 0 | 0 | 0 | 2 | 0 | 0 | 0 | 0 | 0 | 0 | 0 | 0 | 0 | 0 | 0 |
| Clostridium_sensu_stricto_2 | 0 | 1 | 0 | 3 | 4 | 3 | 0 | 5 | 1 | 1 | 0 | 11 | 2 | 6 | 5 | 6 |
| Calothrix_PCC-6303 | 1 | 0 | 1 | 0 | 0 | 0 | 3 | 0 | 0 | 0 | 0 | 0 | 0 | 0 | 0 | 0 |
| Clostridium_sensu_stricto_14 | 2 | 0 | 1 | 12 | 1 | 1 | 0 | 3 | 3 | 2 | 5 | 1 | 1 | 2 | 3 | 3 |
| Clostridium_sensu_stricto_9 | 0 | 0 | 0 | 0 | 0 | 0 | 0 | 0 | 0 | 0 | 0 | 0 | 0 | 0 | 3 | 0 |
| Corynebacterium_1 | 0 | 0 | 0 | 0 | 0 | 0 | 0 | 0 | 0 | 0 | 0 | 2 | 0 | 0 | 0 | 0 |
| Crocinitomix | 0 | 2 | 1 | 2 | 0 | 1 | 3 | 0 | 3 | 2 | 0 | 1 | 0 | 0 | 1 | 9 |
| Calothrix_KVSF5 | 45 | 1 | 0 | 16 | 5 | 1 | 15 | 4 | 0 | 0 | 0 | 0 | 0 | 0 | 3 | 2 |
| Cnuella | 0 | 0 | 0 | 0 | 0 | 0 | 0 | 0 | 0 | 0 | 0 | 2 | 0 | 0 | 0 | 0 |
| Comamonas | 0 | 0 | 113 | 0 | 1 | 2 | 0 | 0 | 0 | 0 | 0 | 0 | 0 | 0 | 0 | 0 |
| Clostridium_sensu_stricto_10 | 7 | 9 | 3 | 0 | 2 | 14 | 6 | 2 | 2 | 1 | 13 | 4 | 23 | 0 | 14 | 8 |
| Candidatus_Moduliflexus | 0 | 0 | 0 | 1 | 0 | 1 | 2 | 0 | 0 | 1 | 0 | 0 | 0 | 0 | 0 | 0 |
| Candidatus_Nitrotoga | 0 | 0 | 0 | 3 | 0 | 3 | 0 | 0 | 0 | 0 | 0 | 0 | 0 | 0 | 0 | 0 |
| Candidatus_Megaira | 1 | 0 | 3 | 2 | 4 | 18 | 4 | 0 | 2 | 4 | 0 | 0 | 4 | 0 | 0 | 2 |
| Candidatus_Finniella | 0 | 0 | 0 | 0 | 0 | 0 | 0 | 0 | 0 | 0 | 0 | 0 | 0 | 2 | 0 | 0 |
| Candidatus_Lumbricincola | 0 | 0 | 0 | 0 | 0 | 0 | 0 | 0 | 0 | 0 | 0 | 0 | 0 | 4 | 0 | 0 |
| Candidatus_Rhabdochlamydia | 0 | 1 | 0 | 0 | 2 | 0 | 17 | 7 | 3 | 0 | 7 | 2 | 0 | 3 | 0 | 0 |
| Chryseobacterium | 0 | 0 | 0 | 0 | 0 | 0 | 0 | 0 | 0 | 0 | 0 | 0 | 2 | 0 | 0 | 0 |
| Cloacibacterium | 0 | 3 | 129 | 1 | 6 | 0 | 2 | 1 | 0 | 0 | 2 | 2 | 0 | 0 | 0 | 2 |
| Chlorobium | 0 | 0 | 3 | 0 | 0 | 0 | 0 | 0 | 0 | 0 | 0 | 2 | 0 | 0 | 0 | 2 |
| Candidatus_Tenderia | 1 | 0 | 1 | 0 | 0 | 1 | 2 | 0 | 0 | 0 | 0 | 39 | 3 | 33 | 0 | 0 |
| Catellatospora | 0 | 3 | 13 | 7 | 2 | 5 | 1 | 0 | 0 | 1 | 1 | 0 | 10 | 2 | 2 | 10 |
| Desulforegula | 0 | 0 | 0 | 0 | 0 | 0 | 2 | 0 | 0 | 0 | 0 | 0 | 1 | 2 | 2 | 1 |
| Desulforhopalus | 0 | 0 | 2 | 3 | 2 | 6 | 13 | 2 | 0 | 0 | 0 | 1 | 0 | 1 | 0 | 2 |
| C39 | 0 | 3 | 3 | 0 | 0 | 2 | 0 | 6 | 0 | 0 | 5 | 27 | 2 | 0 | 0 | 0 |
| Desulfatirhabdium | 3 | 5 | 22 | 11 | 3 | 37 | 23 | 23 | 25 | 40 | 8 | 8 | 8 | 2 | 4 | 15 |
| Desulfitobacterium | 0 | 0 | 0 | 0 | 0 | 0 | 0 | 0 | 0 | 0 | 0 | 0 | 2 | 0 | 0 | 0 |
| Breznakia | 0 | 0 | 0 | 0 | 2 | 0 | 0 | 0 | 1 | 0 | 0 | 0 | 0 | 0 | 0 | 0 |
| Dolichospermum_NIES41 | 0 | 0 | 0 | 0 | 0 | 0 | 2 | 0 | 0 | 0 | 0 | 0 | 0 | 0 | 0 | 0 |
| Domibacillus | 28 | 0 | 0 | 0 | 0 | 0 | 0 | 0 | 0 | 0 | 2 | 0 | 4 | 0 | 0 | 0 |
| Diplorickettsia | 0 | 0 | 0 | 0 | 0 | 0 | 0 | 3 | 0 | 0 | 0 | 0 | 0 | 0 | 0 | 0 |
| Desulfovermiculus | 0 | 0 | 0 | 0 | 0 | 0 | 0 | 0 | 0 | 0 | 2 | 0 | 0 | 0 | 0 | 0 |
| Dielma | 0 | 0 | 0 | 8 | 0 | 0 | 2 | 2 | 0 | 0 | 0 | 0 | 0 | 0 | 0 | 0 |
| CENA518 | 0 | 0 | 0 | 0 | 0 | 0 | 3 | 0 | 0 | 0 | 0 | 0 | 0 | 0 | 0 | 0 |
| Cutibacterium | 0 | 0 | 0 | 0 | 0 | 0 | 0 | 2 | 0 | 0 | 0 | 0 | 0 | 0 | 0 | 0 |
| Cyanothece_PCC-8801 | 0 | 0 | 0 | 0 | 0 | 0 | 0 | 0 | 0 | 0 | 0 | 0 | 3 | 0 | 0 | 0 |
| Cuspidothrix_LMECYA_163 | 0 | 25 | 1 | 0 | 4 | 0 | 1 | 1 | 1 | 1 | 0 | 0 | 0 | 0 | 0 | 0 |
| Cryptosporangium | 0 | 0 | 3 | 0 | 0 | 0 | 0 | 1 | 0 | 0 | 0 | 0 | 0 | 0 | 0 | 0 |
| Cupriavidus | 0 | 0 | 1 | 0 | 0 | 2 | 4 | 0 | 0 | 0 | 0 | 0 | 0 | 0 | 0 | 0 |
| Cyanothece_PCC_7425 | 3 | 0 | 0 | 11 | 1 | 0 | 0 | 0 | 0 | 0 | 0 | 0 | 0 | 0 | 0 | 0 |
| Dehalogenimonas | 2 | 18 | 3 | 2 | 3 | 2 | 1 | 0 | 10 | 11 | 0 | 3 | 16 | 1 | 0 | 3 |
| Desertibacter | 0 | 0 | 0 | 0 | 0 | 0 | 0 | 0 | 0 | 2 | 0 | 0 | 0 | 0 | 0 | 0 |
| Dehalobacter | 0 | 1 | 0 | 0 | 0 | 3 | 0 | 0 | 0 | 0 | 0 | 0 | 0 | 0 | 0 | 1 |
| Dechlorosoma | 1 | 5 | 1 | 5 | 1 | 11 | 8 | 0 | 5 | 2 | 0 | 0 | 7 | 0 | 3 | 4 |
| CI75cm.2.12 | 3 | 0 | 1 | 6 | 3 | 15 | 4 | 29 | 38 | 26 | 75 | 39 | 13 | 46 | 3 | 35 |
| Kineosporia | 0 | 7 | 26 | 11 | 27 | 9 | 2 | 3 | 0 | 18 | 2 | 4 | 0 | 4 | 0 | 19 |
| Pectinatus | 0 | 0 | 0 | 0 | 0 | 0 | 0 | 0 | 0 | 0 | 0 | 0 | 0 | 3 | 0 | 0 |
| Anabaena_XPORK15F | 0 | 0 | 0 | 0 | 0 | 0 | 0 | 0 | 0 | 2 | 0 | 0 | 0 | 0 | 0 | 0 |
| Parafilimonas | 0 | 0 | 10 | 0 | 0 | 2 | 0 | 0 | 0 | 0 | 1 | 0 | 0 | 0 | 0 | 3 |
| Papillibacter | 1 | 3 | 0 | 0 | 0 | 0 | 0 | 0 | 0 | 0 | 0 | 0 | 0 | 0 | 1 | 0 |
| Paracoccus | 0 | 0 | 0 | 0 | 0 | 0 | 0 | 0 | 0 | 0 | 0 | 2 | 0 | 0 | 0 | 0 |
| Pelagibacterium | 0 | 0 | 0 | 0 | 1 | 3 | 0 | 2 | 0 | 4 | 0 | 0 | 0 | 0 | 0 | 0 |
| Aminobacter | 0 | 0 | 0 | 0 | 0 | 5 | 0 | 0 | 0 | 0 | 0 | 0 | 0 | 0 | 0 | 0 |
| Phormidium_IAM_M-71 | 0 | 1 | 0 | 4 | 3 | 0 | 0 | 0 | 2 | 21 | 0 | 0 | 0 | 0 | 0 | 1 |
| Ammoniphilus | 0 | 0 | 0 | 3 | 2 | 0 | 0 | 0 | 0 | 0 | 0 | 0 | 1 | 0 | 0 | 0 |
| Pelagibius | 0 | 0 | 0 | 1 | 0 | 0 | 2 | 0 | 0 | 0 | 0 | 0 | 0 | 0 | 0 | 0 |
| Peptoclostridium | 1 | 1 | 0 | 0 | 0 | 0 | 0 | 0 | 0 | 0 | 0 | 2 | 0 | 0 | 4 | 0 |
| Anaerobacterium | 1 | 0 | 0 | 0 | 0 | 0 | 0 | 0 | 0 | 0 | 0 | 0 | 4 | 0 | 0 | 0 |
| Anaerotruncus | 39 | 15 | 27 | 20 | 18 | 24 | 28 | 34 | 26 | 45 | 36 | 26 | 31 | 32 | 21 | 30 |
| Anaerosporobacter | 1 | 0 | 0 | 0 | 1 | 0 | 0 | 0 | 0 | 0 | 0 | 0 | 1 | 0 | 0 | 0 |
| Nostoc_PCC-8976 | 1 | 0 | 0 | 0 | 2 | 0 | 2 | 0 | 0 | 1 | 0 | 0 | 0 | 0 | 0 | 0 |
| Nodularia_PCC-9350 | 0 | 0 | 0 | 0 | 0 | 0 | 0 | 0 | 0 | 2 | 0 | 0 | 0 | 0 | 0 | 0 |
| Nostoc_PCC-7524 | 0 | 0 | 0 | 0 | 0 | 0 | 0 | 3 | 1 | 0 | 0 | 0 | 0 | 0 | 0 | 0 |
| Anaerosinus | 1 | 3 | 0 | 1 | 0 | 0 | 0 | 0 | 0 | 2 | 0 | 2 | 0 | 1 | 2 | 0 |
| Anaerobium | 0 | 3 | 0 | 3 | 0 | 0 | 0 | 0 | 0 | 2 | 2 | 8 | 4 | 0 | 2 | 0 |
| Paludibacterium | 0 | 2 | 0 | 0 | 0 | 0 | 0 | 0 | 0 | 0 | 0 | 0 | 0 | 0 | 0 | 0 |
| Anaerocella | 0 | 3 | 0 | 4 | 0 | 0 | 0 | 0 | 0 | 0 | 0 | 0 | 0 | 0 | 0 | 0 |
| Oscillatoria_PCC-6304 | 0 | 0 | 2 | 0 | 0 | 0 | 0 | 0 | 0 | 0 | 0 | 0 | 0 | 0 | 0 | 0 |
| Oscillibacter | 5 | 0 | 1 | 0 | 8 | 7 | 9 | 7 | 4 | 2 | 1 | 1 | 2 | 2 | 1 | 2 |
| Pseudarthrobacter | 43 | 67 | 123 | 60 | 5 | 116 | 7 | 26 | 9 | 241 | 5 | 14 | 62 | 3 | 9 | 398 |
| Pseudochrobactrum | 0 | 0 | 0 | 0 | 0 | 0 | 0 | 0 | 0 | 0 | 0 | 0 | 0 | 0 | 2 | 0 |
| Pseudanabaena_PCC-6802 | 2 | 0 | 0 | 3 | 0 | 0 | 0 | 0 | 0 | 0 | 0 | 0 | 0 | 0 | 0 | 2 |
| Proteocatella | 5 | 0 | 1 | 0 | 0 | 0 | 0 | 0 | 0 | 0 | 0 | 0 | 0 | 0 | 0 | 0 |
| Pseudanabaena_NgrPSln22 | 0 | 0 | 0 | 0 | 0 | 0 | 2 | 0 | 0 | 0 | 0 | 0 | 0 | 0 | 0 | 0 |
| Pseudoflavitalea | 0 | 0 | 4 | 1 | 9 | 0 | 0 | 0 | 0 | 0 | 0 | 0 | 0 | 0 | 0 | 0 |
| Pseudorhodobacter | 1 | 2 | 1 | 4 | 1 | 9 | 10 | 0 | 1 | 4 | 1 | 0 | 1 | 0 | 0 | 3 |
| Pseudoxanthobacter | 0 | 0 | 0 | 0 | 3 | 0 | 0 | 0 | 0 | 0 | 0 | 0 | 0 | 0 | 0 | 0 |
| Pseudohongiella | 0 | 0 | 0 | 0 | 0 | 0 | 0 | 0 | 0 | 0 | 0 | 30 | 0 | 0 | 0 | 0 |
| Pseudofulvimonas | 0 | 0 | 2 | 0 | 0 | 1 | 0 | 0 | 1 | 0 | 0 | 0 | 0 | 0 | 0 | 0 |
| Pseudogulbenkiania | 0 | 0 | 0 | 0 | 0 | 0 | 0 | 2 | 0 | 0 | 0 | 0 | 0 | 0 | 0 | 0 |
| Proteiniclasticum | 17 | 25 | 10 | 5 | 7 | 20 | 11 | 9 | 6 | 7 | 4 | 8 | 2 | 4 | 10 | 7 |
| Pleomorphomonas | 0 | 1 | 2 | 0 | 0 | 0 | 4 | 0 | 0 | 0 | 0 | 0 | 0 | 1 | 1 | 0 |
| Plesiomonas | 0 | 0 | 0 | 2 | 2 | 0 | 8 | 0 | 1 | 2 | 0 | 0 | 0 | 0 | 3 | 1 |
| Piscinibacter | 21 | 37 | 114 | 113 | 88 | 20 | 66 | 50 | 16 | 61 | 57 | 56 | 8 | 12 | 16 | 48 |
| Phycisphaera | 0 | 0 | 0 | 0 | 0 | 0 | 0 | 0 | 0 | 0 | 0 | 0 | 0 | 3 | 0 | 0 |
| Pir3_lineage | 0 | 0 | 2 | 0 | 0 | 0 | 1 | 0 | 0 | 0 | 0 | 0 | 0 | 0 | 0 | 0 |
| Polaromonas | 0 | 0 | 0 | 0 | 0 | 0 | 0 | 0 | 0 | 0 | 0 | 3 | 0 | 0 | 0 | 0 |
| Promicromonospora | 0 | 0 | 9 | 1 | 0 | 0 | 0 | 0 | 0 | 1 | 0 | 0 | 0 | 0 | 0 | 1 |
| Prosthecomicrobium | 3 | 3 | 8 | 3 | 7 | 2 | 13 | 21 | 2 | 5 | 6 | 5 | 1 | 5 | 5 | 2 |
| Potamolinea_1PC | 0 | 0 | 0 | 0 | 0 | 0 | 0 | 0 | 0 | 3 | 0 | 0 | 0 | 0 | 0 | 0 |
| Polycyclovorans | 0 | 0 | 0 | 0 | 1 | 5 | 0 | 0 | 0 | 0 | 0 | 0 | 1 | 0 | 0 | 3 |
| Polymorphobacter | 1 | 1 | 22 | 10 | 22 | 3 | 18 | 4 | 2 | 11 | 2 | 13 | 0 | 0 | 0 | 0 |
| Lysinibacillus | 6 | 0 | 0 | 1 | 0 | 2 | 3 | 0 | 0 | 0 | 0 | 3 | 9 | 0 | 5 | 4 |
| Arenibacter | 0 | 0 | 0 | 0 | 0 | 0 | 0 | 0 | 0 | 0 | 0 | 0 | 2 | 0 | 0 | 0 |
| A7P-90m | 0 | 0 | 0 | 0 | 0 | 0 | 0 | 0 | 0 | 0 | 0 | 0 | 0 | 0 | 0 | 4 |
| Longispora | 0 | 0 | 0 | 0 | 0 | 0 | 0 | 0 | 0 | 0 | 0 | 7 | 0 | 0 | 0 | 0 |
| Luteitalea | 1 | 0 | 5 | 1 | 0 | 0 | 1 | 2 | 0 | 5 | 0 | 0 | 0 | 1 | 4 | 4 |
| MD3-55 | 0 | 0 | 0 | 0 | 0 | 0 | 2 | 0 | 0 | 0 | 0 | 0 | 0 | 0 | 0 | 0 |
| Macellibacteroides | 0 | 1 | 1 | 27 | 0 | 4 | 0 | 2 | 3 | 2 | 1 | 5 | 2 | 0 | 0 | 0 |
| Mangroviflexus | 1 | 3 | 3 | 1 | 0 | 3 | 0 | 3 | 0 | 2 | 0 | 0 | 0 | 0 | 2 | 4 |
| Arcobacter | 4 | 27 | 1 | 3 | 1 | 0 | 33 | 0 | 0 | 0 | 0 | 0 | 0 | 0 | 5 | 0 |
| MIZ17 | 0 | 2 | 0 | 0 | 0 | 0 | 0 | 0 | 0 | 0 | 0 | 0 | 4 | 0 | 0 | 0 |
| MM2 | 0 | 7 | 1 | 0 | 0 | 4 | 0 | 1 | 0 | 0 | 0 | 1 | 5 | 0 | 9 | 0 |
| Arthrobacter | 1 | 0 | 0 | 0 | 0 | 0 | 0 | 0 | 0 | 5 | 0 | 0 | 0 | 0 | 4 | 7 |
| Lactivibrio | 0 | 3 | 0 | 0 | 0 | 0 | 0 | 2 | 0 | 0 | 0 | 0 | 0 | 0 | 0 | 0 |
| Lamprocystis | 0 | 0 | 2 | 0 | 2 | 0 | 0 | 1 | 0 | 0 | 0 | 0 | 0 | 0 | 0 | 0 |
| Azospira | 0 | 13 | 10 | 1 | 0 | 7 | 0 | 2 | 0 | 4 | 8 | 9 | 0 | 0 | 0 | 0 |
| Kocuria | 0 | 1 | 3 | 0 | 0 | 3 | 0 | 0 | 0 | 1 | 0 | 3 | 0 | 0 | 0 | 4 |
| Lachnoclostridium_10 | 0 | 0 | 0 | 0 | 0 | 0 | 0 | 3 | 0 | 0 | 0 | 0 | 0 | 0 | 0 | 0 |
| Aurantimicrobium | 1 | 8 | 22 | 3 | 6 | 5 | 6 | 10 | 4 | 7 | 5 | 9 | 2 | 0 | 2 | 5 |
| Lewinella | 0 | 0 | 0 | 0 | 4 | 0 | 0 | 0 | 0 | 2 | 0 | 0 | 0 | 0 | 0 | 0 |
| Limnothrix | 0 | 0 | 0 | 0 | 0 | 0 | 0 | 0 | 0 | 0 | 0 | 2 | 0 | 0 | 0 | 0 |
| Leptonema | 0 | 7 | 0 | 1 | 0 | 0 | 3 | 0 | 2 | 1 | 10 | 3 | 1 | 4 | 2 | 0 |
| Leadbetterella | 1 | 1 | 0 | 64 | 6 | 0 | 69 | 2 | 12 | 17 | 0 | 1 | 1 | 15 | 22 | 46 |
| Lentimicrobium | 0 | 11 | 2 | 1 | 0 | 0 | 0 | 0 | 0 | 0 | 1 | 0 | 0 | 0 | 0 | 0 |
| Mucilaginibacter | 0 | 0 | 2 | 0 | 0 | 0 | 0 | 0 | 0 | 0 | 0 | 0 | 0 | 0 | 0 | 0 |
| Anoxybacillus | 0 | 0 | 0 | 0 | 0 | 0 | 0 | 0 | 0 | 0 | 0 | 0 | 2 | 0 | 0 | 0 |
| Modestobacter | 0 | 0 | 0 | 0 | 0 | 0 | 0 | 0 | 0 | 0 | 0 | 0 | 0 | 0 | 0 | 3 |
| Microlunatus | 0 | 0 | 0 | 0 | 0 | 0 | 0 | 0 | 0 | 3 | 0 | 0 | 0 | 0 | 0 | 0 |
| Micromonospora | 0 | 0 | 1 | 0 | 0 | 0 | 0 | 0 | 0 | 0 | 0 | 1 | 0 | 3 | 0 | 0 |
| Nitrolancea | 0 | 0 | 0 | 0 | 0 | 0 | 0 | 0 | 0 | 0 | 0 | 2 | 0 | 0 | 0 | 0 |
| Nocardia | 0 | 5 | 0 | 1 | 0 | 0 | 0 | 0 | 0 | 0 | 0 | 0 | 0 | 0 | 0 | 1 |
| Nocardiopsis | 0 | 0 | 0 | 2 | 0 | 0 | 0 | 0 | 0 | 0 | 0 | 0 | 0 | 0 | 0 | 0 |
| Niveispirillum | 0 | 0 | 0 | 0 | 0 | 5 | 0 | 0 | 0 | 0 | 0 | 0 | 0 | 0 | 1 | 5 |
| Ancylobacter | 0 | 2 | 5 | 0 | 1 | 4 | 0 | 0 | 0 | 1 | 1 | 1 | 1 | 0 | 0 | 7 |
| Niveibacterium | 0 | 0 | 0 | 1 | 0 | 0 | 2 | 0 | 0 | 0 | 0 | 0 | 0 | 0 | 0 | 1 |
| Microcoleus_PCC-7113 | 0 | 0 | 0 | 0 | 0 | 0 | 4 | 0 | 0 | 0 | 0 | 0 | 0 | 0 | 0 | 0 |
| Aquisphaera | 0 | 0 | 0 | 1 | 1 | 3 | 0 | 2 | 0 | 1 | 0 | 0 | 0 | 0 | 0 | 0 |
| Methylobacter | 25 | 43 | 12 | 8 | 5 | 6 | 4 | 4 | 14 | 1 | 16 | 19 | 32 | 5 | 100 | 4 |
| Methanolinea | 0 | 2 | 1 | 1 | 2 | 2 | 1 | 0 | 3 | 0 | 9 | 6 | 0 | 0 | 6 | 5 |
| Meniscus | 0 | 4 | 0 | 0 | 0 | 0 | 0 | 0 | 0 | 0 | 0 | 0 | 0 | 0 | 0 | 0 |
| Mesotoga | 0 | 0 | 0 | 0 | 0 | 0 | 0 | 0 | 0 | 0 | 0 | 2 | 0 | 0 | 0 | 0 |
| Methylocella | 0 | 0 | 0 | 0 | 0 | 4 | 0 | 0 | 0 | 0 | 0 | 0 | 0 | 0 | 0 | 0 |
| Microbacterium | 0 | 3 | 8 | 0 | 3 | 2 | 0 | 0 | 0 | 2 | 1 | 0 | 1 | 0 | 0 | 3 |
| Microcoleus_Es-Yyy1400 | 0 | 0 | 0 | 0 | 0 | 0 | 2 | 0 | 0 | 0 | 0 | 0 | 0 | 0 | 0 | 0 |
| Aquimonas | 0 | 0 | 9 | 0 | 0 | 3 | 0 | 0 | 0 | 7 | 1 | 2 | 0 | 0 | 0 | 3 |
| Methylophilus | 0 | 16 | 23 | 6 | 2 | 6 | 4 | 4 | 4 | 6 | 11 | 2 | 0 | 0 | 1 | 0 |
| Methylosarcina | 0 | 0 | 0 | 2 | 0 | 3 | 10 | 1 | 0 | 0 | 2 | 0 | 0 | 0 | 0 | 0 |

Table S4. fungal genus in surface sediments of Erhai Lake

| Genus | BP | DZ | FMY | KLC | LHQ | LQ | RLYC | SPC | THC | TY | XC | XHC | XJY | XYZ | YHY | YJC |
| --- | --- | --- | --- | --- | --- | --- | --- | --- | --- | --- | --- | --- | --- | --- | --- | --- |
| Arcuospathidium | 462 | 64 | 366 | 804 | 2364 | 222 | 261 | 861 | 1007 | 3903 | 1385 | 542 | 134 | 932 | 205 | 1534 |
| Prorodon | 3351 | 421 | 1193 | 1368 | 2 | 782 | 2478 | 1559 | 1189 | 948 | 906 | 32 | 1769 | 771 | 25 | 1309 |
| Penicillium | 141 | 22 | 12 | 101 | 33 | 19 | 135 | 41 | 47 | 44 | 22 | 24 | 75 | 5 | 80 | 0 |
| Frontonia | 23 | 7572 | 12491 | 3490 | 36 | 152 | 8776 | 262 | 1886 | 3506 | 13 | 77 | 42 | 7804 | 86 | 13 |
| Vorticella | 4 | 443 | 119 | 39 | 256 | 8 | 579 | 74 | 1411 | 991 | 19 | 6 | 7 | 156 | 52 | 79 |
| Mortierella | 9 | 148 | 0 | 83 | 0 | 7 | 49 | 2 | 48 | 59 | 0 | 11 | 112 | 72 | 0 | 0 |
| Cyrtohymena | 0 | 135 | 7 | 113 | 0 | 38 | 369 | 431 | 491 | 44 | 0 | 2 | 2 | 62 | 300 | 2162 |
| Navicula | 183 | 3 | 11 | 5 | 0 | 1178 | 56 | 17 | 10 | 0 | 4 | 18 | 126 | 146 | 314 | 776 |
| Acremonium | 27 | 60 | 4 | 984 | 32 | 121 | 202 | 159 | 1008 | 79 | 36 | 43 | 101 | 273 | 49 | 105 |
| Talaromyces | 36 | 5 | 0 | 13 | 26 | 16 | 21 | 14 | 24 | 45 | 9 | 0 | 8 | 20 | 32 | 15 |
| Gomphonema | 10 | 0 | 0 | 3 | 9 | 61 | 0 | 79 | 278 | 61 | 0 | 0 | 11 | 0 | 11 | 24 |
| Coprinellus | 27 | 142 | 0 | 129 | 0 | 9 | 5 | 17 | 28 | 0 | 0 | 0 | 57 | 15 | 57 | 0 |
| Spumella | 13 | 19 | 2 | 13 | 0 | 0 | 16 | 5 | 14 | 0 | 0 | 0 | 14 | 15 | 9 | 0 |
| Podospora | 0 | 0 | 296 | 294 | 0 | 0 | 66 | 1384 | 132 | 3 | 0 | 0 | 96 | 738 | 0 | 0 |
| Epistylis | 100 | 358 | 0 | 0 | 1271 | 0 | 2 | 5 | 8 | 5 | 120 | 0 | 0 | 4 | 1169 | 32 |
| Trichoderma | 67 | 28 | 7 | 16 | 20 | 38 | 31 | 62 | 26 | 43 | 0 | 0 | 32 | 153 | 3 | 0 |
| Fusarium | 4 | 0 | 27 | 800 | 65 | 2 | 22 | 0 | 30 | 2 | 46 | 12 | 82 | 59 | 10 | 33 |
| Cladosporium | 88 | 87 | 53 | 191 | 134 | 51 | 120 | 478 | 164 | 123 | 4 | 8 | 88 | 66 | 388 | 73 |
| Inocybe | 0 | 20 | 0 | 384 | 0 | 0 | 48 | 0 | 0 | 0 | 0 | 73 | 0 | 0 | 0 | 15 |
| Hygrocybe | 0 | 0 | 0 | 0 | 0 | 0 | 0 | 7 | 0 | 0 | 0 | 0 | 10 | 0 | 91 | 0 |
| Pseudeurotium | 221 | 48 | 17 | 101 | 322 | 37 | 48 | 718 | 144 | 308 | 14 | 28 | 86 | 13 | 103 | 81 |
| Aspergillus | 34 | 3 | 0 | 0 | 0 | 14 | 0 | 6 | 4 | 19 | 2 | 93 | 14 | 0 | 66 | 10 |
| Halteria | 0 | 13 | 3 | 172 | 0 | 27 | 15 | 9 | 17 | 167 | 0 | 13 | 29 | 140 | 2 | 3 |
| Zopfiella | 8 | 0 | 47 | 0 | 0 | 15 | 32 | 80 | 261 | 11 | 0 | 0 | 127 | 583 | 79 | 226 |
| Betamyces | 278 | 73 | 114 | 56 | 0 | 48 | 189 | 0 | 5 | 10 | 0 | 46 | 30 | 49 | 70 | 21 |
| Ichthyophthirius | 0 | 128 | 193 | 4 | 0 | 0 | 30 | 0 | 3 | 3 | 0 | 0 | 0 | 149 | 11 | 0 |
| Zygorhizidium | 11 | 0 | 0 | 21 | 38 | 8 | 53 | 0 | 0 | 27 | 6 | 0 | 36 | 0 | 14 | 48 |
| Pholiota | 13 | 111 | 11 | 87 | 0 | 0 | 5 | 0 | 2 | 17 | 0 | 0 | 8 | 0 | 25 | 0 |
| Chaetomium | 0 | 6 | 6 | 0 | 0 | 14 | 0 | 28 | 26 | 0 | 0 | 0 | 20 | 0 | 0 | 0 |
| Periconia | 0 | 0 | 9 | 0 | 0 | 0 | 14 | 37 | 0 | 13 | 0 | 3 | 33 | 10 | 0 | 0 |
| Tetracladium | 6 | 5 | 0 | 78 | 14 | 0 | 0 | 0 | 2 | 0 | 9 | 0 | 4 | 0 | 0 | 0 |
| Serendipita | 4 | 185 | 0 | 22 | 0 | 4 | 24 | 0 | 2 | 0 | 3 | 0 | 0 | 0 | 2 | 0 |
| Metarhizium | 0 | 0 | 10 | 9 | 0 | 4 | 8 | 0 | 15 | 7 | 0 | 2 | 25 | 4 | 0 | 0 |
| Paramecium | 0 | 221 | 382 | 0 | 0 | 0 | 7 | 38 | 0 | 3 | 6 | 12 | 0 | 51 | 69 | 0 |
| Gibellulopsis | 28 | 0 | 5 | 120 | 0 | 161 | 121 | 22 | 1660 | 185 | 0 | 0 | 92 | 66 | 0 | 25 |
| Westerdykella | 120 | 0 | 0 | 0 | 0 | 30 | 0 | 41 | 27 | 8 | 0 | 0 | 9 | 74 | 31 | 0 |
| Vorticellides | 4 | 0 | 26 | 4 | 0 | 285 | 6 | 3 | 0 | 14 | 15 | 0 | 230 | 56 | 5 | 0 |
| Cyclotella | 0 | 0 | 2 | 2 | 0 | 2 | 2 | 6 | 5 | 4 | 0 | 0 | 0 | 0 | 0 | 2 |
| Coprinopsis | 0 | 0 | 0 | 0 | 0 | 8 | 0 | 18 | 5 | 0 | 2 | 0 | 9 | 14 | 29 | 0 |
| Tomentella | 44 | 38 | 0 | 193 | 8 | 6 | 80 | 14 | 6 | 0 | 0 | 0 | 41 | 11 | 0 | 0 |
| Funneliformis | 0 | 0 | 4 | 188 | 0 | 0 | 0 | 0 | 0 | 0 | 0 | 0 | 0 | 0 | 0 | 0 |
| Geminibasidium | 0 | 0 | 0 | 0 | 0 | 0 | 0 | 0 | 3 | 0 | 0 | 0 | 0 | 0 | 305 | 0 |
| Plectosphaerella | 20 | 6 | 8 | 81 | 39 | 8 | 15 | 48 | 19 | 49 | 17 | 26 | 72 | 64 | 0 | 37 |
| Amphileptus | 0 | 5 | 32 | 0 | 0 | 0 | 49 | 0 | 0 | 0 | 35 | 0 | 25 | 4 | 61 | 0 |
| Coniochaeta | 17 | 0 | 9 | 0 | 0 | 6 | 4 | 8 | 8 | 0 | 0 | 0 | 18 | 8 | 0 | 60 |
| Exophiala | 0 | 0 | 2 | 6 | 0 | 0 | 5 | 0 | 0 | 0 | 0 | 0 | 9 | 2 | 4 | 0 |
| Kazachstania | 0 | 4 | 0 | 0 | 0 | 26 | 12 | 24 | 6 | 2 | 9 | 0 | 35 | 0 | 0 | 0 |
| Spirostomum | 10 | 88 | 134 | 0 | 0 | 0 | 107 | 0 | 2 | 6 | 0 | 0 | 0 | 25 | 10 | 0 |
| Acrobeloides | 0 | 2 | 0 | 0 | 3 | 0 | 0 | 0 | 29 | 0 | 0 | 0 | 0 | 739 | 0 | 0 |
| Neobulgaria | 7 | 22 | 0 | 0 | 0 | 0 | 12 | 0 | 12 | 0 | 0 | 0 | 43 | 0 | 7 | 0 |
| Askenasia | 4 | 4 | 0 | 3 | 0 | 396 | 0 | 2 | 3 | 641 | 0 | 0 | 0 | 0 | 0 | 0 |
| Cirrenalia | 2 | 9 | 3 | 31 | 7 | 0 | 74 | 8 | 19 | 4 | 9 | 0 | 3 | 111 | 16 | 0 |
| Preussia | 10 | 0 | 0 | 176 | 0 | 0 | 3 | 14 | 0 | 0 | 0 | 0 | 9 | 0 | 14 | 0 |
| Psathyrella | 0 | 0 | 0 | 0 | 0 | 0 | 9 | 5 | 0 | 0 | 11 | 0 | 22 | 0 | 11 | 0 |
| Emericellopsis | 189 | 143 | 208 | 541 | 122 | 247 | 175 | 431 | 143 | 230 | 338 | 24 | 212 | 454 | 5747 | 280 |
| Trachelophyllum | 0 | 0 | 0 | 0 | 0 | 0 | 0 | 0 | 49 | 0 | 0 | 0 | 0 | 274 | 0 | 0 |
| Macrocyclops | 0 | 0 | 88 | 0 | 0 | 0 | 394 | 0 | 4 | 9 | 0 | 0 | 0 | 111 | 0 | 0 |
| Pichia | 2 | 0 | 4 | 0 | 5 | 14 | 0 | 25 | 8 | 0 | 0 | 0 | 0 | 0 | 0 | 0 |
| Ochroconis | 5 | 45 | 0 | 154 | 2 | 0 | 14 | 0 | 21 | 0 | 0 | 0 | 20 | 2 | 0 | 0 |
| Pyrenochaetopsis | 16 | 15 | 0 | 41 | 0 | 18 | 28 | 12 | 0 | 45 | 0 | 0 | 21 | 0 | 0 | 0 |
| Pyrenochaeta | 14 | 5 | 0 | 4 | 0 | 4 | 0 | 0 | 0 | 15 | 8 | 0 | 16 | 0 | 0 | 0 |
| Semispathidium | 0 | 0 | 0 | 17 | 0 | 0 | 209 | 0 | 42 | 0 | 0 | 0 | 0 | 233 | 2 | 0 |
| Scutellinia | 102 | 0 | 41 | 0 | 0 | 181 | 10 | 5 | 139 | 53 | 134 | 0 | 50 | 54 | 45 | 0 |
| Clonostachys | 0 | 0 | 0 | 43 | 0 | 0 | 6 | 0 | 7 | 9 | 0 | 0 | 5 | 7 | 0 | 0 |
| Vishniacozyma | 0 | 0 | 0 | 12 | 0 | 0 | 0 | 12 | 0 | 26 | 0 | 0 | 9 | 0 | 0 | 0 |
| Anteholosticha | 0 | 68 | 13 | 0 | 0 | 0 | 27 | 2 | 0 | 0 | 4 | 0 | 0 | 53 | 0 | 0 |
| Myrmecridium | 3 | 0 | 0 | 0 | 18 | 0 | 0 | 3 | 30 | 0 | 0 | 0 | 7 | 6 | 0 | 0 |
| Tintinnidium | 0 | 0 | 0 | 0 | 0 | 4 | 2 | 0 | 14 | 75 | 0 | 0 | 0 | 0 | 0 | 0 |
| Coprinus | 0 | 0 | 0 | 14 | 0 | 0 | 29 | 0 | 7 | 0 | 0 | 0 | 0 | 0 | 9 | 0 |
| Lophiostoma | 4 | 2 | 0 | 9 | 0 | 0 | 0 | 0 | 0 | 0 | 0 | 0 | 6 | 0 | 4 | 0 |
| Strongylidium | 42 | 2 | 0 | 202 | 0 | 0 | 557 | 220 | 0 | 0 | 0 | 0 | 0 | 38 | 309 | 0 |
| Scleroderma | 0 | 0 | 0 | 0 | 0 | 0 | 9 | 3 | 0 | 0 | 0 | 0 | 3 | 0 | 27 | 0 |
| Pseudocoleophoma | 0 | 0 | 0 | 25 | 32 | 6 | 0 | 0 | 0 | 0 | 0 | 11 | 10 | 0 | 0 | 0 |
| Gonyostomum | 0 | 0 | 0 | 0 | 0 | 0 | 5 | 0 | 0 | 88 | 0 | 0 | 0 | 0 | 0 | 0 |
| Phlyctochytrium | 0 | 9 | 0 | 0 | 0 | 18 | 79 | 0 | 0 | 18 | 0 | 0 | 27 | 0 | 0 | 0 |
| Mesocyclops | 0 | 4 | 0 | 0 | 0 | 0 | 0 | 0 | 190 | 0 | 6 | 0 | 0 | 4 | 233 | 0 |
| Mucor | 6 | 9 | 0 | 4 | 61 | 0 | 0 | 0 | 0 | 0 | 0 | 2 | 0 | 0 | 3 | 0 |
| Agaricus | 0 | 0 | 0 | 0 | 0 | 0 | 0 | 7 | 4 | 0 | 0 | 0 | 9 | 0 | 0 | 2 |
| Candida | 20 | 11 | 7 | 23 | 214 | 47 | 19 | 48 | 13 | 0 | 51 | 0 | 17 | 28 | 38 | 0 |
| Sellaphora | 6 | 0 | 0 | 0 | 0 | 62 | 6 | 3 | 0 | 33 | 0 | 0 | 22 | 0 | 0 | 0 |
| Beauveria | 0 | 88 | 0 | 18 | 0 | 0 | 9 | 8 | 30 | 0 | 0 | 0 | 12 | 46 | 0 | 0 |
| Phallus | 34 | 0 | 0 | 4 | 18 | 0 | 6 | 0 | 4 | 7 | 0 | 0 | 0 | 0 | 15 | 0 |
| Hypochnicium | 83 | 111 | 0 | 63 | 2 | 0 | 141 | 0 | 0 | 8 | 7 | 0 | 23 | 29 | 22 | 104 |
| Sarocladium | 9 | 0 | 0 | 0 | 0 | 0 | 17 | 0 | 0 | 0 | 0 | 0 | 0 | 5 | 0 | 0 |
| Synura | 0 | 0 | 0 | 0 | 0 | 0 | 5 | 11 | 3 | 0 | 0 | 0 | 0 | 0 | 0 | 0 |
| Paracremonium | 60 | 2 | 2 | 50 | 0 | 0 | 16 | 0 | 71 | 0 | 6 | 0 | 30 | 0 | 0 | 0 |
| Cystobasidium | 0 | 0 | 0 | 6 | 0 | 0 | 0 | 6 | 0 | 0 | 0 | 0 | 2 | 0 | 4 | 0 |
| Entophlyctis | 0 | 81 | 3 | 0 | 18 | 0 | 77 | 0 | 0 | 5 | 0 | 0 | 0 | 0 | 2 | 0 |
| Thelonectria | 0 | 16 | 0 | 0 | 0 | 0 | 11 | 0 | 0 | 0 | 0 | 0 | 5 | 0 | 10 | 0 |
| Pseudotontonia | 0 | 3 | 28 | 0 | 0 | 45 | 40 | 0 | 0 | 0 | 357 | 0 | 0 | 59 | 688 | 0 |
| Homalogastra | 0 | 0 | 0 | 20 | 0 | 0 | 158 | 0 | 0 | 0 | 0 | 0 | 0 | 0 | 0 | 53 |
| Saitozyma | 34 | 0 | 15 | 0 | 7 | 0 | 37 | 17 | 60 | 0 | 0 | 0 | 16 | 0 | 316 | 16 |
| Spongospora | 5 | 0 | 0 | 0 | 0 | 26 | 0 | 0 | 0 | 6 | 0 | 0 | 10 | 0 | 0 | 0 |
| Paraphaeosphaeria | 43 | 0 | 0 | 119 | 107 | 0 | 47 | 7 | 52 | 28 | 40 | 0 | 0 | 5 | 0 | 0 |
| Malassezia | 4 | 11 | 0 | 0 | 0 | 0 | 2 | 0 | 0 | 0 | 6 | 20 | 0 | 0 | 0 | 0 |
| Ascobolus | 5 | 0 | 15 | 0 | 0 | 3 | 365 | 0 | 0 | 0 | 61 | 0 | 11 | 0 | 0 | 0 |
| Melanopsichium | 0 | 2 | 0 | 0 | 0 | 0 | 0 | 4 | 3 | 2 | 0 | 0 | 0 | 0 | 0 | 0 |
| Sebacina | 0 | 2 | 0 | 0 | 0 | 15 | 0 | 0 | 0 | 0 | 0 | 0 | 3 | 0 | 4 | 0 |
| Paraphoma | 0 | 9 | 0 | 0 | 0 | 0 | 0 | 0 | 0 | 0 | 0 | 0 | 10 | 0 | 10 | 0 |
| Scedosporium | 38 | 0 | 2 | 0 | 7 | 0 | 22 | 36 | 12 | 29 | 0 | 0 | 0 | 0 | 50 | 0 |
| Geopora | 4 | 0 | 0 | 0 | 0 | 0 | 0 | 7 | 0 | 0 | 0 | 23 | 5 | 0 | 0 | 0 |
| Aphanoascus | 3 | 0 | 0 | 25 | 19 | 0 | 3 | 0 | 15 | 0 | 0 | 0 | 35 | 0 | 0 | 0 |
| Solicoccozyma | 0 | 0 | 0 | 266 | 0 | 0 | 0 | 0 | 3 | 0 | 0 | 0 | 25 | 0 | 5 | 0 |
| Saccharomyces | 0 | 0 | 0 | 24 | 0 | 8 | 0 | 0 | 4 | 32 | 0 | 0 | 5 | 0 | 3 | 2 |
| Furculomyces | 0 | 18 | 2 | 20 | 0 | 0 | 2 | 0 | 0 | 0 | 20 | 4 | 4 | 0 | 0 | 0 |
| Entoloma | 0 | 11 | 0 | 7 | 0 | 0 | 0 | 0 | 0 | 0 | 0 | 0 | 6 | 0 | 0 | 25 |
| Enchelyodon | 0 | 0 | 0 | 0 | 0 | 0 | 0 | 0 | 549 | 0 | 0 | 0 | 0 | 0 | 0 | 0 |
| Stylonychia | 0 | 51 | 6 | 0 | 0 | 2 | 5 | 0 | 0 | 0 | 0 | 0 | 0 | 0 | 0 | 0 |
| Spathidium | 213 | 0 | 0 | 0 | 0 | 0 | 4 | 2 | 0 | 0 | 0 | 0 | 0 | 397 | 0 | 0 |
| Jahnula | 0 | 10 | 11 | 0 | 0 | 0 | 0 | 0 | 0 | 0 | 0 | 0 | 0 | 5 | 0 | 0 |
| Zoothamnium | 0 | 0 | 0 | 0 | 0 | 9 | 183 | 0 | 3 | 0 | 0 | 3 | 0 | 0 | 14 | 0 |
| Abortiporus | 204 | 6 | 43 | 110 | 244 | 32 | 323 | 430 | 47 | 110 | 42 | 40 | 15 | 136 | 18 | 294 |
| Holosticha | 0 | 0 | 0 | 0 | 0 | 196 | 0 | 0 | 0 | 0 | 18 | 3 | 0 | 2 | 7 | 0 |
| Gonostomum | 0 | 0 | 0 | 4 | 0 | 0 | 57 | 0 | 0 | 4 | 0 | 0 | 0 | 0 | 0 | 0 |
| Scytalidium | 0 | 0 | 0 | 0 | 0 | 0 | 16 | 0 | 0 | 0 | 0 | 0 | 0 | 0 | 10 | 0 |
| Strombidium | 0 | 0 | 0 | 102 | 0 | 53 | 63 | 0 | 0 | 0 | 53 | 2 | 87 | 9 | 0 | 0 |
| Epicarchesium | 0 | 0 | 4 | 0 | 0 | 34 | 0 | 0 | 0 | 0 | 3 | 0 | 0 | 0 | 0 | 0 |
| Gamsia | 4 | 3 | 0 | 9 | 0 | 5 | 25 | 0 | 12 | 0 | 0 | 0 | 103 | 0 | 43 | 0 |
| Fuscheria | 0 | 0 | 0 | 25 | 0 | 0 | 0 | 0 | 25 | 521 | 0 | 0 | 0 | 0 | 0 | 0 |
| Anguillospora | 13 | 15 | 7 | 18 | 0 | 0 | 13 | 0 | 0 | 0 | 0 | 0 | 8 | 9 | 14 | 0 |
| Purpureocillium | 0 | 4 | 0 | 7 | 0 | 0 | 5 | 0 | 0 | 0 | 0 | 0 | 0 | 0 | 0 | 0 |
| Cladorrhinum | 5 | 0 | 53 | 0 | 0 | 0 | 63 | 190 | 88 | 15 | 0 | 0 | 88 | 220 | 0 | 0 |
| Oidiodendron | 0 | 3 | 0 | 0 | 0 | 0 | 0 | 0 | 0 | 0 | 0 | 0 | 3 | 0 | 13 | 0 |
| Microascus | 0 | 0 | 0 | 11 | 0 | 0 | 0 | 0 | 15 | 0 | 0 | 0 | 9 | 0 | 0 | 0 |
| Massarina | 0 | 21 | 19 | 457 | 35 | 15 | 7 | 26 | 83 | 5 | 11 | 0 | 11 | 24 | 401 | 12 |
| Arthrobotrys | 0 | 0 | 0 | 0 | 21 | 0 | 0 | 0 | 4 | 0 | 7 | 0 | 0 | 0 | 0 | 0 |
| Wallemia | 0 | 0 | 0 | 0 | 0 | 0 | 0 | 10 | 0 | 0 | 0 | 0 | 0 | 0 | 13 | 0 |
| Volutella | 0 | 0 | 0 | 11 | 0 | 0 | 0 | 5 | 0 | 0 | 0 | 0 | 20 | 0 | 0 | 0 |
| Magnaporthe | 0 | 0 | 0 | 20 | 0 | 0 | 0 | 0 | 0 | 0 | 0 | 0 | 8 | 0 | 0 | 0 |
| Vannella | 0 | 0 | 2 | 0 | 0 | 0 | 0 | 0 | 5 | 0 | 0 | 0 | 7 | 0 | 0 | 0 |
| Phoma | 5 | 0 | 0 | 8 | 10 | 0 | 0 | 38 | 0 | 0 | 0 | 0 | 0 | 0 | 0 | 0 |
| Byssothecium | 0 | 0 | 0 | 0 | 0 | 0 | 0 | 0 | 0 | 0 | 0 | 0 | 18 | 8 | 0 | 0 |
| Cadophora | 6 | 0 | 0 | 68 | 0 | 0 | 0 | 0 | 75 | 0 | 0 | 0 | 8 | 0 | 0 | 0 |
| Chaetosphaeria | 0 | 0 | 0 | 0 | 0 | 0 | 0 | 0 | 2 | 0 | 0 | 0 | 12 | 0 | 0 | 0 |
| Trematosphaeria | 131 | 98 | 48 | 71 | 36 | 33 | 71 | 70 | 72 | 39 | 65 | 63 | 228 | 231 | 94 | 72 |
| Auxarthron | 0 | 0 | 0 | 12 | 0 | 0 | 0 | 0 | 0 | 0 | 0 | 0 | 49 | 0 | 14 | 0 |
| Trichothecium | 0 | 12 | 2 | 56 | 0 | 0 | 0 | 0 | 10 | 0 | 0 | 0 | 4 | 257 | 2 | 0 |
| Cytospora | 0 | 0 | 0 | 0 | 0 | 0 | 0 | 14 | 5 | 3 | 0 | 0 | 0 | 0 | 0 | 0 |
| Dactylonectria | 0 | 3 | 0 | 19 | 0 | 0 | 0 | 0 | 0 | 0 | 9 | 0 | 0 | 0 | 0 | 0 |
| Aquamyces | 0 | 0 | 10 | 0 | 0 | 0 | 0 | 0 | 0 | 0 | 2 | 0 | 5 | 19 | 0 | 0 |
| Ustilago | 0 | 0 | 2 | 7 | 0 | 0 | 0 | 12 | 9 | 0 | 0 | 30 | 0 | 4 | 0 | 0 |
| Tetrahymena | 0 | 0 | 21 | 0 | 0 | 0 | 0 | 83 | 0 | 0 | 21 | 0 | 0 | 0 | 4 | 0 |
| Leohumicola | 0 | 0 | 0 | 0 | 0 | 0 | 0 | 0 | 0 | 0 | 0 | 0 | 10 | 3 | 2 | 0 |
| Barnettozyma | 0 | 0 | 0 | 0 | 0 | 0 | 15 | 0 | 0 | 0 | 0 | 0 | 10 | 0 | 0 | 0 |
| Minutisphaera | 0 | 25 | 0 | 74 | 3 | 0 | 14 | 0 | 0 | 0 | 0 | 0 | 0 | 0 | 0 | 0 |
| Leptodiscella | 0 | 0 | 0 | 84 | 0 | 0 | 4 | 20 | 20 | 6 | 0 | 0 | 14 | 0 | 27 | 0 |
| Rimostrombidium | 0 | 2 | 0 | 0 | 238 | 0 | 44 | 2 | 0 | 2 | 0 | 0 | 0 | 0 | 0 | 0 |
| Cyclidium | 0 | 0 | 3 | 0 | 11 | 0 | 0 | 0 | 0 | 0 | 5 | 0 | 0 | 0 | 35 | 0 |
| Botryotrichum | 35 | 0 | 0 | 0 | 0 | 0 | 0 | 0 | 14 | 0 | 0 | 0 | 77 | 0 | 0 | 0 |
| Bothrioneurum | 0 | 96 | 0 | 0 | 0 | 0 | 0 | 0 | 0 | 0 | 159 | 0 | 0 | 0 | 0 | 0 |
| Bifidochaetus | 3 | 2 | 0 | 0 | 0 | 0 | 0 | 0 | 8 | 0 | 0 | 0 | 16 | 0 | 0 | 0 |
| Sakaguchia | 0 | 0 | 0 | 0 | 0 | 0 | 3 | 0 | 3 | 0 | 0 | 0 | 3 | 0 | 0 | 0 |
| Idriella | 0 | 0 | 0 | 0 | 0 | 0 | 6 | 0 | 12 | 0 | 0 | 0 | 0 | 0 | 0 | 0 |
| Papiliotrema | 0 | 0 | 0 | 20 | 0 | 0 | 0 | 0 | 0 | 0 | 0 | 0 | 7 | 0 | 0 | 0 |
| Hypholoma | 0 | 0 | 0 | 0 | 0 | 0 | 0 | 14 | 4 | 0 | 5 | 0 | 0 | 0 | 0 | 0 |
| Nais | 0 | 5 | 3 | 0 | 0 | 0 | 0 | 0 | 0 | 0 | 0 | 0 | 0 | 0 | 0 | 0 |
| Nectriopsis | 0 | 0 | 0 | 31 | 0 | 12 | 0 | 0 | 0 | 19 | 0 | 0 | 7 | 3 | 0 | 0 |
| Microdochium | 0 | 0 | 0 | 4 | 0 | 0 | 0 | 0 | 0 | 22 | 5 | 0 | 0 | 0 | 0 | 0 |
| Nectria | 0 | 0 | 0 | 0 | 0 | 0 | 0 | 0 | 0 | 0 | 0 | 0 | 17 | 0 | 0 | 0 |
| Hysterangium | 0 | 0 | 0 | 0 | 0 | 26 | 0 | 0 | 0 | 0 | 0 | 0 | 0 | 0 | 9 | 0 |
| Issatchenkia | 0 | 0 | 0 | 0 | 54 | 4 | 0 | 5 | 0 | 0 | 0 | 0 | 0 | 0 | 0 | 0 |
| Lobulomyces | 19 | 0 | 67 | 22 | 0 | 7 | 0 | 0 | 0 | 4 | 0 | 0 | 19 | 0 | 0 | 0 |
| Scopuloides | 0 | 0 | 0 | 0 | 0 | 0 | 0 | 0 | 0 | 0 | 0 | 0 | 0 | 4 | 5 | 0 |
| Lophiotrema | 0 | 0 | 0 | 0 | 4 | 0 | 0 | 0 | 0 | 17 | 0 | 0 | 6 | 0 | 13 | 0 |
| Monoblepharella | 0 | 0 | 4 | 2 | 0 | 0 | 0 | 16 | 0 | 0 | 0 | 0 | 0 | 0 | 0 | 0 |
| Lentithecium | 79 | 0 | 0 | 141 | 0 | 0 | 9 | 0 | 28 | 0 | 0 | 0 | 0 | 11 | 5 | 0 |
| Schizothecium | 0 | 0 | 0 | 0 | 0 | 0 | 0 | 0 | 0 | 0 | 0 | 0 | 4 | 4 | 0 | 0 |
| Olpidium | 0 | 2 | 0 | 0 | 0 | 0 | 0 | 0 | 0 | 0 | 0 | 0 | 0 | 0 | 5 | 0 |
| Saturnispora | 13 | 0 | 0 | 4 | 0 | 0 | 0 | 0 | 0 | 0 | 0 | 0 | 0 | 0 | 0 | 0 |
| Naganishia | 11 | 0 | 0 | 0 | 0 | 0 | 9 | 6 | 0 | 0 | 0 | 0 | 0 | 0 | 0 | 0 |
| Mastigobasidium | 0 | 11 | 0 | 0 | 0 | 0 | 0 | 0 | 0 | 0 | 0 | 0 | 0 | 0 | 0 | 0 |
| Roussoella | 0 | 0 | 0 | 0 | 0 | 0 | 5 | 0 | 0 | 0 | 0 | 0 | 6 | 0 | 0 | 0 |
| Oedogoniomyces | 0 | 0 | 0 | 15 | 0 | 0 | 0 | 2 | 0 | 0 | 0 | 0 | 0 | 0 | 0 | 0 |
| Mallomonas | 0 | 0 | 0 | 0 | 0 | 0 | 7 | 0 | 0 | 46 | 0 | 0 | 0 | 0 | 0 | 0 |
| Rhodotorula | 3 | 0 | 0 | 0 | 0 | 0 | 0 | 0 | 10 | 0 | 0 | 0 | 0 | 0 | 0 | 0 |
| Ogataea | 0 | 5 | 0 | 0 | 0 | 0 | 2 | 0 | 0 | 0 | 0 | 0 | 0 | 0 | 0 | 0 |
| Knufia | 0 | 14 | 0 | 0 | 0 | 0 | 0 | 0 | 0 | 0 | 0 | 0 | 3 | 0 | 0 | 0 |
| Cephalotrichum | 0 | 0 | 0 | 0 | 0 | 0 | 0 | 8 | 4 | 0 | 0 | 0 | 3 | 0 | 0 | 0 |
| Trechispora | 0 | 0 | 0 | 0 | 0 | 0 | 4 | 0 | 0 | 0 | 0 | 0 | 29 | 0 | 0 | 0 |
| Phialophora | 0 | 0 | 0 | 0 | 0 | 21 | 0 | 0 | 0 | 0 | 0 | 0 | 0 | 0 | 4 | 0 |
| Trichomerium | 5 | 0 | 0 | 0 | 0 | 0 | 0 | 0 | 0 | 0 | 6 | 0 | 0 | 0 | 0 | 0 |
| Protrudomyces | 4 | 0 | 0 | 0 | 0 | 4 | 0 | 0 | 0 | 0 | 0 | 0 | 0 | 0 | 0 | 0 |
| Tulostoma | 0 | 0 | 0 | 0 | 0 | 0 | 0 | 0 | 0 | 0 | 0 | 0 | 5 | 0 | 7 | 0 |
| Carchesium | 0 | 9 | 0 | 0 | 0 | 0 | 0 | 0 | 0 | 0 | 0 | 6 | 0 | 0 | 32 | 0 |
| Chrysosporium | 0 | 10 | 8 | 0 | 0 | 0 | 0 | 0 | 0 | 0 | 0 | 6 | 10 | 0 | 19 | 0 |
| Conocybe | 0 | 0 | 0 | 0 | 0 | 0 | 0 | 0 | 7 | 0 | 3 | 0 | 0 | 0 | 0 | 0 |
| Corticium | 0 | 0 | 0 | 112 | 0 | 0 | 0 | 0 | 0 | 0 | 0 | 0 | 14 | 0 | 4 | 0 |
| Cortinarius | 0 | 0 | 3 | 0 | 0 | 0 | 0 | 0 | 0 | 0 | 0 | 0 | 13 | 0 | 0 | 0 |
| Phialocephala | 0 | 0 | 0 | 0 | 0 | 0 | 0 | 0 | 33 | 0 | 0 | 0 | 6 | 15 | 0 | 62 |
| Cistella | 0 | 0 | 0 | 0 | 0 | 0 | 0 | 0 | 0 | 13 | 0 | 0 | 8 | 0 | 0 | 0 |
| Clavaria | 0 | 0 | 0 | 0 | 0 | 0 | 0 | 0 | 0 | 0 | 0 | 0 | 10 | 0 | 4 | 0 |
| Torulaspora | 0 | 0 | 0 | 0 | 0 | 0 | 0 | 0 | 0 | 0 | 0 | 0 | 5 | 0 | 5 | 0 |
| Zygosaccharomyces | 0 | 0 | 0 | 0 | 6 | 0 | 0 | 0 | 0 | 0 | 0 | 0 | 0 | 0 | 0 | 5 |
| Alfaria | 0 | 0 | 0 | 0 | 0 | 0 | 0 | 0 | 0 | 0 | 0 | 15 | 0 | 0 | 0 | 51 |
| Alternaria | 0 | 0 | 0 | 0 | 0 | 0 | 0 | 0 | 0 | 0 | 0 | 0 | 6 | 2 | 0 | 0 |
| Acrostalagmus | 27 | 0 | 0 | 0 | 0 | 0 | 29 | 6 | 5 | 16 | 11 | 0 | 150 | 8 | 6 | 33 |
| Acaulium | 14 | 0 | 0 | 0 | 0 | 0 | 4 | 0 | 0 | 0 | 0 | 0 | 0 | 0 | 0 | 0 |
| Acicuseptoria | 6 | 0 | 0 | 0 | 0 | 0 | 6 | 9 | 0 | 0 | 0 | 0 | 0 | 0 | 0 | 0 |
| Acrocalymma | 6 | 0 | 0 | 113 | 0 | 0 | 0 | 6 | 0 | 0 | 8 | 0 | 0 | 0 | 0 | 0 |
| Xylomelasma | 0 | 10 | 0 | 0 | 0 | 0 | 12 | 3 | 0 | 4 | 0 | 0 | 2 | 3 | 0 | 0 |
| Ustilaginoidea | 2 | 0 | 3 | 0 | 0 | 0 | 22 | 0 | 5 | 33 | 0 | 0 | 2 | 4 | 0 | 0 |
| Botrytis | 0 | 0 | 0 | 0 | 0 | 0 | 6 | 7 | 0 | 0 | 0 | 0 | 8 | 0 | 0 | 0 |
| Urocentrum | 0 | 20 | 459 | 0 | 0 | 0 | 0 | 0 | 6 | 0 | 0 | 0 | 0 | 0 | 0 | 0 |
| Biatriospora | 0 | 0 | 0 | 5 | 0 | 0 | 0 | 0 | 0 | 0 | 0 | 0 | 16 | 0 | 0 | 0 |
| Annulohypoxylon | 0 | 0 | 0 | 0 | 0 | 0 | 0 | 0 | 0 | 0 | 0 | 0 | 5 | 3 | 0 | 0 |
| Postia | 0 | 0 | 0 | 0 | 0 | 0 | 13 | 0 | 0 | 0 | 0 | 0 | 5 | 0 | 3 | 0 |
| Plasmodiophora | 5 | 0 | 0 | 0 | 0 | 0 | 3 | 0 | 0 | 0 | 0 | 0 | 6 | 0 | 0 | 0 |
| Geastrum | 0 | 0 | 0 | 4 | 13 | 0 | 0 | 0 | 0 | 0 | 0 | 0 | 0 | 0 | 0 | 0 |
| Geosmithia | 3 | 0 | 0 | 3 | 0 | 0 | 0 | 0 | 0 | 0 | 0 | 0 | 0 | 0 | 0 | 0 |
| Gibberella | 0 | 0 | 3 | 38 | 0 | 0 | 0 | 0 | 0 | 0 | 0 | 0 | 4 | 0 | 0 | 0 |
| Parauronema | 0 | 10 | 0 | 0 | 0 | 9 | 0 | 0 | 0 | 0 | 0 | 0 | 0 | 0 | 0 | 0 |
| Eunapius | 0 | 0 | 0 | 0 | 35 | 0 | 0 | 0 | 0 | 0 | 0 | 0 | 0 | 0 | 0 | 0 |
| Stilbella | 0 | 30 | 0 | 8 | 0 | 0 | 0 | 0 | 0 | 0 | 0 | 0 | 0 | 0 | 0 | 0 |
| Stagonospora | 0 | 0 | 0 | 0 | 0 | 0 | 0 | 6 | 0 | 0 | 0 | 0 | 9 | 0 | 20 | 0 |
| Setophaeosphaeria | 0 | 13 | 10 | 0 | 39 | 19 | 21 | 0 | 0 | 0 | 0 | 0 | 0 | 0 | 0 | 20 |
| Hongkongmyces | 151 | 102 | 29 | 245 | 71 | 34 | 42 | 13 | 8 | 0 | 99 | 0 | 31 | 76 | 5 | 80 |
| Hyaloraphidium | 0 | 0 | 0 | 0 | 0 | 0 | 0 | 0 | 0 | 0 | 0 | 0 | 0 | 16 | 0 | 0 |
| Pulvinula | 9 | 0 | 0 | 13 | 0 | 0 | 3 | 0 | 0 | 0 | 0 | 0 | 0 | 0 | 0 | 0 |
| Granulobasidium | 0 | 0 | 0 | 0 | 0 | 0 | 0 | 3 | 0 | 0 | 0 | 0 | 18 | 0 | 0 | 0 |
| Gymnoascus | 0 | 0 | 0 | 0 | 0 | 0 | 0 | 3 | 0 | 0 | 0 | 0 | 4 | 0 | 0 | 0 |
| Sinodiaptomus | 0 | 0 | 0 | 0 | 0 | 0 | 0 | 0 | 0 | 2295 | 4 | 0 | 0 | 0 | 0 | 2 |
| Craspedacusta | 4 | 0 | 0 | 0 | 0 | 0 | 0 | 0 | 0 | 0 | 2 | 0 | 0 | 0 | 0 | 0 |
| Delfinachytrium | 0 | 0 | 8 | 0 | 0 | 0 | 0 | 0 | 0 | 0 | 2 | 0 | 0 | 0 | 0 | 0 |
| Diaporthe | 0 | 0 | 0 | 0 | 0 | 0 | 0 | 0 | 0 | 0 | 5 | 0 | 6 | 0 | 0 | 0 |
| Cystofilobasidium | 0 | 0 | 0 | 0 | 0 | 2 | 44 | 0 | 0 | 0 | 0 | 0 | 0 | 0 | 0 | 0 |
| Thaumatomonas | 0 | 0 | 0 | 0 | 0 | 0 | 0 | 0 | 4 | 3 | 0 | 0 | 0 | 0 | 0 | 0 |
| Cyberlindnera | 12 | 3 | 0 | 0 | 0 | 0 | 4 | 28 | 5 | 5 | 0 | 0 | 3 | 167 | 45 | 32 |
| Diaxonella | 0 | 14 | 0 | 0 | 0 | 0 | 0 | 0 | 0 | 14 | 0 | 0 | 0 | 0 | 0 | 0 |
| Tachysoma | 0 | 0 | 0 | 51 | 0 | 0 | 7 | 0 | 0 | 0 | 0 | 0 | 0 | 57 | 0 | 0 |
| Pedospumella | 4 | 0 | 0 | 0 | 0 | 0 | 0 | 0 | 0 | 0 | 0 | 0 | 0 | 0 | 2 | 0 |
| Suillus | 0 | 0 | 7 | 0 | 0 | 0 | 0 | 0 | 0 | 3 | 0 | 0 | 10 | 0 | 0 | 0 |
| Dictyosporium | 0 | 0 | 0 | 0 | 0 | 0 | 0 | 0 | 0 | 0 | 0 | 29 | 7 | 5 | 0 | 0 |
| Punctularia | 0 | 4 | 0 | 0 | 0 | 0 | 0 | 0 | 0 | 0 | 0 | 0 | 0 | 0 | 0 | 0 |
| Rasamsonia | 0 | 0 | 0 | 0 | 0 | 0 | 0 | 0 | 0 | 0 | 0 | 0 | 0 | 0 | 12 | 0 |
| Rhizophagus | 0 | 0 | 0 | 0 | 0 | 3 | 0 | 0 | 0 | 0 | 0 | 0 | 0 | 0 | 0 | 0 |
| Rhizophlyctis | 0 | 0 | 0 | 107 | 0 | 0 | 0 | 0 | 0 | 0 | 0 | 0 | 0 | 0 | 0 | 0 |
| Pseudaleuria | 0 | 0 | 0 | 0 | 0 | 0 | 0 | 0 | 3 | 0 | 0 | 0 | 0 | 0 | 0 | 0 |
| Radulidium | 0 | 0 | 0 | 0 | 0 | 0 | 0 | 0 | 0 | 0 | 0 | 0 | 6 | 0 | 0 | 0 |
| Prorocentrum | 0 | 0 | 0 | 0 | 0 | 0 | 0 | 16 | 0 | 9 | 0 | 0 | 5 | 8 | 0 | 0 |
| Pseudourostyla | 0 | 0 | 0 | 0 | 0 | 0 | 0 | 0 | 0 | 0 | 0 | 0 | 0 | 0 | 9 | 0 |
| Protospathidium | 0 | 0 | 0 | 67 | 0 | 0 | 0 | 0 | 0 | 0 | 0 | 0 | 0 | 0 | 0 | 0 |
| Pseudomerulius | 0 | 0 | 0 | 0 | 0 | 0 | 0 | 7 | 0 | 0 | 0 | 0 | 0 | 0 | 0 | 0 |
| Pseudogymnoascus | 0 | 0 | 0 | 0 | 0 | 0 | 0 | 0 | 0 | 0 | 0 | 0 | 54 | 0 | 20 | 0 |
| Psilocybe | 0 | 0 | 0 | 0 | 0 | 0 | 0 | 0 | 7 | 0 | 0 | 0 | 0 | 0 | 0 | 0 |
| Rachicladosporium | 0 | 0 | 0 | 0 | 0 | 0 | 0 | 0 | 0 | 0 | 0 | 0 | 4 | 0 | 0 | 0 |
| Tingoldiago | 0 | 0 | 6 | 0 | 0 | 0 | 0 | 0 | 0 | 0 | 0 | 0 | 0 | 0 | 0 | 0 |
| Tilletiopsis | 0 | 0 | 3 | 0 | 0 | 0 | 0 | 0 | 0 | 0 | 0 | 0 | 0 | 0 | 0 | 0 |
| Tilletia | 0 | 0 | 0 | 6 | 0 | 0 | 0 | 0 | 0 | 0 | 0 | 0 | 0 | 0 | 0 | 0 |
| Tricholoma | 0 | 0 | 0 | 0 | 0 | 0 | 0 | 0 | 0 | 0 | 0 | 0 | 3 | 0 | 0 | 0 |
| Trametes | 0 | 7 | 0 | 0 | 0 | 0 | 0 | 0 | 0 | 0 | 0 | 0 | 0 | 0 | 0 | 0 |
| Tolypocladium | 0 | 0 | 0 | 0 | 0 | 0 | 0 | 0 | 0 | 0 | 0 | 0 | 5 | 0 | 0 | 0 |
| Thelebolus | 0 | 0 | 14 | 0 | 0 | 0 | 0 | 0 | 0 | 0 | 0 | 0 | 0 | 0 | 0 | 0 |
| Thanatephorus | 0 | 0 | 0 | 0 | 0 | 0 | 0 | 0 | 0 | 0 | 0 | 0 | 0 | 126 | 0 | 0 |
| Tausonia | 0 | 0 | 0 | 0 | 0 | 0 | 0 | 0 | 0 | 0 | 0 | 0 | 21 | 0 | 0 | 0 |
| Thielaviopsis | 0 | 0 | 0 | 0 | 0 | 0 | 3 | 0 | 0 | 0 | 0 | 0 | 0 | 0 | 0 | 0 |
| Thermomyces | 0 | 0 | 0 | 0 | 0 | 6 | 0 | 0 | 0 | 0 | 0 | 0 | 0 | 0 | 0 | 0 |
| Thelephora | 0 | 0 | 0 | 44 | 0 | 0 | 9 | 0 | 0 | 0 | 0 | 0 | 0 | 0 | 0 | 0 |
| Xenasmatella | 0 | 0 | 0 | 0 | 0 | 0 | 0 | 0 | 0 | 0 | 0 | 0 | 10 | 0 | 0 | 0 |
| Xenasma | 0 | 0 | 0 | 0 | 0 | 0 | 8 | 0 | 0 | 0 | 0 | 0 | 0 | 0 | 0 | 0 |
| Wallrothiella | 0 | 0 | 0 | 0 | 0 | 0 | 0 | 0 | 0 | 4 | 0 | 0 | 0 | 0 | 0 | 0 |
| Yamadazyma | 0 | 0 | 0 | 0 | 0 | 0 | 8 | 0 | 0 | 0 | 0 | 0 | 0 | 0 | 0 | 0 |
| Xylaria | 0 | 0 | 0 | 0 | 0 | 0 | 6 | 0 | 0 | 0 | 0 | 0 | 0 | 0 | 0 | 0 |
| Xepicula | 0 | 0 | 0 | 0 | 0 | 0 | 0 | 0 | 4 | 0 | 0 | 0 | 0 | 0 | 0 | 0 |
| Udeniozyma | 0 | 0 | 0 | 0 | 0 | 0 | 0 | 0 | 0 | 0 | 0 | 0 | 0 | 0 | 3 | 0 |
| Tryblidiopsis | 0 | 0 | 0 | 0 | 0 | 0 | 0 | 0 | 0 | 0 | 0 | 0 | 4 | 0 | 0 | 0 |
| Tricula | 0 | 11 | 0 | 0 | 0 | 0 | 0 | 0 | 0 | 0 | 0 | 0 | 0 | 0 | 0 | 0 |
| Vahlkampfia | 0 | 0 | 0 | 2 | 0 | 0 | 0 | 0 | 0 | 0 | 0 | 0 | 0 | 0 | 5 | 0 |
| Uronemella | 0 | 0 | 0 | 0 | 0 | 0 | 0 | 0 | 0 | 0 | 5 | 0 | 0 | 0 | 0 | 0 |
| Umbilicaria | 0 | 0 | 0 | 0 | 0 | 0 | 0 | 0 | 0 | 0 | 0 | 0 | 10 | 0 | 0 | 0 |
| Symmetrospora | 0 | 0 | 0 | 0 | 0 | 0 | 0 | 0 | 0 | 0 | 0 | 7 | 0 | 0 | 0 | 0 |
| Saccharomycopsis | 0 | 0 | 0 | 0 | 0 | 0 | 0 | 0 | 0 | 0 | 0 | 6 | 0 | 0 | 0 | 0 |
| Russula | 0 | 0 | 7 | 0 | 0 | 0 | 0 | 0 | 0 | 0 | 0 | 0 | 0 | 0 | 0 | 0 |
| Ripartites | 0 | 0 | 0 | 0 | 0 | 0 | 0 | 0 | 0 | 0 | 0 | 0 | 3 | 0 | 0 | 0 |
| Sarcopodium | 10 | 0 | 3 | 0 | 0 | 11 | 0 | 0 | 0 | 0 | 8 | 0 | 0 | 0 | 0 | 0 |
| Sampaiozyma | 0 | 0 | 0 | 0 | 0 | 0 | 0 | 0 | 7 | 0 | 0 | 0 | 0 | 0 | 0 | 0 |
| Sagenomella | 0 | 0 | 0 | 0 | 0 | 0 | 6 | 0 | 0 | 0 | 0 | 0 | 5 | 0 | 6 | 0 |
| Rhizoscyphus | 0 | 0 | 0 | 0 | 0 | 0 | 0 | 0 | 0 | 0 | 0 | 0 | 10 | 0 | 0 | 0 |
| Rhizopus | 0 | 0 | 0 | 0 | 0 | 0 | 0 | 0 | 0 | 0 | 0 | 0 | 7 | 0 | 0 | 0 |
| Rhizophydium | 0 | 0 | 0 | 0 | 0 | 0 | 0 | 0 | 0 | 0 | 0 | 0 | 0 | 0 | 0 | 4 |
| Rigidoporus | 0 | 0 | 0 | 0 | 0 | 0 | 0 | 0 | 0 | 0 | 0 | 0 | 4 | 0 | 0 | 0 |
| Rhyacodrilus | 0 | 0 | 0 | 0 | 0 | 0 | 0 | 0 | 0 | 0 | 2 | 0 | 0 | 0 | 0 | 0 |
| Rhizosphaera | 0 | 0 | 0 | 0 | 0 | 0 | 0 | 0 | 0 | 0 | 0 | 14 | 0 | 0 | 0 | 0 |
| Spizellomyces | 4 | 0 | 0 | 0 | 0 | 0 | 0 | 0 | 0 | 0 | 0 | 0 | 0 | 0 | 0 | 0 |
| Spiromastix | 0 | 0 | 0 | 0 | 0 | 0 | 0 | 0 | 0 | 0 | 0 | 0 | 0 | 0 | 4 | 0 |
| Sodiomyces | 0 | 0 | 0 | 0 | 0 | 0 | 0 | 0 | 0 | 0 | 0 | 0 | 0 | 0 | 6 | 0 |
| Stenostomum | 0 | 14 | 0 | 0 | 0 | 0 | 0 | 0 | 0 | 0 | 0 | 0 | 0 | 0 | 0 | 0 |
| Stachybotrys | 12 | 0 | 0 | 8 | 0 | 16 | 0 | 16 | 14 | 0 | 0 | 0 | 8 | 9 | 17 | 50 |
| Sporendonema | 0 | 0 | 0 | 0 | 0 | 0 | 0 | 0 | 0 | 24 | 0 | 0 | 0 | 0 | 0 | 0 |
| Septoria | 0 | 0 | 2 | 0 | 0 | 0 | 0 | 0 | 0 | 0 | 0 | 0 | 0 | 0 | 0 | 0 |
| Segregatospumella | 0 | 0 | 0 | 10 | 5 | 0 | 0 | 0 | 0 | 0 | 0 | 0 | 0 | 0 | 0 | 16 |
| Schizophyllum | 0 | 0 | 0 | 0 | 0 | 0 | 0 | 6 | 0 | 0 | 0 | 0 | 0 | 0 | 0 | 0 |
| Smardaea | 0 | 0 | 0 | 0 | 0 | 0 | 0 | 0 | 0 | 0 | 0 | 0 | 0 | 0 | 4 | 0 |
| Setoseptoria | 0 | 0 | 0 | 0 | 0 | 0 | 0 | 0 | 0 | 0 | 0 | 0 | 0 | 3 | 0 | 0 |
| Setophoma | 0 | 0 | 47 | 0 | 0 | 0 | 0 | 0 | 0 | 0 | 0 | 0 | 0 | 0 | 0 | 0 |
| Cryptosporiopsis | 0 | 0 | 0 | 0 | 0 | 0 | 0 | 0 | 0 | 0 | 0 | 0 | 4 | 0 | 0 | 0 |
| Cylindrocarpon | 0 | 0 | 0 | 87 | 0 | 0 | 0 | 0 | 0 | 0 | 0 | 0 | 0 | 0 | 0 | 0 |
| Crepidotus | 0 | 0 | 0 | 0 | 0 | 0 | 0 | 0 | 0 | 0 | 0 | 0 | 8 | 0 | 0 | 0 |
| Cryptocaryon | 0 | 0 | 0 | 0 | 0 | 0 | 0 | 210 | 0 | 0 | 0 | 0 | 0 | 0 | 0 | 0 |
| Dactylella | 0 | 0 | 4 | 0 | 0 | 0 | 0 | 0 | 0 | 0 | 0 | 0 | 0 | 0 | 0 | 0 |
| Dactylogyrus | 7 | 0 | 0 | 0 | 0 | 0 | 0 | 0 | 0 | 0 | 0 | 0 | 0 | 0 | 0 | 0 |
| Cyphellophora | 0 | 0 | 0 | 6 | 0 | 0 | 0 | 0 | 0 | 0 | 0 | 0 | 0 | 0 | 0 | 0 |
| Cystotheca | 0 | 0 | 42 | 0 | 0 | 0 | 0 | 0 | 0 | 0 | 0 | 0 | 0 | 0 | 0 | 0 |
| Cordana | 0 | 17 | 0 | 0 | 0 | 0 | 0 | 0 | 0 | 0 | 0 | 0 | 0 | 0 | 0 | 0 |
| Citeromyces | 0 | 0 | 0 | 0 | 0 | 0 | 22 | 0 | 0 | 0 | 0 | 0 | 0 | 0 | 0 | 0 |
| Clydaea | 0 | 0 | 0 | 0 | 0 | 0 | 0 | 0 | 0 | 0 | 0 | 0 | 0 | 0 | 0 | 11 |
| Chlorophyllum | 0 | 0 | 0 | 0 | 0 | 0 | 0 | 6 | 0 | 0 | 0 | 0 | 0 | 0 | 0 | 0 |
| Chondrostereum | 0 | 2 | 0 | 0 | 0 | 0 | 0 | 0 | 0 | 0 | 0 | 0 | 0 | 0 | 0 | 0 |
| Conticribra | 0 | 0 | 0 | 0 | 0 | 6 | 0 | 2 | 0 | 0 | 0 | 0 | 0 | 0 | 0 | 0 |
| Coralloidiomyces | 0 | 0 | 0 | 0 | 0 | 0 | 0 | 0 | 0 | 0 | 0 | 0 | 4 | 0 | 0 | 0 |
| Coleophoma | 0 | 0 | 0 | 0 | 0 | 0 | 0 | 0 | 0 | 0 | 0 | 0 | 2 | 0 | 0 | 0 |
| Collophora | 0 | 0 | 0 | 0 | 0 | 0 | 0 | 0 | 0 | 0 | 0 | 0 | 2 | 0 | 0 | 0 |
| Euplotes | 0 | 0 | 0 | 0 | 0 | 8 | 0 | 0 | 0 | 0 | 0 | 0 | 0 | 0 | 0 | 0 |
| Filobasidium | 0 | 0 | 0 | 5 | 0 | 0 | 0 | 0 | 0 | 0 | 4 | 0 | 0 | 0 | 0 | 0 |
| Endosporium | 0 | 0 | 0 | 12 | 0 | 0 | 0 | 0 | 0 | 0 | 0 | 0 | 0 | 0 | 0 | 0 |
| Erysiphe | 0 | 0 | 0 | 0 | 0 | 0 | 0 | 0 | 0 | 0 | 0 | 0 | 18 | 0 | 0 | 0 |
| Fontanospora | 0 | 0 | 0 | 0 | 0 | 0 | 0 | 0 | 0 | 0 | 0 | 0 | 4 | 0 | 0 | 0 |
| Fusicolla | 0 | 0 | 0 | 0 | 0 | 3 | 0 | 0 | 6 | 0 | 0 | 0 | 38 | 0 | 0 | 0 |
| Filosporella | 0 | 0 | 0 | 71 | 0 | 0 | 0 | 0 | 2 | 0 | 0 | 0 | 0 | 0 | 2 | 0 |
| Fimetariella | 0 | 0 | 0 | 0 | 0 | 0 | 0 | 0 | 0 | 0 | 0 | 0 | 3 | 0 | 0 | 0 |
| Dothiorella | 0 | 0 | 0 | 0 | 0 | 0 | 0 | 0 | 0 | 0 | 0 | 0 | 4 | 0 | 0 | 0 |
| Diacyclops | 0 | 0 | 0 | 0 | 0 | 0 | 0 | 5 | 0 | 0 | 0 | 0 | 0 | 0 | 0 | 0 |
| Didymella | 0 | 0 | 0 | 0 | 0 | 0 | 0 | 0 | 6 | 0 | 0 | 0 | 13 | 0 | 0 | 0 |
| Davidhawksworthia | 0 | 0 | 0 | 0 | 0 | 0 | 0 | 0 | 0 | 0 | 0 | 0 | 5 | 0 | 0 | 0 |
| Debaryomyces | 0 | 0 | 0 | 0 | 0 | 0 | 0 | 13 | 0 | 0 | 0 | 0 | 0 | 0 | 0 | 0 |
| Dipodascus | 0 | 0 | 0 | 0 | 0 | 0 | 10 | 0 | 0 | 0 | 0 | 0 | 0 | 11 | 0 | 0 |
| Diversispora | 0 | 0 | 0 | 0 | 0 | 0 | 0 | 0 | 0 | 0 | 0 | 0 | 3 | 0 | 0 | 0 |
| Dileptus | 0 | 0 | 0 | 0 | 0 | 0 | 0 | 0 | 6 | 0 | 0 | 0 | 0 | 6 | 0 | 0 |
| Dinemasporium | 0 | 0 | 0 | 0 | 0 | 3 | 0 | 0 | 0 | 0 | 0 | 0 | 0 | 0 | 0 | 0 |
| Aquastroma | 0 | 0 | 0 | 0 | 0 | 0 | 2 | 0 | 0 | 0 | 0 | 0 | 0 | 0 | 0 | 0 |
| Arachnomyces | 0 | 0 | 0 | 0 | 0 | 0 | 0 | 0 | 5 | 0 | 0 | 0 | 2 | 0 | 0 | 0 |
| Aquadiscula | 0 | 0 | 0 | 0 | 0 | 0 | 0 | 0 | 0 | 0 | 0 | 0 | 0 | 0 | 16 | 0 |
| Aquapeziza | 0 | 0 | 0 | 0 | 0 | 0 | 0 | 0 | 0 | 28 | 0 | 0 | 0 | 0 | 0 | 0 |
| Arthrinium | 0 | 0 | 3 | 0 | 0 | 0 | 0 | 0 | 0 | 0 | 0 | 0 | 0 | 0 | 0 | 0 |
| Arxiella | 0 | 0 | 0 | 0 | 0 | 0 | 0 | 0 | 0 | 0 | 0 | 0 | 0 | 0 | 10 | 0 |
| Archaeorhizomyces | 0 | 0 | 0 | 0 | 0 | 0 | 0 | 0 | 0 | 0 | 0 | 0 | 9 | 0 | 0 | 0 |
| Aristerostoma | 3 | 0 | 0 | 0 | 0 | 0 | 0 | 0 | 0 | 0 | 0 | 0 | 0 | 0 | 0 | 0 |
| Apiotrichum | 0 | 0 | 0 | 0 | 0 | 0 | 0 | 7 | 0 | 0 | 0 | 0 | 0 | 0 | 0 | 0 |
| Aequabiliella | 0 | 0 | 0 | 3 | 0 | 0 | 0 | 0 | 0 | 0 | 0 | 0 | 0 | 0 | 0 | 0 |
| Aleuria | 0 | 0 | 0 | 0 | 0 | 0 | 0 | 0 | 0 | 0 | 0 | 0 | 0 | 4 | 0 | 0 |
| Achroiostachys | 0 | 0 | 2 | 0 | 0 | 0 | 0 | 0 | 0 | 0 | 0 | 0 | 0 | 0 | 0 | 0 |
| Actinomucor | 0 | 0 | 10 | 0 | 0 | 0 | 0 | 0 | 0 | 0 | 0 | 0 | 0 | 0 | 0 | 0 |
| Angulomyces | 0 | 0 | 0 | 0 | 0 | 0 | 0 | 0 | 0 | 0 | 0 | 0 | 0 | 3 | 0 | 0 |
| Antennariella | 0 | 0 | 0 | 0 | 0 | 0 | 0 | 0 | 0 | 0 | 0 | 10 | 0 | 0 | 0 | 0 |
| Alogomyces | 0 | 0 | 0 | 0 | 0 | 0 | 0 | 0 | 0 | 0 | 0 | 8 | 0 | 0 | 0 | 0 |
| Alphamyces | 0 | 0 | 0 | 0 | 0 | 0 | 0 | 0 | 0 | 0 | 0 | 0 | 0 | 4 | 0 | 0 |
| Celosporium | 0 | 4 | 0 | 0 | 0 | 0 | 0 | 0 | 0 | 0 | 0 | 0 | 0 | 0 | 0 | 0 |
| Cenococcum | 0 | 0 | 0 | 0 | 0 | 0 | 0 | 0 | 0 | 0 | 0 | 0 | 4 | 0 | 0 | 0 |
| Camarosporium | 11 | 0 | 0 | 0 | 0 | 0 | 0 | 0 | 0 | 0 | 0 | 0 | 0 | 0 | 0 | 0 |
| Catenulifera | 0 | 0 | 0 | 0 | 0 | 0 | 0 | 0 | 0 | 0 | 0 | 0 | 4 | 0 | 0 | 0 |
| Cercophora | 0 | 0 | 0 | 0 | 0 | 8 | 0 | 0 | 0 | 0 | 0 | 0 | 0 | 0 | 0 | 0 |
| Chloridium | 0 | 0 | 0 | 0 | 0 | 0 | 0 | 0 | 0 | 0 | 0 | 0 | 8 | 0 | 0 | 0 |
| Cephalosporium | 0 | 0 | 0 | 0 | 0 | 0 | 0 | 0 | 0 | 0 | 0 | 0 | 0 | 3 | 0 | 0 |
| Cephalotrichiella | 0 | 0 | 0 | 9 | 0 | 0 | 0 | 0 | 0 | 0 | 0 | 0 | 11 | 0 | 0 | 0 |
| Calonectria | 0 | 0 | 4 | 0 | 0 | 0 | 0 | 0 | 0 | 0 | 0 | 0 | 0 | 0 | 0 | 0 |
| Basidioascus | 0 | 0 | 0 | 0 | 0 | 0 | 0 | 0 | 0 | 0 | 0 | 0 | 0 | 0 | 2 | 0 |
| Biecheleria | 0 | 0 | 0 | 0 | 0 | 0 | 0 | 0 | 4 | 0 | 0 | 0 | 0 | 0 | 0 | 0 |
| Ascitendus | 10 | 0 | 0 | 0 | 0 | 0 | 0 | 0 | 0 | 0 | 0 | 0 | 0 | 0 | 0 | 0 |
| Bannoa | 0 | 6 | 0 | 0 | 0 | 0 | 0 | 0 | 0 | 0 | 0 | 0 | 0 | 0 | 0 | 0 |
| Buxtonella | 5 | 0 | 0 | 0 | 0 | 0 | 0 | 0 | 0 | 0 | 0 | 0 | 0 | 0 | 0 | 0 |
| Byssochlamys | 0 | 0 | 0 | 0 | 0 | 0 | 0 | 0 | 0 | 0 | 0 | 0 | 0 | 0 | 0 | 10 |
| Bipolaris | 0 | 0 | 0 | 0 | 4 | 0 | 0 | 0 | 0 | 0 | 0 | 0 | 0 | 0 | 0 | 0 |
| Buckleyzyma | 0 | 0 | 0 | 0 | 0 | 0 | 0 | 0 | 0 | 0 | 0 | 0 | 3 | 0 | 0 | 0 |
| Gaertneriomyces | 0 | 0 | 0 | 0 | 0 | 0 | 0 | 0 | 0 | 0 | 63 | 0 | 0 | 0 | 0 | 0 |
| Neosartorya | 0 | 0 | 0 | 0 | 0 | 0 | 0 | 0 | 0 | 0 | 0 | 0 | 0 | 0 | 13 | 0 |
| Neourostylopsis | 0 | 0 | 0 | 0 | 0 | 0 | 34 | 0 | 0 | 0 | 0 | 0 | 0 | 0 | 32 | 69 |
| Neofusicoccum | 9 | 0 | 0 | 0 | 0 | 0 | 0 | 0 | 0 | 0 | 0 | 0 | 0 | 0 | 0 | 0 |
| Neophaeococcomyces | 0 | 0 | 0 | 0 | 0 | 0 | 0 | 0 | 0 | 10 | 0 | 0 | 0 | 0 | 0 | 0 |
| Occultifur | 0 | 0 | 0 | 0 | 0 | 2 | 0 | 0 | 0 | 0 | 0 | 0 | 0 | 0 | 0 | 0 |
| Ophioceras | 0 | 0 | 0 | 0 | 0 | 0 | 0 | 0 | 0 | 0 | 0 | 0 | 7 | 7 | 0 | 0 |
| Nigrograna | 0 | 0 | 0 | 0 | 0 | 0 | 5 | 0 | 0 | 0 | 0 | 0 | 0 | 0 | 0 | 0 |
| Nigrospora | 0 | 0 | 34 | 0 | 0 | 0 | 0 | 0 | 0 | 0 | 0 | 0 | 0 | 0 | 0 | 0 |
| Nemania | 0 | 0 | 0 | 0 | 0 | 0 | 0 | 0 | 0 | 9 | 0 | 0 | 0 | 9 | 0 | 0 |
| Millerozyma | 0 | 0 | 0 | 0 | 0 | 0 | 0 | 0 | 0 | 0 | 0 | 0 | 0 | 0 | 4 | 0 |
| Monocillium | 0 | 0 | 0 | 0 | 0 | 0 | 0 | 0 | 0 | 0 | 0 | 0 | 2 | 0 | 0 | 0 |
| Metschnikowia | 0 | 0 | 0 | 0 | 0 | 0 | 0 | 0 | 0 | 0 | 0 | 0 | 0 | 2 | 0 | 0 |
| Microbotryum | 4 | 0 | 0 | 0 | 0 | 0 | 0 | 0 | 0 | 0 | 0 | 0 | 0 | 0 | 0 | 0 |
| Nassula | 0 | 0 | 0 | 0 | 0 | 0 | 2 | 0 | 0 | 0 | 0 | 0 | 0 | 0 | 0 | 0 |
| Naucoria | 0 | 0 | 0 | 3 | 0 | 0 | 0 | 0 | 0 | 0 | 0 | 0 | 0 | 0 | 0 | 0 |
| Mycosphaerella | 0 | 5 | 0 | 0 | 0 | 0 | 0 | 0 | 0 | 0 | 0 | 0 | 0 | 0 | 0 | 0 |
| Myrothecium | 0 | 0 | 0 | 5 | 0 | 0 | 0 | 0 | 0 | 0 | 0 | 0 | 0 | 0 | 0 | 0 |
| Phialemoniopsis | 0 | 0 | 0 | 0 | 0 | 0 | 0 | 0 | 0 | 0 | 0 | 0 | 3 | 0 | 0 | 0 |
| Phomatospora | 0 | 0 | 0 | 0 | 0 | 0 | 6 | 0 | 0 | 0 | 0 | 0 | 0 | 0 | 0 | 0 |
| Phaeosphaeria | 0 | 0 | 0 | 0 | 0 | 0 | 0 | 0 | 0 | 0 | 0 | 0 | 0 | 0 | 7 | 0 |
| Phanerochaete | 0 | 0 | 0 | 0 | 0 | 0 | 0 | 0 | 0 | 0 | 0 | 0 | 0 | 0 | 13 | 0 |
| Pomacea | 0 | 0 | 0 | 0 | 0 | 0 | 0 | 0 | 0 | 0 | 0 | 0 | 2 | 0 | 0 | 0 |
| Powellomyces | 0 | 0 | 0 | 6 | 0 | 0 | 0 | 44 | 0 | 0 | 0 | 0 | 0 | 0 | 0 | 0 |
| Pilidium | 0 | 0 | 0 | 0 | 25 | 0 | 0 | 0 | 0 | 0 | 0 | 0 | 0 | 0 | 0 | 0 |
| Piloderma | 0 | 0 | 0 | 0 | 0 | 0 | 0 | 0 | 0 | 0 | 0 | 0 | 11 | 0 | 0 | 0 |
| Phaeococcomyces | 0 | 0 | 0 | 0 | 0 | 0 | 0 | 0 | 0 | 0 | 7 | 0 | 0 | 0 | 0 | 0 |
| Parafurgasonia | 0 | 0 | 0 | 0 | 0 | 0 | 0 | 0 | 0 | 0 | 0 | 0 | 0 | 28 | 0 | 0 |
| Paramicrosporidium | 0 | 0 | 0 | 0 | 0 | 12 | 0 | 26 | 0 | 143 | 0 | 0 | 0 | 0 | 0 | 0 |
| Paecilomyces | 0 | 0 | 0 | 0 | 0 | 0 | 0 | 0 | 3 | 0 | 0 | 0 | 0 | 0 | 0 | 0 |
| Paraconiothyrium | 0 | 0 | 0 | 0 | 23 | 0 | 0 | 0 | 0 | 0 | 0 | 0 | 0 | 0 | 0 | 0 |
| Peniophora | 0 | 0 | 0 | 0 | 0 | 0 | 0 | 0 | 0 | 0 | 0 | 0 | 0 | 0 | 9 | 0 |
| Petriella | 0 | 0 | 0 | 0 | 0 | 0 | 0 | 0 | 13 | 0 | 0 | 0 | 0 | 0 | 0 | 0 |
| Paraphysomonas | 0 | 0 | 0 | 0 | 0 | 0 | 0 | 0 | 0 | 0 | 0 | 0 | 0 | 0 | 0 | 3 |
| Paxillus | 0 | 0 | 0 | 0 | 0 | 0 | 0 | 0 | 0 | 0 | 0 | 0 | 3 | 0 | 0 | 0 |
| Humicola | 13 | 0 | 0 | 0 | 0 | 0 | 28 | 0 | 42 | 0 | 0 | 0 | 51 | 0 | 0 | 0 |
| Hymenula | 0 | 0 | 0 | 50 | 0 | 0 | 0 | 0 | 0 | 0 | 0 | 0 | 0 | 0 | 0 | 0 |
| Harpochytrium | 0 | 0 | 0 | 0 | 0 | 0 | 0 | 24 | 0 | 0 | 0 | 0 | 0 | 0 | 0 | 0 |
| Hebeloma | 0 | 0 | 0 | 9 | 0 | 0 | 0 | 0 | 0 | 0 | 0 | 0 | 0 | 0 | 0 | 0 |
| Iodophanus | 442 | 36 | 3 | 29 | 0 | 0 | 0 | 0 | 0 | 0 | 0 | 0 | 0 | 0 | 0 | 0 |
| Keratella | 0 | 0 | 0 | 0 | 0 | 0 | 0 | 0 | 0 | 0 | 25 | 0 | 0 | 0 | 0 | 0 |
| Hyphodontia | 0 | 0 | 0 | 0 | 0 | 0 | 0 | 0 | 0 | 0 | 0 | 0 | 0 | 0 | 31 | 0 |
| Hypomyces | 0 | 0 | 0 | 0 | 0 | 0 | 0 | 0 | 3 | 0 | 7 | 0 | 6 | 0 | 0 | 0 |
| Hannaella | 0 | 0 | 0 | 0 | 0 | 0 | 0 | 0 | 0 | 0 | 0 | 0 | 6 | 0 | 0 | 0 |
| Geomyces | 0 | 0 | 0 | 0 | 0 | 0 | 0 | 0 | 0 | 11 | 0 | 0 | 0 | 0 | 0 | 0 |
| Globulidrilus | 0 | 0 | 0 | 11 | 0 | 0 | 0 | 0 | 0 | 0 | 0 | 0 | 0 | 0 | 0 | 0 |
| Gaeumannomyces | 3 | 0 | 0 | 3 | 0 | 0 | 0 | 175 | 54 | 30 | 0 | 0 | 20 | 226 | 0 | 0 |
| Ganoderma | 0 | 0 | 0 | 0 | 0 | 0 | 0 | 0 | 0 | 0 | 0 | 15 | 0 | 0 | 0 | 0 |
| Gymnostellatospora | 0 | 0 | 0 | 0 | 0 | 0 | 0 | 0 | 0 | 0 | 0 | 0 | 7 | 0 | 0 | 0 |
| Hamigera | 0 | 0 | 0 | 0 | 0 | 0 | 0 | 0 | 0 | 8 | 0 | 0 | 4 | 0 | 0 | 0 |
| Gonapodya | 0 | 0 | 0 | 0 | 0 | 0 | 0 | 0 | 0 | 0 | 0 | 0 | 11 | 0 | 0 | 0 |
| Gymnascella | 0 | 0 | 0 | 0 | 0 | 0 | 0 | 0 | 3 | 0 | 0 | 0 | 0 | 0 | 0 | 0 |
| Lipomyces | 0 | 0 | 0 | 0 | 0 | 0 | 0 | 0 | 0 | 0 | 0 | 0 | 5 | 0 | 0 | 0 |
| Lophodermium | 0 | 0 | 0 | 0 | 0 | 0 | 0 | 0 | 0 | 0 | 0 | 0 | 4 | 0 | 0 | 0 |
| Leucosporidium | 0 | 0 | 0 | 101 | 0 | 0 | 9 | 0 | 0 | 0 | 0 | 0 | 0 | 0 | 0 | 0 |
| Leveillula | 0 | 0 | 0 | 0 | 0 | 0 | 0 | 0 | 0 | 0 | 0 | 0 | 2 | 0 | 0 | 0 |
| Metacordyceps | 0 | 0 | 0 | 0 | 0 | 0 | 0 | 0 | 0 | 0 | 0 | 25 | 0 | 0 | 0 | 0 |
| Metanophrys | 0 | 0 | 0 | 0 | 0 | 0 | 0 | 0 | 0 | 0 | 8 | 0 | 0 | 22 | 0 | 0 |
| Mariannaea | 0 | 0 | 0 | 0 | 0 | 0 | 0 | 0 | 0 | 0 | 0 | 0 | 2 | 0 | 0 | 0 |
| Marssonina | 0 | 0 | 0 | 0 | 0 | 0 | 8 | 0 | 0 | 0 | 0 | 0 | 0 | 0 | 0 | 0 |
| Leuconeurospora | 0 | 0 | 0 | 0 | 0 | 0 | 6 | 0 | 0 | 0 | 0 | 0 | 0 | 0 | 0 | 0 |
| Lactarius | 0 | 0 | 0 | 0 | 0 | 0 | 0 | 0 | 0 | 0 | 0 | 0 | 2 | 0 | 0 | 0 |
| Lambertella | 0 | 0 | 0 | 0 | 0 | 0 | 0 | 0 | 0 | 0 | 0 | 0 | 12 | 0 | 0 | 0 |
| Lachancea | 0 | 0 | 0 | 0 | 0 | 0 | 6 | 13 | 0 | 0 | 0 | 0 | 0 | 0 | 0 | 0 |
| Lacrymaria | 0 | 0 | 0 | 0 | 0 | 0 | 0 | 0 | 0 | 0 | 0 | 0 | 0 | 0 | 14 | 0 |
| Leptosphaeria | 0 | 0 | 0 | 0 | 0 | 0 | 0 | 0 | 0 | 0 | 0 | 0 | 3 | 0 | 0 | 0 |
| Leptospora | 0 | 0 | 0 | 10 | 0 | 0 | 0 | 0 | 0 | 0 | 0 | 0 | 0 | 0 | 0 | 0 |
| Lecane | 0 | 0 | 0 | 0 | 0 | 0 | 0 | 6 | 0 | 0 | 0 | 0 | 0 | 0 | 0 | 0 |
| Lentinus | 0 | 0 | 0 | 0 | 0 | 0 | 0 | 0 | 0 | 0 | 0 | 24 | 0 | 0 | 0 | 0 |

S3. Fig 3. Bacterial (a) and fungal (b) community structures at the genus level at different sampling points

S3. Fig 3. Bacterial (a) and fungal (b) community structures at the genus level at different sampling points
